# Identification of browning-related microRNAs and their targets reveals complex miRNA-mediated browning regulatory networks in *Luffa cylindrica*

**DOI:** 10.1038/s41598-018-33896-9

**Published:** 2018-11-02

**Authors:** Yuanyuan Xu, Zhe Liu, Lina Lou, Xiaojun Su

**Affiliations:** Institute of Vegetable Crops, Jiangsu Academy of Agricultural Sciences, Jiangsu Key Laboratory for Horticultural Crop Genetic Improvement, Nanjing, 210014 People’s Republic of China

## Abstract

As a non-coding and endogenous small RNA, MicroRNA (miRNA) takes a vital regulatory role in plant growth and development. Long-term storage and processing of many fruits and vegetables, including *Luffa*, are subject to influences from browning, a common post-harvest problem that adversely affects flavor, aroma, and nutritional value. The browning regulatory networks mediated by miRNA, however, remain largely unexplored. For a systematic identification of browning-responsive miRNAs and the targets, we built two RNA libraries from *Luffa* pulps of near-isogenic line, with resistant and sensitive browning characteristics respectively, and then sequenced them using Solexa high-throughput technology. We consequently identified 179 known miRNAs that represent 17 non-conserved miRNA families and 24 conserved families, as well as 84 potential novel miRNAs, among which 16 miRNAs (eight known and eight novel miRNAs) were found to exhibit significant differential expressions and were thus identified as browning-related miRNAs. We then studied those browning-responsive miRNAs and the corresponding targets with RT-qPCR and finally validated their expression patterns. The results revealed that the expression patterns are specific to plant development stages and the miRNAs are identified with 39 target transcripts, which involve in plant development, defense response, transcriptional regulation, and signal transduction. After characterizing these miRNAs and their targets, we propose a browning regulatory network model of miRNA-mediatation in this paper. The findings of the work are helpful for the understanding of miRNA-mediated regulatory mechanisms of browning in *Luffa*, and will facilitate genetic improvement of pulp characteristics in *Luffa*.

## Introduction

The worldwide industries of fruits and vegetables are faced with a challenge, browning, in fruits and vegetables, such as banana, apple, potato, and pear, which have been studied extensively^1^. Regarded as a multi-factor process, browning of fruits and vegetables can be classified into two groups, enzymatic browning and non-enzymatic browning^2–4^. Fruit browning is based on enzymatic browning, which is always the key point of study in the preservation of fruit after picking^5,6^. Studies indicated that all the enzymatic browning occurring in fresh fruits and vegetables is harmful, which can not only affect the flavor and taste, but also result in damage to tissue content of amino acids, reduction of vitamin C content, decreased protein solubility, and serious effects on nutritional quality^1^. A variety of internal and external factors effect fruit senescence and every of them controls a subgroup of senescence related genes, which are regulated by a complex regulatory crosstalk and will play their roles during perception, signal transduction pathway, and downstream response sequentially^7,8^. In recent years, browning has been an important factor for controlling the quality of various fruits and vegetables. How to efficiently control browning in fruits and vegetables has been an important subject that requires urgent study in fruit and vegetable cultivation. Although characterization of some key browning-related genes for their biological functions was already made in many plant species^8–10^, a full understanding of their browning regulation at molecular level, however, is not obtained in some cucurbit crops, including *Luffa*.

As a small RNA of roughly 21 to 24 nucleotides (nt), MicroRNAs (miRNAs) guide targeted mRNA cleavages or translation inhibitions and therefore regulate the gene expressions at transcription and/or post-transcription levels in both animals and plants^11^. miRNA modulates down-stream events through posttranscription repressions in their target genes in many plants^12,13^. Moreover, many miRNAs have an evolutional conservation accross many plant species. Aside from their roles in modulating abiotic stresses, such as drought, salt, cold, and heat stress, recent evidence has indicated that miRNAs also takes effect in plant growing, for example leaf morphogenesis, root and flower development, embryogenesis, and plant senescence^14–17^.

Recently, a sequencing technology with high-throughput called NGS (next-generation sequencing) has assisted in the identification of an increasing number of miRNAs related to browning in several crops^8,9^. PPO (Polyphenol oxidase), an enzyme usually existing in animal and plant, is connected to enzymatic browning and has a dinuclear copper centre^18^. Its expressions connected with miRNAs were uncovered empirically. For example, an interaction between PPO-encoding genes and eight known miRNAs was first revealed and was determined its role in browning controlling. Among the miRNAs, miR482 and miR1448 were detected to have down-regulations along with increases in PPO expression^19^. Some miRNAs were found to effect targeting PPOs in other plants and consequently promote biosynthesis of brown pigment. For example, regulation of browning reactions in rice by osa-miR2923a has been verified^20^. Reduction of PPO gene expressions and enzymatic browning for potatoes by artificial miRNAs were also reported. Induced losses of gene functions by artificial microRNAs (amiRNAs) were recorded, which was to prevent browning plant tissues from causing injuries or damages. amiRNAs were produced, for example, in down-regulating PPO genes in plants by modifying miR168a^21,22^. In addition, in potato tuber tissue it was at low levels to express all the identified PPO upstream miRNAs, which could decrease the inhibition of PPO genes and therefore produce brown spots^23^. miR164 was observed to accelerate senescence of its mutants by regulating *ORE1*, the NAC transcription factor^24^. mRNA degradation in auxin response factor ARF2/3/4 and the consequent change in senescence timing were reported to be caused by miR390 triggered production of trans-acting siRNAs from TAS3^25^. A “stay-green” phenotype was reported to be caused by over-expressions of miR319 targeted TCP transcription factors^26,27^. For fruits, on the contrary, only miR156 and miR172 were verified to fine-tune expressions of *CNR* and *AP2a*, two key regulators in tomato fruit ripening^9,28^. These results showed that miRNAs or their combinations with their target genes take a key part in browning related processes and pathways. miRNA-mediated gene expression and the browning regulatory network in *Luffa*, however, is little known.

*Luffa* (2n = 2x = 26), a vegetable of Cucurbitaceae family, is an important annual climbing herbaceous crop worldwide. *Luffa cylindrical*, commonly known as sponge gourd, is a very popular vegetable in China^29^. There are eight species all over the world, wherein mainly two species are cultivated in China, including *Luffa cylindrica* Roem and *Luffa acutangula* Roxb. *Luffa* has therapeutic effects that include fever reduction, dispersing phlegm, and detoxification. Compared with other melons and vegetables, *Luffa* contains higher levels of various nutrients^30^. The pulp of *Luffa* is not only rich in protein, fat, carbohydrate, crude fiber, vitamin, and a variety of mineral elements, but also contains amaroids, mucoid substances, citrulline, xylan, interferon, and saponin, which are contained in ginseng^31^. For their hypolipidemic, cardiac stimulating, antitussive, anti-inflammatory and anti-emetic capabilities, old pulp and seeds of *Luffa* have been applied as medicine substances for long time. In addition, a high antioxidant phenolic substance was verified in *Luffa*, which indicates that it is beneficial for the human health to take *Luffa* in fresh and it is best to have *Luffa* as a food in fresh condiitons after harvest and purchse. It is usually challenged, however, by browning during long storages, which adversely affect the flavor, aroma, and nutritional value^1,6^.

For this reason, it is imperative to elucidate miRNA-directed genetic networks of *Luffa* browning modulation. Through high-throughput sequencing, some miRNAs that can regulate several biological mechanisms have been extensively identified in several plants^17,23,32–35^. For *Luffa*, however, browning-related miRNAs have not been identified or characterized systematically. For this reason, we built two small RNA libraries (JAAS-BR and JAAS-BS) from *Luffa* pulps at 9 days after pollination (DAP) and sequenced them using Solexa sequencing technology. The study was purposed at identification of *Luffa* pulp miRNAs both already known and potential novel, and validation of browning miRNA targeted genes. The result of this study uncovered the regulatory networks in *Luffa* browning mediated by miRNA. It also facilitated understanding of browning mechanisms at molecular level of fruit and vegetables.

## Results

### Sequencing of transcriptomes and small RNAs in *Luffa* pulp

For better understanding of transcription profiles and differences and for the establishment of a comprehensive reference sequencing database of browning phenotypes, we separately built two cDNA libraries from JAAS-BR (browning resistant) and JAAS-BS (browning sensitive) pulps at 9 DAP and then used the Solexa HiSeq 2000 platform to comprehensively sequence the samples, generating 106.6 million total raw reads. Totally 105.4 million clean reads were obtained after removing adaptor contaminants, low-quality tags, and poly(A) tails, from which we then assembled 158,290 contigs using all the high-quality clean reads. We assembled these contigs into 127,914 unigenes (an average length of 923.9 nt and a N50 length of 1,663 nt) after further paired-end annotation and gap filling. We used EST (expressed sequence tag) sequences to integrate our *Luffa* pulp transcriptome library and available GSS (genomic survey sequences) as *Luffa* reference sequences in order to identify the known and novel miRNAs in *Luffa*.

**Figure 1:**
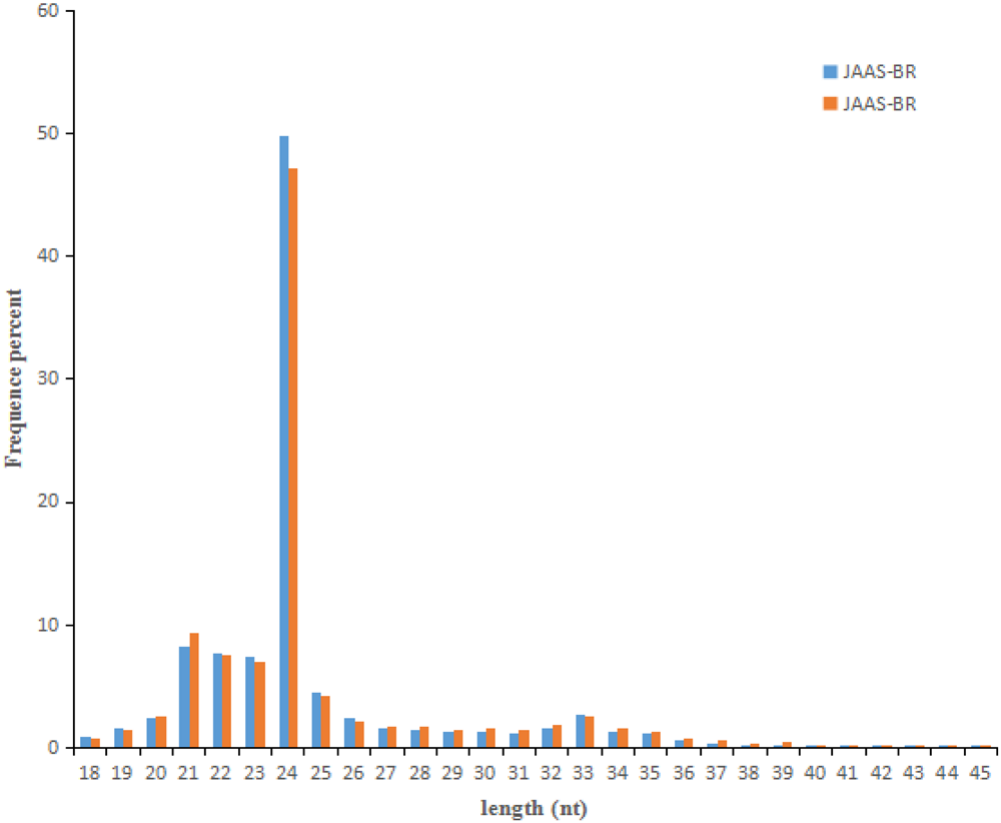
Length distribution and frequency percentage of small RNA sequences in JAAS-BR and JAAS-BS libraries in *Luffa*.

For identification of miRNAs from genetic lines with different browning characteristics, two sRNA libraries, JAAS-BR and JAAS-BS, were respectively constructed from *Luffa* pulps at 9 DAP. In total, in the two libraries, 21,435,789 clean reads representing 11,595,519 sequences were obtained (Table 1 and Fig. S1). We respectively obtained from the above libraries 11,053,385 (representing 5,856,286 unique sequences) and 10,382,404 (representing 5,739,233 unique sequences) clean reads after filtering out the low quality reads, adapter sequences, and contaminants **(**Table 1**)**. In both libraries the sRNA sequences have the length distributed from 18 to 45 nt with 24-nt sRNAs representing the most frequent length, respectively accounting for 49.83% and 47.20% of sRNAs in JAAS-BR and JAAS-BS libraries **(**Fig. 1**)**. Those sequences that matched non-coding sRNAs, including rRNAs, snoRNAs, tRNAs and snRNAs, were all removed by comparing these sRNAs with the Rfam databases and NCBI GenBank (Table S2). Furthermore, for known miRNA identification, we annotated and obtained 8,448 (JAAS-BR) and 7,669 (JAAS-BS) miRNA analogous sequences. The remaining 5,486,351 (JAAS-BR) and 5,280,336 (JAAS-BS) unannotated sRNAs were applied in subsequent analysis of novel miRNAs.

**Table 1:** Statistical analysis of sequencing reads from JAAS-BR and JAAS-BS libraries of *Luffa* pulps.

Category	JAAS-BR		JAAS-BS	
	Count (%)	Percent (%)	Count (%)	Percent (%)
Raw reads	11,620,756		10,826,646	
High quality	11,500,050	100	10,708,503	100
Clean reads	110,533,85	96.12	10,382,404	96.95
3’adapter_null	110,209	0.96	109,402	1.02
Insert null	16,115	0.14	10,427	0.10
5’adapter contaminants	5,571	0.05	4,622	0.04
Smaller than 18nt	314,378	2,73	201,293	1.88
Poly A	392	0.00	355	0.00

### Identification of known miRNAs in *Luffa*

**Figure 2:**
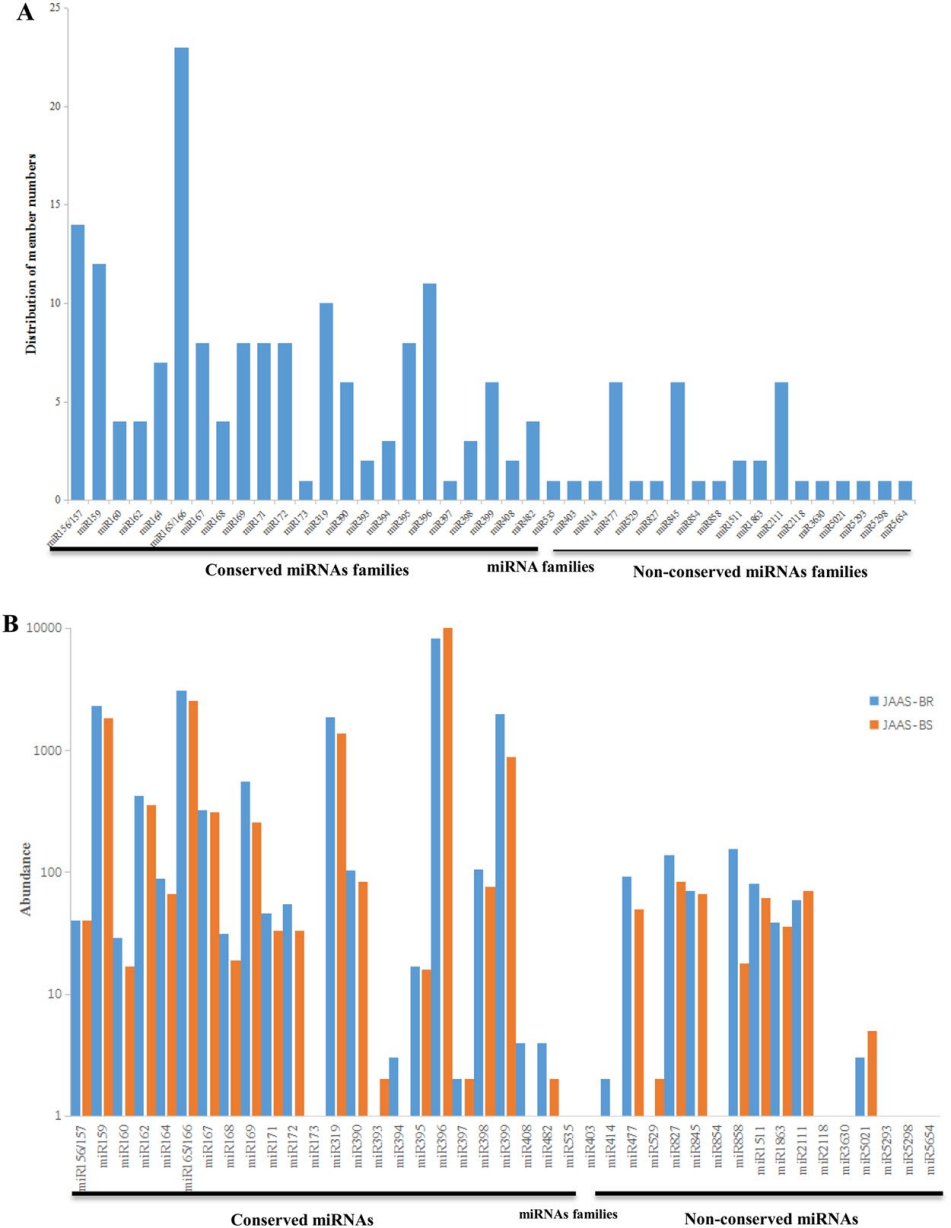
Members and abundances of known miRNA families identified in *Luffa*. (**A**) Distribution of known miRNA family. (**B**) Count of each known miRNA family.

For identification of *Luffa* miRNAs already known corresponding to different browning characteristics, we aligned their sequences with those of the known miRNAs in miRBase 21.0. Ultimately, in the two libraries we identified 158 miRNAs of 24 conserved families (Table 2 and Table S3). In both libraries the conserved miRNA families have very different quantity of members. For example, the miRNA families miR165/166 and miR156/157 had 21 and 14 members respectively; while only one member are there for each of the three miRNA families (miR173, miR397 and miR535) (Table 2 and Fig. 2A). Moreover, in the two libraries we also discovered 34 sequences belonging to 17 non-conserved miRNA families that have less members relative to the conserved (Table 2 and Fig. 2A), where many non-conserved miRNA families (e.g., miR403, miR414, miR529 and miR827) had only one member, whereas three families (miR477, miR845 and miR2111) and two miRNA families (miR1511 and miR1863) had six and two members, respectively.

**Table 2:** Known miRNA families and their abundances identified from JAAS-BR and JAAS-BS libraries.

Family	Members	miRNA reads		Total reads	Ratio
		JAAS-BR	JAAS-BS		(JAAS-BS/JAAS-BR)
**Conserved miRNA**					
miR156/157	14	40	40	80	1.00
miR159	12	2,305	1,840	4,145	0.80
miR160	4	29	17	46	0.59
miR162	4	424	358	782	0.84
miR164	7	88	66	154	0.75
miR165/166	14	2,818	2,359	5,177	0.84
miR167	8	321	309	630	0.96
miR168	3	31	19	50	0.61
miR169	8	553	256	809	0.46
miR171	8	46	33	79	0.72
miR172	8	55	33	88	0.60
miR173	1	0	1	1	—
miR319	10	1,883	1,364	3,247	0.72
miR390	5	104	83	187	0.80
miR393	2	0	2	2	—
miR394	3	3	1	4	0.33
miR395	8	17	16	33	0.94
miR396	11	8,218	9,997	18,215	1.22
miR397	1	2	2	4	1.00
miR398	3	105	76	181	0.72
miR399	6	1,984	883	2,867	0.45
miR408	2	4	0	4	0.00
miR482	4	4	2	6	0.50
miR535	1	1	0	1	0.00
**Non-conserved miRNA**					
miR403	1	0	1	1	—
miR414	1	2	0	2	0.00
miR477	6	92	50	142	0.54
miR529	1	0	2	2	—
miR827	1	138	84	222	0.61
miR845	5	69	67	136	0.97
miR854	1	1	0	1	0.00
miR858	1	156	18	174	0.12
miR894	1	226	184	410	0.81
miR1511	2	80	61	141	0.76
miR1863	2	39	36	75	0.92
miR2111	6	59	71	130	1.20
miR2118	1	1	0	1	0.00
miR3630	1	1	1	2	1.00
miR5021	1	3	5	8	1.67
miR5293	1	1	0	1	0.00
miR5298	1	0	1	1	—
miR5654	1	1	0	1	0.00

In the two libraries the miRNA reads have a relatively stable number with a ratio (JAAS-BS/JAAS-BR) of 0.00 to 1.67 and was thus used in the assessment of miRNA expression levels as an indicator (Table 2 and Fig. 2B). miR396 had a highest number in expressions, respectively with 8,218 and 9,997 copies in JAAS-BR and JAAS-BR. Though miRNAs, like miR162, miR167, miR169, miR390 and miR398, were moderately expressed, some miRNA families, such as miR159, miR165/166, miR319 and miR399, had a relatively high number. However, several miRNA families (e.g., miR394, miR397, miR482 and miR3630) were found to have an extremely low level of expressions in both libraries. Moreover, a few miRNA families, including miR173, miR393, miR408, miR535, miR403, miR414, miR529, miR854, miR2118, miR5293, miR5298, and miR5654, were identified and observed in only one library. Furthermore, within a same miRNA family, the members also had sharply different levels of expressions (Table S3). For example, within miR396, miR396h had a number of 6,736, while miR396e-3p had only one copy. The vast range in expressions within a same family among different members indicates miRNAs’ exact expressions corresponding to specific conditions.

In addition, we studied the known miRNA precursors and predicted their secondary structures in Fig. S2. These miRNA precursors we also studied about their folding free energy (MFE) and length, which are at −59.7 kcal mol^−1^ in average minimal, and ranged from 63 to 605 nt (average 145 nt) (Table S3).

### Identification of potential novel miRNAs candidates in *Luffa*

Novel miRNAs with the characteristic stem ring precursor were selected for annotation^36^. In the present study, we identified totally 84 potential novel miRNAs of 68 novel miRNA families. Among them, we found that 13 novel miRNAs had miRNA*s (complementary miRNA sequences), and many miRNA*s had only one sequence (Table 3; Table S4), partially due to the degration of most miRNA*s in miRNA pathways. The novel mature miRNAs had their main length distributed from 20 nt to 23 nt, with the majority being 21 nt (Table S4). However, only 30 novel miRNAs (35.71%) had over 100 sequenced copies in either of the sRNA libraries. The novel miRNA precursors were from 70 to 361 nt in length, averaged at 169 nt. Moreover, for the 84 novel miRNA precursors, their secondary structures were predicted and identified (Fig. S2), and their MFE is between −121.1 and −18.2 kcal/mol, averaged at −49.87 kcal/mol.

**Table 3:** Novel miRNAs with their complementary miRNA*s identified from JAAS-BR and JAAS-BS libraries.

miRNA	Mature sequences (5′-3′)	Length (nt)	LP	MFE	miRNA reads		Arm	miRNA Location
					JAAS-BR	JAAS-BS		
Lc-miRn1–5p	GGAATGTTGTCTGGTGCGAGA	21	87	−52.9	22	26	5′	TR1186
Lc-miRn1–3p	TCGGACCAGGCTTCATTCCCC	21	87	−52.9	652	472	3′	TR1186
Lc-miRn5-5p	GGAATGTTGTCTGGCTCGAGG	21	154	−47.02	13	6	5′	TR16913
Lc−miRn5-3p	TCGGACCAGGCTTCATTCCCC	21	154	−47.02	433	314	3′	TR16913
Lc-miRn8-5p	AATGCGGTCTGGTTCGAGAGC	21	107	−46.6	10	18	5′	TR22538
Lc-miRn8-3p	TCTCGGACCAGGCTTCATTCT	21	107	−46.6	81	71	3′	TR22538
Lc-miRn10-5p	TGGAGAAGCAGGGCACGTGCTG	22	202	−56.6	40	28	5′	TR30054
Lc-miRn10-3p	TCATGTGCCCCTCTTCGCCATC	22	202	−56.6	1272	471	3′	TR30054
Lc-miRn13-5p	CCACAGCTTTCTTGAACTGCA	21	143	−63.7	100	109	5′	TR3658
Lc-miRn13-3p	GTTCAATAAAGCTGTGGGAAG	21	143	−63.7	26	16	3′	TR3658
Lc-miRn15-5p	TGGCATAGGCTACTTGGAAAC	21	133	−36.8	1508	932	5′	TR47641
Lc-miRn15-3p	TTCCAAGTCCACCCATGCCCGC	22	133	−36.8	1116	864	3′	TR47641
Lc-miRn25b-5p	TTCCACAGCTTTCTTGAACTT	21	166	−61.4	203	255	5′	TR6554
Lc-miRn25b-3p	GTTCAAGAAAGCTGTGGGAGA	21	166	−61.4	1	1	3′	TR6554
Lc-miRn37a-5p	TTCCACAGCTTTCTTGAACTT	21	143	−56.3	198	256	5′	TR9925
Lc-miRn37a-3p	CTCAAGAAAGCTGTGGGACATC	22	143	−56.3	274	229	3′	TR9925
Lc-miRn38-5p	GGAATGTTGGCTGGCTCGAGG	21	146	−44.5	4	7	5′	TR6110
Lc-miRn38-3p	TCGGACCAGGCTTCATTCCCC	21	146	−44.5	644	466	3′	TR6110
Lc-miRn39-5p	TTTTTCCACAGCTTTCTTGAACT	23	132	−32.75	131	213	5′	Chr1
Lc-miRn39-3p	CTCAAGAAAGCTGTGGGAAATTA	23	132	−32.75	426	363	3′	Chr1
Lc-miRn43-5p	CAGAGCTCCTTGAAGTCCAATA	22	236	−86.5	12	11	5′	Chr1
Lc-miRn43-3p	TTTGGATTGAAGGGAGCTCTA	22	236	−86.5	4061	3094	3′	Chr1
Lc-miRn54-5p	TGGAGAAGCAGGGCACGTGCAT	22	135	−66.6	1	4	5′	Chr3
Lc-miRn54-3p	TCATGTGCCCCTCTTCTCCATC	22	135	−66.6	261	111	3′	Chr3
Lc-miRn66-5p	TGGAGAAGCAGGGCACGTGCA	21	96	−40.2	1	3	5′	Chr7
Lc-miRn66-3p	CACGTGCTCCCCTTCTCCAAC	21	96	−40.2	35	13	3′	Chr7

### Identification of browning related miRNAs in *Luffa*

**Figure 3:**
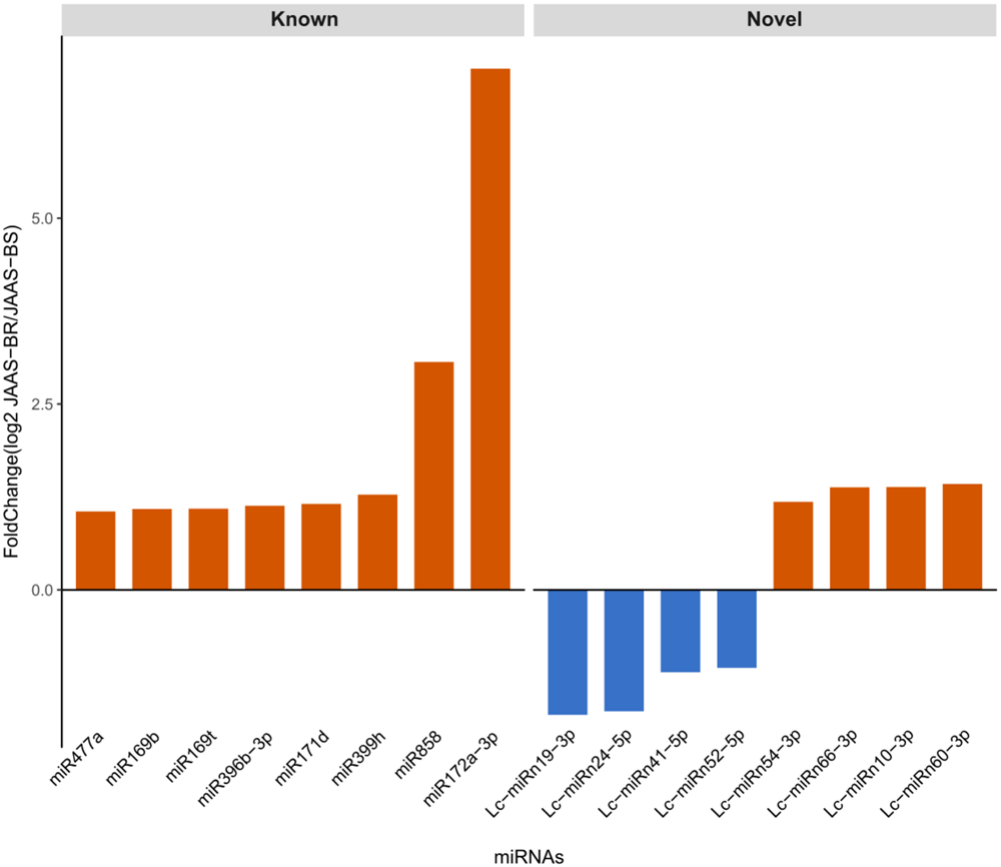
Comparative relative expression of differentially expressed miRNAs. The *y*-axis represents the various differentially expressed miRNAs between *Luffa* with different browning characteristics. The *x*-axis represents the fold change value of each miRNA between the JAAS-BR and JAAS-BS libraries. A represents differentially expressed known miRNAs and B represents differentially expressed novel miRNAs. The miRNAs with positive fold-change values are up-regulated, and the ones with negative fold-change values are down-regulated.

For identification of miRNAs capable of regulating browning in *Luffa*, we carried out differential expression analysis in the JAAS-BR and JAAS-BS libraries on totally 187 known and 84 novel miRNAs. In all, we identified 16 differentially expressed miRNAs, eight known and eight novels, as browning-related (Table S5), which demonstrated very different expressions across the two libraries. Eight known and four novels from the 16 miRNAs were up-related in the JAAS-BR library, and the remaining four novel miRNAs were down-regulated (Fig. 3). Two miRNAs, miR172a-3p (7.01-fold) and Lc-miRn60-3p (1.42-fold), had greatest changes in expression levels. Only one of the sixteen was detected in only one library (Table S5), indicating that this miRNA might display cultivar-specific expressions in *Luffa*. It indicates that in the process of *Luffa* browning differentially regulated miRNAs are significant regulators.

### Target prediction of browning-related miRNAs

**Figure 4:**
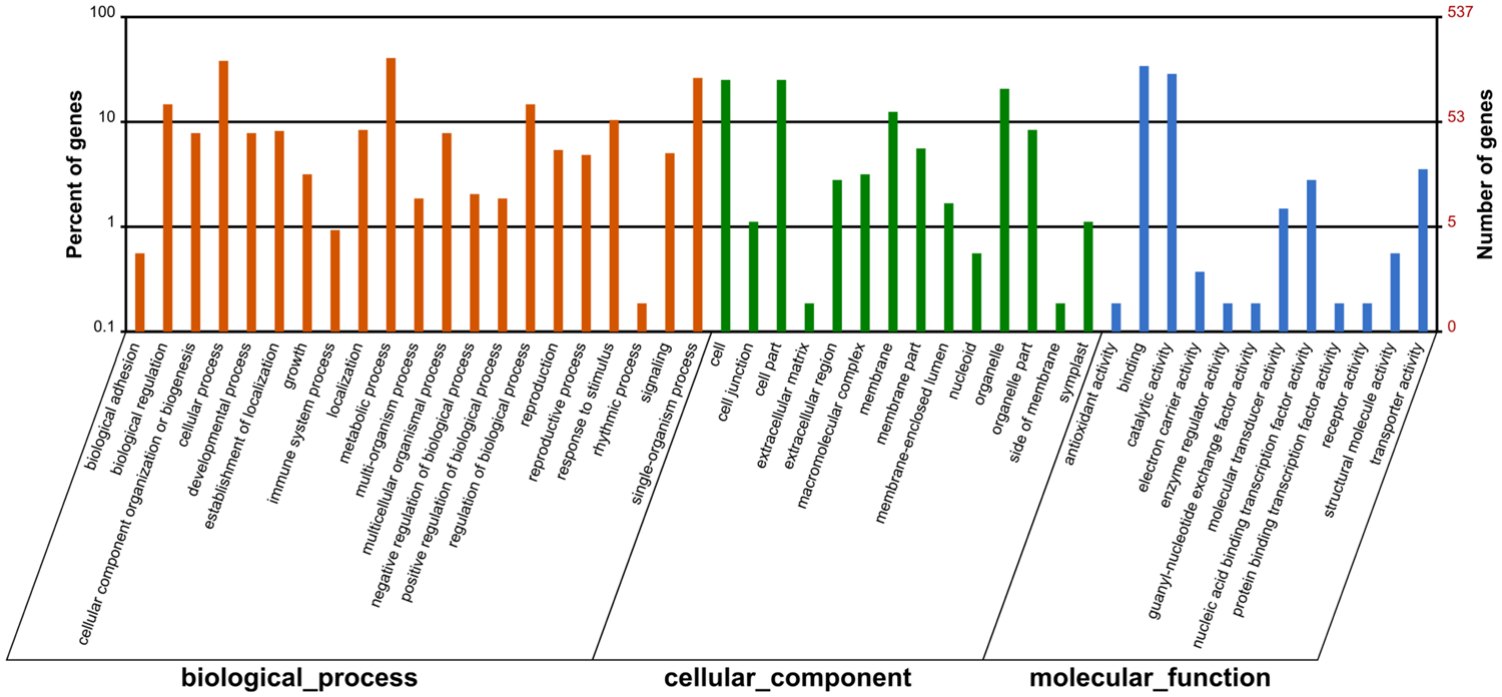
GO classification of target genes for all identified miRNAs in *Luffa*.

**Figure 5:**
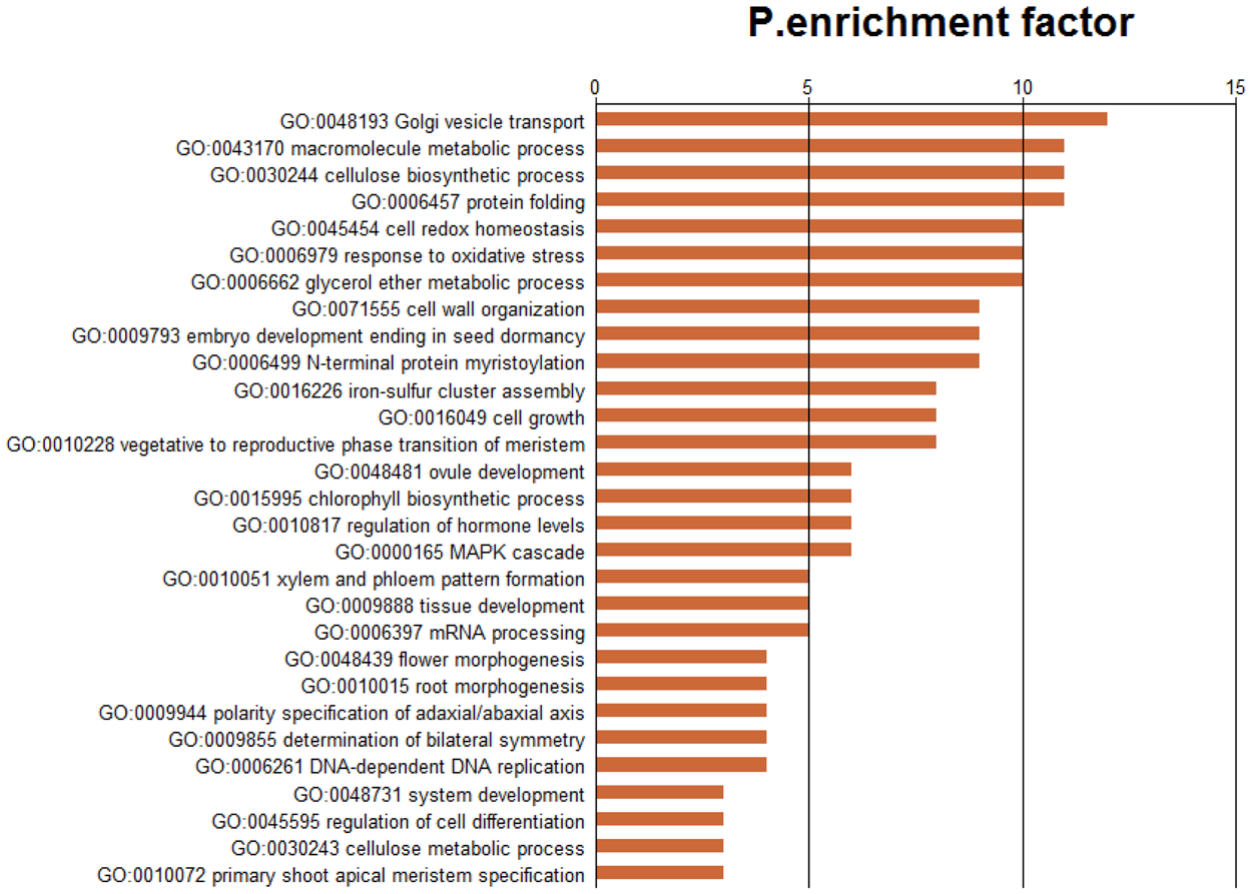
Classification of biological process with GO enrichment multiple analysis.

In functionally characterizing miRNA biological roles, target validation is a prerequisite. In systematic identifying of target transcripts, psRNATarget server is a useful approach^37^. In the present study, for all the identified miRNAs in *Luffa, we* predicted totally 1,209 target transcripts with 2,100 target locations (Table S6). We used Blast2GO analysis to perform annotation on these target transcripts, so as to clarify the miRNA biological functions. We classified the target sequences into three Gene Ontology (GO) categories including cellular components, molecular functions, and biological processes (Fig. 4). With regard to biological processes, the three most abundant GO terms were single-organism process, metabolic process, and cellular process. The three most dominant GO terms in cellular components were organelle, cell part, and cell. Transporter activity, catalytic activity and binding were the three most dominant GO categories for molecular functions. Moreover, the target transcripts were demonstrated, with GO enrichment analysis, to be involved in various cell development, metabolic and oxidation reaction processes (Fig. 5).

**Figure 6:**
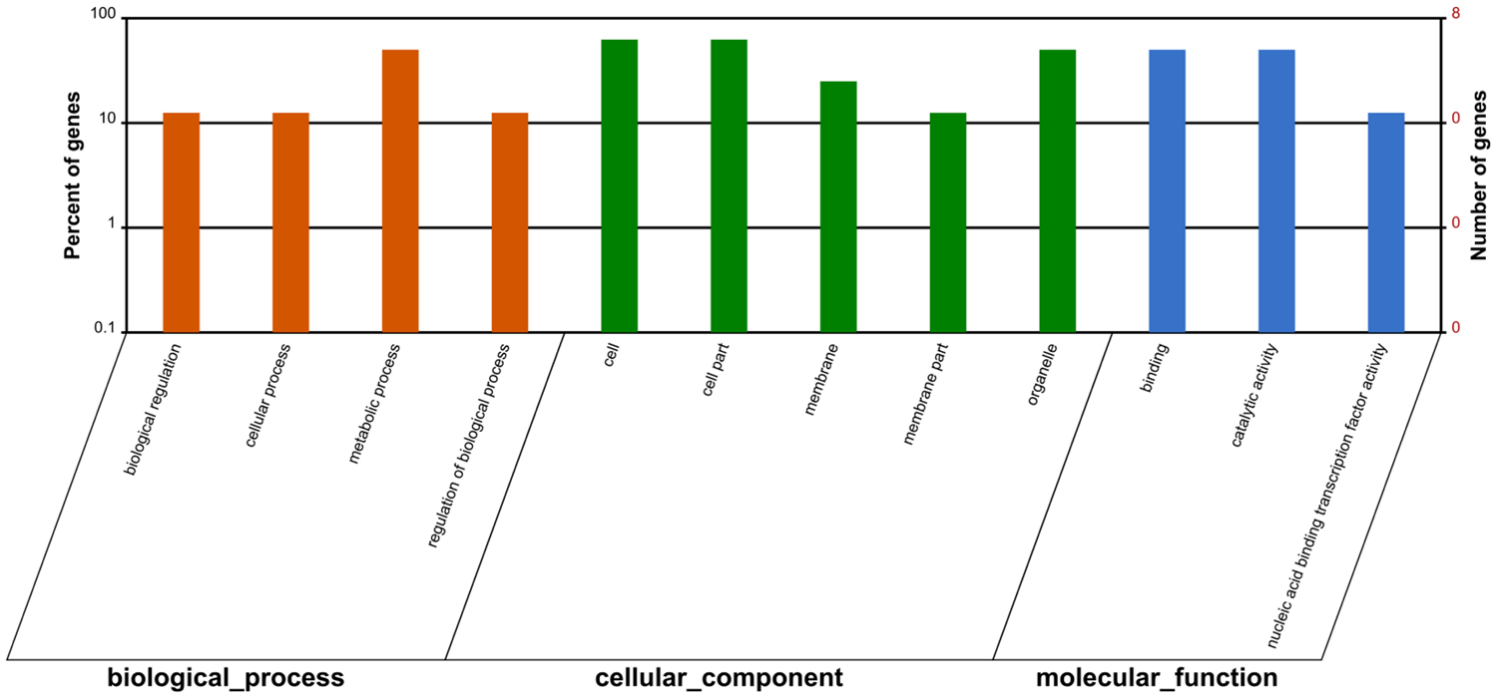
GO classification of target genes for browning-related miRNAs in *Luffa*.

We identified totally 39 browning related target transcripts as putative targets of 11 miRNAs, including three novel miRNAs, two non-conserved, and six conserved (Table S7). The targets were analyzed using Blast2GO about the browning-related miRNAs and it showed that they could be enriched among four biological process categories, five cellular components, and three molecular functions (Fig. 6). The GO analysis on browning-related targets showed that, after comparison with the enriched GO terms in Fig. 4, most enriched GO categories were similar to the putative targets. The biological process and cellular component had three most abundant GO categories consistent with those of all miRNAs targets; while ‘transporter activity’, the third most enriched term among molecular functions, was not found in the browning-related targets. In addition, for the unclassified non-conserved miRNAs, 53 targets were also predicted (Table S8).

For known miRNAs many target genes encoded transcription factors (TFs), such as TOE3 (ethylene-responsive transcription factor), NFYA (nuclear transcription factor), MYB transcription factor, and RAP2–7 (ethylene-responsive transcription factor) (Table 4). Furthermore, some target genes, such as *PHYH* (targeted by miR396), *RGLG* (targeted by miR396), and *UBC24* (targeted by miR399), were identified as connected with browning processes of *Luffa*. *TCB3* (targeted by LcmiRn19) among other sequences was also found to play a major role in membrane transport processes (Table S7), which means that in many biological processes and physiological functions these potential target genes take their roles.

**Table 4:** Identified targets for conserved miRNAs in *Luffa*.

miRNA family	Target sequence	Homologs in *Cucumis sativus*	Target Gene	Target gene annotation
miR169	TR39930_c0_g1_i1	Csa3G782710	*NFYA*	nuclear transcription factor Y subunit A-10-like
miR172	TR41282_c0_g1_i1	Csa2G279250	*TOE3*	AP2-like ethylene-responsive transcription factor TOE3
	TR41282_c0_g2_i1	Csa2G279250	*TOE3*	AP2-like ethylene-responsive transcription factor TOE3
	TR73357_c1_g1_i1	Csa5G138510	*SR34A*	serine/arginine-rich splicing factor SR34A
	TR76908_c0_g1_i1	Csa6G296960	*RAP2–7*	Ethylene-responsive transcription factor RAP2–7
miR396	TR37331_c0_g1_i4	Csa5G289600	*PHYH*	phytanoyl-CoA dioxygenase
	TR37331_c0_g1_i3	Csa5G289600	*PHYH*	phytanoyl-CoA dioxygenase
	TR37331_c0_g1_i2	Csa5G289600	*PHYH*	phytanoyl-CoA dioxygenase
	TR79721_c0_g1_i1	Csa1G002920	*RGLG*	E3 ubiquitin-protein ligase RGLG2 -like protein
miR399	TR71171_c0_g1_i1	Csa6G517350	*UBC24*	ubiquitin-conjugating enzyme E2 24
	TR71171_c0_g2_i1	Csa6G517350	*UBC24*	ubiquitin-conjugating enzyme E2 24

### RT-qPCR validation of miRNAs and their target genes

**Figure 7:**
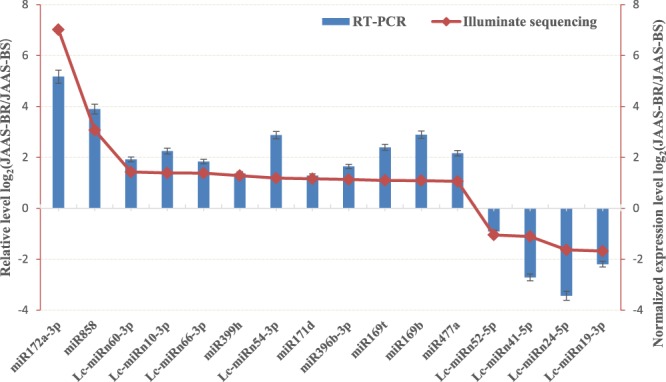
Comparison of relative expression levels of miRNAs between RT-qPCR and Solexa sequencing.

For examination of the Solexa sequencing reads and the dynamic expression patterns of browning-related miRNAs in *Luffa*, we used RT-qPCR to analyze the expression levels of 16 selected miRNAs at 9 DAP, i.e., miR477a, miR169b, miR169t, miR171d, miR399h, miR396b-3p, miR858, miR172a-3p, Lc-miRn10-3p, Lc-miRn19-3p, Lc-miRn24-5p, Lc-miRn41-5p, Lc-miRn52-5p, Lc-miRn54-3p, Lc-miRn60-3p and Lc-miRn66-3p, and further compared them to the results of Solexa sequencing (Fig. 7). The comparison revealed that between NGS sequencing and RT-qPCR most miRNAs shared a similar tendency in expression patterns, which indicates that for miRNAs in *Luffa* the sRNA sequencing data can be used to represent relative expression levels.

**Figure 8:**
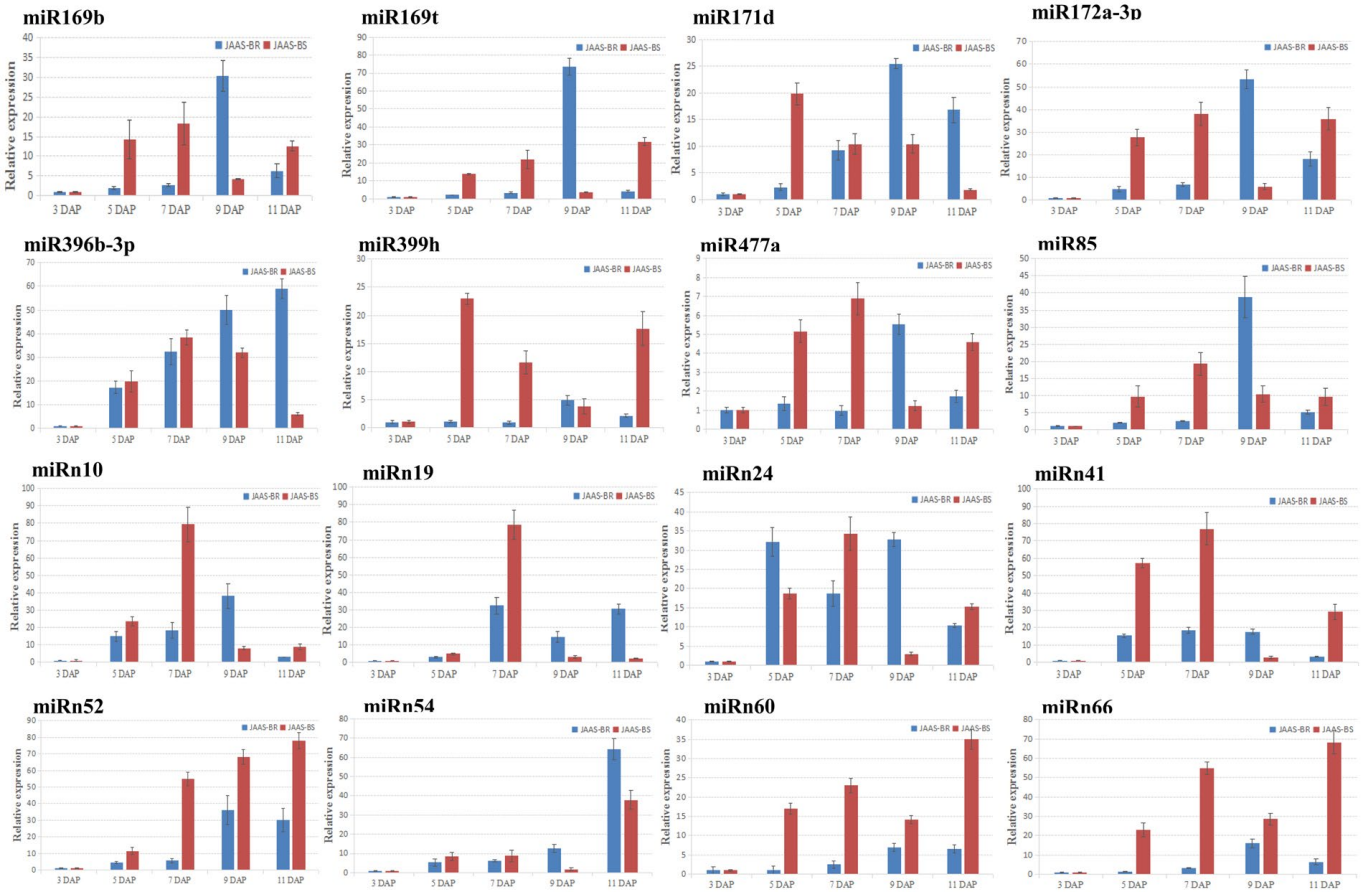
RT-qPCR validation of putative miRNAs at different periods after pollination. The relative expression levels of miRNAs during different development stages, i.e., 3, 5, 7, 9, and 11 days after pollination (DAP). Each bar shows the mean ± SE of triplicate assays.

**Figure 9:**
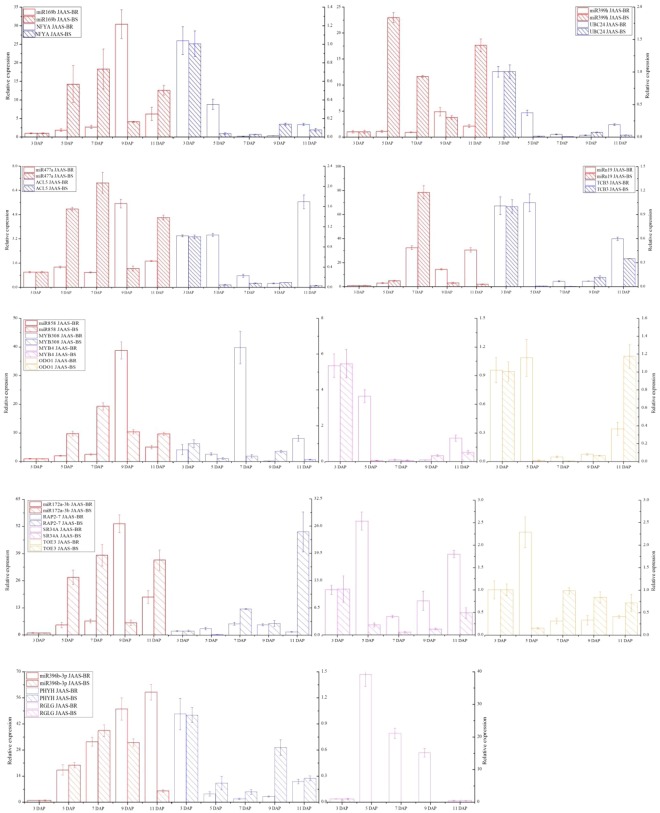
RT-qPCR analysis of several miRNA-targets at different periods after pollination. Each bar shows the mean ± SE of triplicate assays.

We selected 16 miRNAs for RT-qPCR analysis, so as to study the browning-related miRNAs about the expression patterns and their target genes at different days after pollination (Fig. 8). As expected, the expressions of miR169b, miR169t, miR171d, miR172a-3p, miR399h, miR477a, miR858, miRn10, miRn24, miRn52, miRn60, and miRn66 discovered in the JAAS-BR library peaked at 9 DAP and declined at 11 DAP; miR396b-3p and miRn54 exhibited similarly higher expressions at 11 DAP; miRn19 and miRn41 exhibited similarly higher expressions at 7 DAP (Fig. 8). Whereas in the JAAS-BS library, the expression levels of miR169b, miR172a-3p, miR396b-3p, miR477a, miR858, miRn10, miRn19, miRn24, and miRn41 peaked at 7 DAP and declined from 9 DAP to 11 DAP; miR169t, miRn52, miRn54, miRn60, and miRn66 exhibited similarly higher expressions at 11 DAP; in contrast, miR171d and miR399h exhibited similarly higher expression patterns at 5 DAP (Fig. 8). To further confirm the dynamic correlation between the miRNAs and their corresponding targets, the expression patterns of 12 predicted target genes, including *NFYA* (TR39930 targeted by miR169t), *TOE3* (TR41282 targeted by miR172a-3p), *SR34A* (TR73357 targeted by miR172a-3p)*, RAP2-7* (TR76908 targeted by miR172a-3p), *PHYH* (TR37331 targeted by miR396b-3p), *RGLG* (TR79721 targeted by miR396b-3p), *UBC24* (TR71171 targeted by miR399h), *ACL5* (TR32830 targeted by miR477a), *MYB4* (TR9920 targeted by miR858), *MYB308* (TR28685 targeted by miR858), *ODO1* (TR36521 targeted by miR858) and *TCB3* (TR53813 targeted by miRn19), were examined with RT-qPCR at different days after pollination (3, 5, 7, 9 and 11 DAP). As a result, an approximate negative correlation was observed between the expression of majority of browning-responsive miRNAs and their targets. For instance, miR169t, miR399h, miR477a, miRn19 and miR396b-3p exhibited expression tendencies contrary to their corresponding target transcripts during the different DAP (Fig. 9). These results imply that different roles could be played by target genes of browning-related miRNA at different days after pollination. However, most miRNAs will play important roles during the browning process in *Luffa* by negatively regulating their target genes.

## Discussion

*Luffa* browning is regulated by a complex gene network composed of a series of browning-related genes, controlled and coordinated by endogenous and environmental cues^38^. Previous evidence has suggested that in the gene regulatory network in plant growing miRNAs take important roles^11,39,40^. Notably, characteristic regulations by miRNAs and target genes were recently identified for some species^17,23^. However, the few studies on browning regulatory mechanisms with miRNA-directed mainly focuses on the browning of mammalian and human tissues, and there have been no reports identifying or comparatively profiling browning-related miRNAs and targets in plants, including *Luffa*.

### Overview of sRNA sequencing in *Luffa* pulp

A powerful tool is now available by the next-generation sequencing technology, which could be used for identifying comprehensive sets of miRNAs in different varieties and at various stages and for exploring the molecular basis of miRNA-mediated browning regulation. In this study, we successfully identified totally 31 non-conserved miRNAs and 148 conserved miRNAs from JAAS-BR and JAAS-BS libraries by NGS sequencing. Within the distribution of lengths, according to the reported observations in species like *Oryza sativa, Arabidopsis*, and radish^41–43^, the 24-nt length miRNAs dominates, followed by 21-nt class. The conserved miRNA families in the present work mostly include at least one member. Particularly, miR165/166 and miR156/157 each have 14 members, representing the two largest families, and moreover, the conserved miRNA families have an average member number larger than the non-conserved miRNA families, which is in agreement with the previous studies for other species, including strawberry, *Brassica napus*, and radish^32,44,45^. In addition, all novel miRNAs were identified in both libraries, compared to the conserved miRNAs. The result shows that, most novel miRNAs had a lower abundance level than the conserved, as previous reports^45^. The novel miRNAs, expressed at a low level, though, might play roles which are specific to developmental-phases or species in the browning of *Luffa*.

### Characteristics of browning-related miRNAs and their target genes in *Luffa*

Bioinformatics analysis combined with high throughput sequencing help to identify the differentially expressed miRNA and analyze the functions of miRNA, which helps to elucidate potential mechanisms for regulation of biological processes and development paths^13,16,17^. miRNAs take an significant part in cell differentiation, proliferation, function regulation, and apoptosis, as they can regulate the transcription factors involved in the differentiation of brown adipocytes, thus affecting the differentiation and function of brown adipose cells^46^. Many browning-related miRNAs were identified in adipose tissues of animals and humans according to more and more recent studies, which could help to explore miRNAs functions in browning of plants. Recent studies have shown that inhibiting the expression of miR-133 could increase brown cell differentiation, while over-expression of miR133 resulted in decreased brown adipocyte differentiation^47^. miR155 negatively regulates the formation and function of brown adipocytes by inhibiting the differentiation-related regulatory factor C/EBPβ^48^. Gene chip technology showed that the expression of miR-193b-365 was higher in brown adipose tissue, and moreover, the expression level was also significantly upregulated during brown adipose cell differentiation^49^. The above results indicate that miRNA networks are involved in browning regulation. Experimental models and primary adipocyte cultures of mice indicate that miR155, miR133, miR27b, and miR34 negatively regulate the browning, while miR196a, miR26, and miR30 are necessary in this process^50^. Several miRNAs related to browning processes that were detected in human and animals have enriched the knowledge of the regulations of miRNAs in plants, especially in the browning process in *Luffa*. The PPO gene is mainly associated with enzymatic browning, and some identified browning-related miRNAs, including miR168a, were found to be down-regulating PPO genes to avoid injuries and damages caused by browning in plant tissues^19,22^. We identified series of known and novel miRNAs to be browning-related and we used RT-PCR technology to detect their expression levels. The results suggest some previously reported growth and senescence-related miRNAs also showed very different expressions in *Luffa* browning.

Fruit browning is a phenomenon in the growth and development of some plants, during which miRNAs will have differential accumulations across development stages and have differential expressions in fruit tissues as a regulatory role^51^. It was reported that in development of rice and other plants miRNAs had selective expressions and diverse regulatory effects^52,53^. Results have shown that miR858 regulated anthocyanin content in apple and played an crucial role during fruit growth and the high light stress adaptation^10,54^. rsa-miR172 was identified and predicted to target three flowering genes, *AP2*, *TOE2* and *RAP2-7*, which would be down-regulated by rsa-miR172 and regulate flowering time^43^. It was also demonstrated that miR172 expressions produce positive effects on *Arabidopsis* fruit (i.e., silique), while its over expressions will negatively influence growth of apples, leading dramatically reduced fruit sizes^55^. miR169 and miR171 were found to be involved in the anthocyanin biosynthetic pathway^56–58^. miR396 was highly expressed at 50 days after anthesis, while its target genes were down regulated, which indicates the roles of miR396 in the fruit development of hot pepper^17^.

In this study, we found up-regulated miRNAs were more than the down-regulated during each of the different days after pollination. For instance, miR169b, miR169t, miR171d, miR172a-3p, miR396b-3p, miR399h, miR477a, miR858, miRn10, miRn52, miRn54, miRn60, and miRn66 were all up-regulated during the 3–9 DAP in JAAS-BR materials. In addition, the up-regulated miRNAs were also mostly highly expressed in both libraries, indicating that miRNAs take a vital role in the ripening process of the *Luffa* fruit. Some miRNAs, for example miRn19 and miRn41 are firstly up-regulated 3–7 DAP and then down-regulated at 9 DAP. The up- or down-regulations of miRNAs could take a more significant role in network regulation during plant development^43^. The regulatory mechanisms need more investigations to be further clarified for various miRNAs, though, it is possible that the 16 miRNAs with different expressions patterns could take an important role during the process of ripening, aging, and even browning of *Luffa* fruit.

Previous studies have shown that plant miRNAs could regulate corresponding target genes and consequently take parts in almost all development processes. Most miRNAs could encode TFs or key regulatory proteins connected with various plant biological processes^8,44^. We found the Lc-miRNAs in this study target the genes in transcription factor families, such as *TOEs*, *RAPs*, and *MYBs*, most of which were verified to take parts in regulating *Luffa* browning. *MYBs* were verified to take a crucial part during plant growth. Studies have showed that *MYB308* participated in phenylpropane metabolism; overexpression of this transcription factor can affect the biosynthesis of lignin^59^. In addition, *MYB308* has been reported to take part in anthocyanin biosynthesis regulation in apple^10^. In these investigations, two members of the *MYB* gene family, *MYB4* and *MYB308*, were both identified as putative targets of Lc-miR858, indicating that they participated during fruit development and browning in *Luffa*. Additionally, miR172 has been demonstrated to be regulated by the auxin-signaling pathway, as its targeting gene was *AP2*, which was reported to be an important transcription factor affecting fruit development in tomato^33^. In this study, Lc-miR172a-3p was predicted to target three genes, i.e., *RAP2-7*, *SR34A*, and *TOE3*, which involve in transduction of auxin signals and development of fruits by encoding auxin receptor proteins. Moreover, the majority of the miRNAs and their corresponding target genes showed a negative correlation in RT-qPCR, indicating that these miRNAs regulate their corresponding predicted genes^43,45^. Interestingly, in many cases, different targets of a same miRNA were up- or down-regulated while others were expressed inversely, further suggesting that the regulation of the steady state levels of miRNA targets is complex. In addition, the identified browning-related miRNA targeted genes revealed that genes may take part in many biological functions, such as defense responses and signal transductions apart from their participation in browning process. The result suggested that a wide range of biological and developmental processes of *Luffa* are influenced by browning-related miRNAs and their targeted genes.

### miRNA-mediated browning regulatory network in *Luffa* pulp

**Figure 10:**
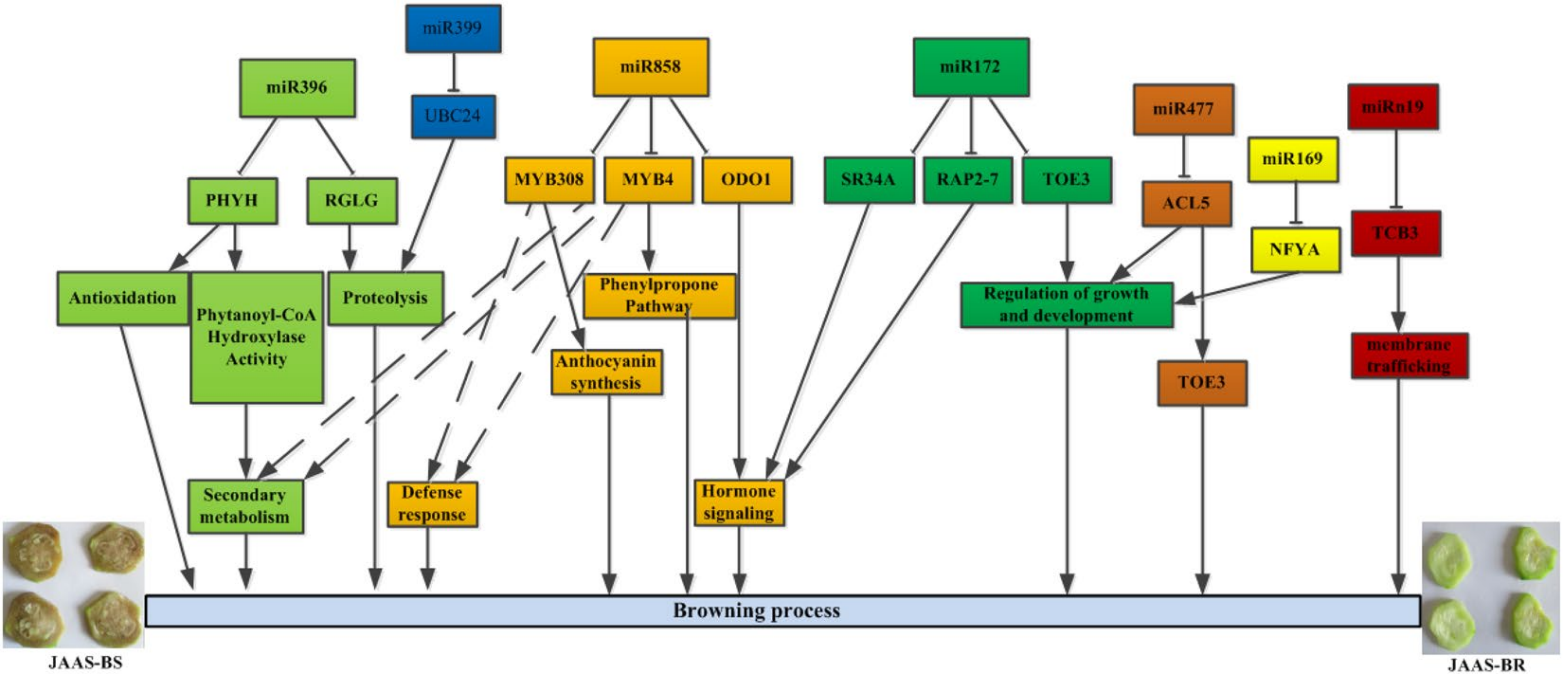
The putative schematic model of the miRNA-mediated browning regulatory network.

Studies showed that miRNAs and their targeted genes participate in browning formation, although crucial roles of miRNAs in fruit development have also been revealed by many functional studies^8^. In this study, we identified the roles of several crucial browning-related genes, i.e., *NFYA*, *RAP2-7*, *RHYH*, and *MYBs*, in the complex genetic networks of browning. We proposed a presumptive schematic model on miRNA-mediated browning regulatory networks after identifying and characterizing *Luffa* miRNAs and the target genes **(**Fig. 10**)**. Of all the predicted targets, some are transcription factors (*NFYA10*, *TOE3*, and *RAP2-7*), which regulate hormone accumulation and plant responses to abiotic stresses and consequently are verified their participation in plant growth and development process^58,60,61^. miR169-targeted *NFYA* (nuclear factor Y subunit A), for example, which will change metabolism of carbohydrates and cell elongation and consequently condition growth of whole plant was regulated extensively in drought and salt stress^62^. RT-qPCR validation and NGS sequencing revealed that Lc-miR169, which was found to target *NFYA10*, was down-expressed at 9 DAP in *Luffa*, in agreement with previous studies (Table 4), indicating that Lc-miR169 and its targets could play important roles in the growth, development, and browning process in *Luffa* (Fig. 10).

TFs (transcription factors) have a significant function in many biological processes as main targeted genes for most miRNAs^45^. For *Luffa* browning, in our work we identified some miRNAs which regulate downstream TFs. ERFs can integrate pathways of ethylene and jasmonic acid in ripening and senescence of fruits according to previous studies^9,28^. Apart from miR172, miR156 and miR396 among other miRNAs were recently predicted in inhibiting *Hevea* transcripts of *29 HbAP2/ERF* genes^63^. miR172 usually targets at AP2-like genes, such as *TOE1* (*TARGET OF EAT1*), *TOE2, TOE3, and AP2*, which mainly participate in regulation of growth and development of plants^61,64^. In our study, Lc-miR172b-3p was identified and predicted to target three genes, *SR34A*, *TOE3* and *RAP2-7*, which were annotated as transcription factors with AP2/ERF domains. miRNA was up-regulated for all different browning materials 3–7 DAP, while expressions continued to increase up to 9 DAP in JAAS-BR materials **(**Fig. 8**)**. At the same time, the target genes *TOE3* and *SR34A* showed a different trend in expression with their corresponding miRNAs, which demonstrated regulation by Lc-miR172b-3p and their associated functioning in *Luffa* development. The exact roles of miR172 in *Luffa* browning, however, need further verifications, since ERF genes may produce an either positive or negative effect on downstream gene expression^65^.

MYBs were proven to extensively involve in regulating metabolism of anthocyanin, a key factor affecting pericarp browning, apart from their functioning in fruit senescence^8,66^. Functions in plant growth and development and browning control are shared by miR858 and the targeted MYB-related protein by regulating several processes, including defense response, hormone signaling, secondary metabolism, anthocyanin synthesis and phenylpropane pathways^8,67^. It was showed in our study that Lc-miR858 targeted at three MYB genes: *MYB308*, *MYB4* and *ODO1*. Additionally browning-related genes, *PHYH* and *RGLG*, were identified as targets of Lc-miR396 in this study, and they participate in redox reactions and protein hydrolysis.

Various factors may affect auxin’s functioning on fruit senescence (delay or acceleration). For example, *Arabidopsis* silique senescence and over-ripening process in tomato were reported to produce auxin responses^68^. Although a body of evidence also supported the functioning of miR393 in mediating auxin signaling and consequently affecting post-harvest senescence of litchi fruit^8,69^, Lc-miR393 was not found to be a differentially expressed gene in this study. This may be attributed to the number of sequencing samples that need to be further studied.

In this study, we first identified browning-related miRNAs and their targets at different days after pollination, and then we used small RNA sequencing technology to comparatively profile them at the transcriptome-wide levels in *Luffa*. We differentially expressed totally eight conserved known miRNAs and eight novel miRNAs, and identified them as browning miRNAs of *Luffa*. We then analyzed the differently regulated miRNAs with RT-qPCR and found them show differential expression patterns. Some browning miRNAs, including miR396, miR858, and miR172, were found to target at several key transcriptional factors and regulating proteins connected with *Luffa* fruit browning, according to GO categorization and functional analysis. The findings of the work are helpful for the understanding of miRNA-mediated regulatory mechanisms of browning in *Luffa*, and facilitate genetic improvement of pulp characteristics in *Luffa*, whilst also advancing the understanding and controlling of browning in crops at molecular level.

## Materials and Methods

### Sample collection

JAAS-BR and JAAS-BS were the genetic materials used, which were derived from near-isogenic lines through multi-generation backcross breeding and selection based on the same original genetic background. Samples of the two types, JAAS-BR and JAAS-BS, free from visual symptoms of any disease or blemishes, and with browning index (BI) of 2.71 and 81.19 respectively, were collected at a stage equivalent to commercial ripeness (9 DAP). Browning appearance of *Luffa* at different DAP and corresponding BI are shown in Fig. S3. *Luffa* were grown in greenhouses after being planted in plastic pots, lighted 16 h (25 °C) on days and 8 h dark (16 °C) on nights. For small RNA sequencing, we collected the fruits from at least five different plants at 9 DAP. To examine temporal expression patterns of miRNAs, pulps were collected at 0, 3, 5, 7, 9 and 11 DAP respectively. We peeled carefully the luffas using stainless steel knives before combining the pulps. Every sample was frozen in liquid nitrogen and stored at −80 °C mediately after being collected from three random individual luffas for later analysis.

### Transcriptome and small RNA sequencing

*Luffa* pulps of same quantity of the three independent biological replicates from near-isogenic lines JAAS-BR and JAAS-BS were mixed for building transcriptome libraries with Illumina TruSeq RNA Sample PrepKit (Illumina, San Diego, CA, USA) according to the use instructions^38^. RNAs were isolated with Trizol reagents (Invitrogen, Waltham, MA, USA) in line with manufacturer agreement.

For the construction of two small RNA libraries, we respectively used the extracted RNA from the pulp samples of the two lines. Briefly, we seperated the 18–30 nt long small RNAs before purifying them with 15% denaturing polyacrylamide gel electrophoresis and then used T4 RNA ligase to ligate them to Solexa adapters at their 5′ and 3′ ends (Illumina). The assembled small RNAs were reversally transcribed to cDNA, followed by PCR amplification. Both small RNAs and the paired-end transcriptome were deep sequenced with a HiSeq. 2000 Solexa sequencer (Illumina) in BGI (Beijing Genomics Institute).

### Bioinformatic analysis of sequencing data

After removing the adapter reads, low quality reads, and contaminated reads beyond the range from 15 nt to 30 nt, the rest small RNAs were aligned with *Luffa* EST and GSS that were kept at NCBI database and *Luffa* reference sequences, including the mRNA transcriptome sequences using the SOAP2 program^70^. Perfectly matched sequences were kept for further analysis. First, we used the non-coding sRNAs from NCBI GenBank databases (http://www.ncbi.nlm.nih.gov/GenBank/) and the Rfam 10.1 databases (http://www.sanger.ac.uk/Software/ac.uk/Softeare/Rfam) by BLASTn search for comparison with the sRNA sequences. Then the sRNA sequences matching small nuclear RNAs (snRNAs), small nucleolar RNAs (snoRNAs), transfer RNAs (tRNAs), ribosomal RNAs (rRNAs) and any sequences with poly(A) tails were filtered out. We then used miRBase 21.0 (http://www.mirbase.org/index.shtml) to compare with the remaining matched sequences for identification of known miRNAs at a maximum of two mismatches. Finally we confirmed all known candidate miRNA precursors using Mfold software about their secondary structures^71^.

### Identification of conserved and novel miRNAs in *Luffa*

For identification of characteristic stem ring structure of conserved miRNA precursors, we used RNAfold program (http://www.tbi.univie.ac.at/~ivo/RNA/ViennaRNA-1.8.1.tar.qz). Unannotated sRNAs were used for identifying novel miRNA after mapping the clean reads to miRBase, Rfam, and NCBI databases. MIREAP (https://sourceforge.net/projects/mireap/) was used for novel miRNA candidate prediction with miRNA sequence lengths of 18–25 bp and sequence lengths of 20–23 bp. In prediction of novel miRNA, we followed the previously reported basic criteria, for example asymmetries between the miRNA and miRNA*, no more than four bulges, maximum miRNA precursor free energy of 18 kcal·mol^−1^, minimum of 16 bp overlapping between the miRNA and miRNA* sequences, and presence of corresponding miRNA* sequences^36^. Additionally, we further screened and verified the predicted novel miRNAs in this study in line with instructions for high confidence miRNA in miRBase21. Mfold software was used for the construction of stem ring structures of pre-miRNAs^71^.

### Differential expressionbrowning related miRNAs

The frequencies of miRNAs from the two libraries were normalized into one million total clean reads for each sample. Differential expression analysis was not carried out in case of expression levels less than one in both libraries because of their low abundance; the expression value was set to 0.01 for further analysis if the abundance of a given miRNA was zero. The fold changes were obtained by: fold change = log_2_ (miRNA normalized read in ‘JAAS-BR’/miRNA normalized read in ‘JAAS-BS’). The *p*-value was obtained in line with reported methods^72,73^. The miRNAs with fold change ≦−1.0 or fold change ≧1.0, (both when *p* ≦ 0.05) were respectively considered to be down- or up- regulated during *Luffa* browning.

### Prediction and annotation of target genes for miRNAs

We used plant small RNA target analysis server (psRNATarget; http://plantgrn.noble.org/psRNATarget/) for predicting the target genes^37^. We used our mRNA transcriptome sequences and the public *Luffa* GSS and EST sequences in the NCBI database as the *Luffa* reference sequences. Blast2GO was performed for GO annotation for a systematic understanding of potential functions of miRNA-targeted genes in *Luffa*. BLASTX search was used to analyze candidate targets with NCBI NR database using default parameters. For a further understanding of the biological functions of the genes, Kobas 2.0 (http://kobas.cbi.pku.edu.cn/home.do/) was usd^74^.

### RT-qPCR validation of miRNAs and potential targets

For validation of the relative expression levels of *Luffa* miRNAs and their targets, and the quality of high-throughput sequencing, RT-qPCR was performed. For validating target genes, we seperated total RNAs from pulps of the near-isogenic lines JAAS-BR and JAAS-BS at different DAP (0, 3, 5, 7, 9 and 11) with Trizol reagent (Invitrogen) and treated them using Superscript III First-Strand Synthesis System (Invitrogen) to reverse transcribe the RNAs into first-strand cDNA. Small RNAs were extracted from the above six pulp samples with a small RNA isolation kit (TaKaRa, Dalian, China). Then, we used a synthesis kit, the One Step PrimeScript miRNA cDNA, to reverse-transcribe the small RNAs into cDNAs. Each PCR reaction was performed with 0.2 μM of primer pairs, 10 μl of 2 × SYBR green PCR reaction mix, and 20 μl containing 2 μl of diluted cDNA, in which 5.8 S rRNAs were used as reference to normalize the expression level. We used Beacon Designer 7.0 (Premier Bio-soft International, Palo Alto, CA USA) to design the specific primers. We listed all the primer sequences of miRNAs and their target genes in Table S1. The RT-qPCR amplification was performed under the conditions described by previous reports^75^. All reactions were performed on MyiQ RT-PCR (BIO-RAD, Hercules, CA, USA) using SYBR Premix Ex TapTM II (TaKaRa) with three biological replicates and technological replicates.

## Conclusion

The application of sRNA sequencing technology combined with bioinformatics analysis provides an unprecedented opportunity to obtain comprehensive understanding of novel and browning-related miRNAs in *Luffa*. A total of 179 known miRNAs and 84 potential novel miRNAs were found to be associated with *Luffa* browning. Totally 16 differentially expressed miRNAs (eight known and eight novel miRNAs) were identified and their 39 target genes were engaged in various biological functions, including plant development, defense response, transcriptional regulation, and signal transduction. Gene ontology categorization and enrichment analysis of the targets corresponding to the differentially expressed miRNAs revealed that a number of miRNA-targeted genes are required for *Luffa* browning. These findings could provide novel insights into miRNA-mediated regulatory mechanisms of browning in *Luffa*, and facilitate genetic improvement of pulp characteristics in *Luffa*.

## Electronic supplementary material

Supplementary Figs

Supplementary Table S1

Supplementary Table S2

Supplementary Table S3

Supplementary Table S4

Supplementary Table S5

Supplementary Table S6

Supplementary Table S7

Supplementary Table S8
