# Supplementary material for: Identification of browning-related microRNAs and their targets reveals complex miRNA-mediated browning regulatory networks in Luffa cylindrica
Source: Sci Rep. 2018 Nov 2;8:16242. doi: 10.1038/s41598-018-33896-9 (PMC6214963; doi:10.1038/s41598-018-33896-9)
Supplement: Supplementary file 1 — Supplementary Figs [file 41598_2018_33896_MOESM1_ESM.pdf]

Identification of browning-related microRNAs and their targets reveals complex miRNA-mediated browning regulatory networks in *Luffa cylindrica*

Yuanyuan Xu, Zhe Liu, Lina Lou, Xiaojun Su*

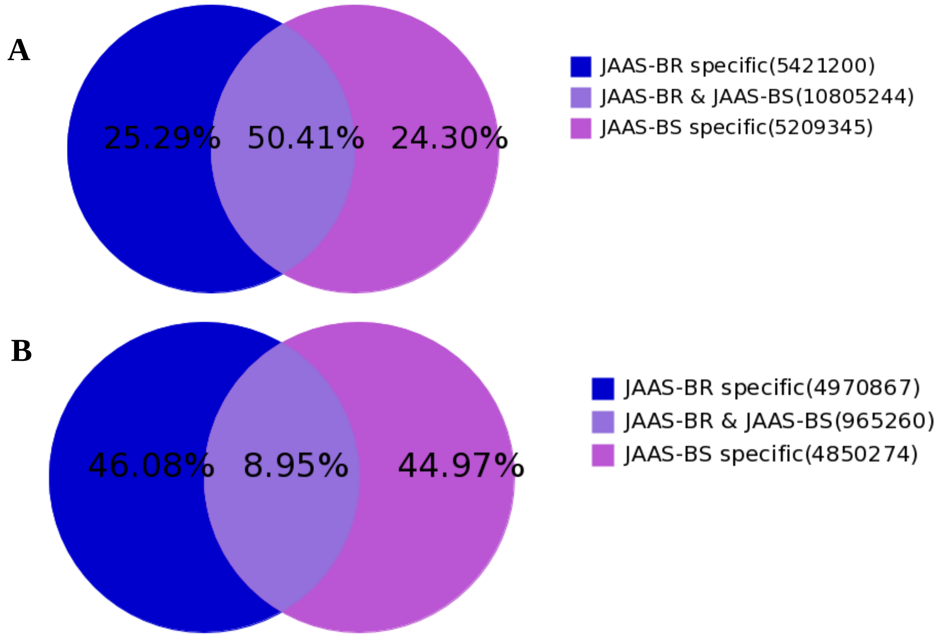

Supplementary Figure S1: Venn chart for total sRNAs (A) and unique sRNAs (B) between JAAS-BR and JAAS-BS libraries.

Supplementary Figure S2: The secondary structures of *L. cylindrica* known and novel miRNA precursors. The 5p mature miRNAs are in red and 3p mature miRNAs in blue. (“.” represent base mismatches, “(” represent base matches).

Conserved miRNAs

miR156b-3p

GTGTTGTGGCAGGTGTGGCAAGGCTATGGGTGACAGAAGAGAGTGAGCACACAGCGG
CCAGACTGCATCTATTCCTTCGGCGGCGGACACGGCCGGGCATGGCCGGCCATGGCAT
GGCATACACGAAAACGAATGATGCTGCTGCTGCTGCGTGCTCACTTCTCTCTCTGTCAC
CTCTCGCACACACGGCTAGCCCGCCAGCCTC

.......(((.((((.(((..(((..((((((((((((((((((((((.(((((((.((...(((((.((((.((((...((.(.((.((((((......)))))).))...).))......)))))..
.)))))))))..)))))))))).)))))))).))))).)))))))))).........))..))).))))))).)))..

****
***GCT
CACTTCTCTCTCTGTCACC******************************

miR156f-3p

TTGTGAGTGAATGAGCTGGGGCAAAAGAAACACACAGAAACTGACAGAAGAGAGTG
AGCACACAAAAGTAAATTGCATATGTTGCATTTGCTTCTCTTGCGTGCTCACTGCTCTTT
CTGTCAGATTCCCGTGCTGATCTCTTTGGCCTGTCCTTGTTCCTCTTTGTCTCAGTCTCT
C

...........((((((((((((((.((((.((((...((((((((((((((((((((.(((((((((((.(((.....)))))))))))...))).))))))))).))).)))))))))....
....((..(.....)..))))))...)))).)).)))))))))).)))..

****
**********************TGCTCACTGCTCTTTCTGTCAGA*************************
****

miR156c

GGTGTCAGAAGAGAGTGAGCACACAGGGTTCTTTGTAGCATGCTTGAGCCTTTCATGC
TAGAAGCTATGTGTGCTCACCCTCTATCTGTCATCCACT

((((.((((((((.(((((((((((.(((...((.(((((((...........))))))).))))).))))))))))).)))).)))).))))....

TGTCAGAAGAGAGTGAGCAC
****

miR156q

TTTGTGGAGGTGACAGAAGAGAGTGAGCACTTGGCTCTTTCCTAGCATGGATCTAAGT
TCACGCCAAAAGCCGTGTGCTCTCTCTCTATCTGTCACCACCTCCTT

.....((.(((((((((((((((.((((((.(((((.(((....((.(((((....))))).)).)))))))).)))))).)))))).))))))))).)).....

**********TGACAGAAGAGAGTGAGCACT***************************************
****

miR156z

TAAACCCAAGTTGGAGCTCTCAGCACTCCAGTGTTGAAGGAAGTGATGGTAAACCCTA
GCTGCTAATTCATGGTTACCTTTGGTTTCACATAAGGAAGCATGTGAAGTCATGGGTCT
GCATGAACTTGGAGATGGGGTTGCCTTGATCTTTTAGTATTGGAGTGAAGGGAGCTCCT
AATTAGGCCTT

.....((.((((((((((((..((((((((((((((((((......(((((.(((((.((.((((((((((...((((.((..(((((((........)))))))..)).))))...)))))).)))
))).))))))))))....))))))))))))))))))..))))))))).)))).))....

****
**AT
TGGAGTGAAGGGAGCT**************

miR156a

AAAGAGGGTTTGGTTGAGAACTGACAGAAGAGAGTGAGTACACGCAGGCAATTGTAT
AGTGGACTATACTCTTGTTTGAGTTATGCGTGTGCTCACTTCTCTTCCGTCAGCCCTTGC
TCTTTTTTTTCTC

(((((((((..((.......(((((.((((((((((((((((((((((((..((((((....))))))..))))).......))))))))))))).))))))).))))).))..)))))))))..
....

**********************TGACAGAAGAGAGTGAGTAC*******************************
****

miR156i-3p

GATATTGACAGAAGAGAGTGAGCACATGCTGCAGTGATTGTATGATAGCATACAATTCT
TGGTGCGTGCTCACTTCTCTTTCTGTCATCTTAT

.....((((((((((((((((((((..((((.((.(((((((((....))))))))))))))..)))))))).)))).)))))))).......

**TGCTCACTT
CTCTTTCTGTCATC****

miR156e-5p

TGGTGGTGATAGAAGAGAGTGAGCACACATGGTGTTTTCTTGCATGATGTTTATATATAT
ATATGCTTGAAACTATGTGTGCTTACTCTCTATCTGTCACCCCCA

.((.((((((((((((((((((((((((((((((...(((..(((((((((......)))).)))))..))))))))))))))))))))))).))))))))).)).

******TGATAGAAGAGAGTGAGCAC**
****

miR156d-3p

TTGACAGAAGATAGAGAGCACTAATGATGATATGCTAATTTCATTCAGCAAAAGCATCT
CACTTCATTTGTGCTCTCTATGCTTCTGTCATCA

.((((((((((((((((((((.((((((((.(((((................))))).)))..))))).))))))))))).)))))))))...

***GCTCTC
TATGCTTCTGTCATCA

miR156e

GTATGGTAGGTGACAGAGGAGAGTGAGCACTCATGGTTCTTTTCTTGCATGAAGTTTCT
ATGCTTGAAGCTCTGTGTGCTTACTCCCTATCTGTCACCCCTCAAGCAT

((.(((..(((((((((((.((((((((((.((.(((....(((..(((((.......)))))..)))))).)).)))))))))))))..)))))))))..))).))..

**********TGACAGAGGAGAGTGAGCAC**
****

miR156a-3p

TTCGTTCCGTGGCTAACTGACAGAAGAGAGTGAGCACACAGCGGGCAGACTGCATCG
ATCGATCTGCATCCGAGACGGCGCACGTACGAATGATGATGCAGCTGCTGCTGCGTGCT
CACTTCTCTCTCTGTCAGTCCTCTAGCTGCTACGGC

.((((...(..(((((((((((((((((((((((((((.((((((.(((.((((((((.(((((.(((..(((...))).))).)).)))....)))))))))))))))).)))))))).)))))
.)))))))))....))))..).)))).

****
************************************GCTCACTTCTCTCTCTGTCAGT*****************
*

miR156e-3p

CCGGCGGGCGCGCGCGGTGACAGAAGAGAGTGAGCACACGGCCGGTGTGACGGC
ACCGGCGCGGATGTGCCGTCGCGGCCGCGTGCTCACTGCTCTCTCTGTCATCCGCTGG
CACACCCTCACG

.(((.(((.(.((..((((((((((((((((((((((((.(((((...((((((((((.........)))))))))))))))).))))))))).)))).))))))).)))).)).).))).).))

****
*******GCTCACTGCTCTCTCTGTCATC******************

miR156k-3p

GGCTTAGGCCCCGAAGAGATTGACAGAAGAGAGCGAGCACCTGGCGCGGCGACCGGC
ATGGAACGCATGCCGTCCCCGCCGCGTGCTCGCTTCTCTTTCTGTCAGCCTCTCCCTCG
TCCTGAG

..((((((...(((((((..((((((((((((((((((((.(((((.((.(((.((((((.....))))))))))))))))).)))))))).)))).))))))))..))))...))).))))))

****
*****GCTCGCTTCTCTTTCTGTCAGC*****************

miR157d-3p

ATGTTGTATGTATAGTGGAGGGTGATAGTGTGGTTGCTGACAGAAGATAGAGAGCACTA
AGGATGATATGCAAAACACACACATATATGTGCTTCTAATTGTATTTCATACTTAACATCA
ATGTTGATGTGAATCGTGAAAAAAAAAAAAGAGAGAAGAGCTAGAGGAGCATCTGCA
TCTCTATTCCTATGTGCTCTCTATGCTTCTGTCATCACCTTTTTTTCTCTATTTCTCTCTAC
ACATTACATATTCACAT

((((.((((((((..((((((((.(((((.(.(((.(.(((((((((((((((((((..((((....((((((......((((....))))........))))))(((((.....((((((....))))
)).....)))))..........(((((..((((((.....))).)))...)))))..))))..))))))))))).))))))))).))))........).))))).))))))))....)))))))..))))

****
****
***********************************GCTCTCTATGCTTCTGTCATC******************
****

miR159b-5p

GGAAGAGCTCCTTGAAGTTCAATGGAGGGTTTAGCAGGGTGAAGTAAAGCTGCTAAG
CTATGGATCCCATAAGCCTTATCAAATTCAATATAATTGATGATAAGGTTTTTTTATGGA
TGCCATATCTCAGGAGCTTTCACTTACCCCTTTAATGGCTTCACTCTTCTTTGGATTGAA
GGGAGCTCTTCATCTCTC

.((((((((((((..((((((((.((((((((..((((((((..(((((((((((.((.((.(((((...((((((((((((((((...(((((...)))))))))))))))).....))))))...))))).
)).)).)))))))).)))))..))))).......)))..))))))).))))))))..))))))))))))).......

****GAGCTCCTTGAAGTTCAATGG***
****
****

miR159b-3p

AAACCCAACTTGGAGTTCCCTGCACTCCAAGTCTGAAAGGATATGATGGTAAACCTCT
ACTGCTAGTTCATGGATACCTCTGACTTCTTAACAACATGCGTTCGAAGTCAAGGGTTT
GCATGCCCTGGGAGATGAGTTTACCTTGATCTTTTGGTATTGGAGTGAAGGGAGCTCCA
GAGGGTATTC

..((((...((((((((((((.((((((((..(..(((((.((....(((((((.((..((.((((..((((.(.(((..((((((((..(((.......))).))))))))..))).).)))))..)))).
))..)))))))))).)).))))))..)..)))))))).))))))))))))).))))....

****

***AT
TGGAGTGAAGGGAGCTCCA**********

miR159c

TACCCAAAACAACATTTAGAGACCATCCATCACAAGAAAAAAGAGTTGCCAAGGGGC
AGCGTGCGACATACCCTGATCTCGTCCTCGGTCGGCGCCGGCGCCGCGGGGCTGCTCC
TGCTGCTGCTGCTGTACATTTGGTTTGAAGGGGGCTCTGTTGGGTGGC
(((((((.........(((((.((.(((.(((((((.....(((((.((..(((((((((..((((.((......)).)))).((((..(((((....)))))))))))))))))...)).))).)).
....))))...))).))))))))))))))))..
****
***TTTGGTTTGAAGGGGGCT
CTG*********

miR159e-5p

CAAAGGGGGTTATGGAGTGGAGCTCCTTGAAGTCCAATTGAGGATCTTACTGGGTGGA
TTGAGCTGCTTAGCTATGGATCCCACAGTTCTACCCATCATTAAGTGCTTTTGTGGTAGT
CTTGTGGCTTCCATATCTGGGGAGCTTCATTTGCCTTTATAGTATTATCCTTCTTTGGATT
GAAGGGAGCTCTACACCCTTCTCCCCTTTTGT
(((((((((....((.((((((((((((..(((((((..((((((..(((((((..(((.(((((.(((((.((((((..((((((..(((((((.............)).)))))..))))))..)
)))))).))))).))))).)))..)))....))))..))))))..)))))))..))))))))))).)).....))))))))).
*******************GAGCTCCTTGAAGTCCAATT**********************************
****
****

miR159b

AAGAGAGCTCCTTTGGGTCCAAACAGAGGGTGTGGATAGGGTGAGATGGAAGCTGCT
GAGCTATGGATTCCACAACCCCACCCTTTTGATTATTGATCCGGTGGGTTGTGGCTCGT
TTTGCATATCTCAGGAGCCTCCGTTCCCTGTTTTCTTCTCTTCCCTCCTTTGGATTGAAG
GGAGCTCTCTT
((((((((((((((.((((((((..(((((.(.((((((((...((((((.(((.(((((.((((.(..(((((((((.(((...(..(...)..)...))))))))))))).......).)))).))
))).))).))))))))))))))....)....))))).))))))))).))))))))))))))
****
****
******TTTGGATTGAAGGGAGCTCTC**

miR159a

GGAGCTCCTTTTGATTCAAAAGCAGGAGGGAGAGAGGGTTTTACACTGCTGCTGGTTC
ATGGATGCCCCTGAGGTGCGCACGATAAGGAAACTGCGCAGCTTAAGGGTTTGCATGG
CCCAGGAGATGTGAATTCCTTCTCCTCTCCTTTCTTGGATTGAAGGGAGCTCC
(((((((((((..((((((.((.(((((((.(((((((.((((((...((.((((..((((.(.((((.((((.((((((......(....))))))).)))).)))).).))))..)))).)).))
))))..))))))))))))))))).))))))..)))))))))))
****
***CTTGGA
TTGAAGGGAGCTCC

miR159d

TGATGTGAGGAGGAGCTCCTTTCGATCCAATTCAGGAGAGGAAGTGGTGGGATGCAGC
TGCCGGTTCATGGATACCTCTGCAGTTCATGCCGGTAGGCCTGCACTTGCATGGGTTTG
CATGACCTGGGAGATGAACCTGCCATTGTGTTCCTCTATTGATTGGATTGAAGGGAGCT

CCGGCTACACCTA

....(((((..((((((((((((((((((((.(((.(((((((((((..((...((.((.((((.(((((.(.((((.((((((.((.(((....))).)).)).)))).)))).).))))).)))
).)).))..))..))))....))))))).)))))))))))))))))))))))..)).)))...

****

****

ATTGGATTGAAGGGAGCTCCG**********

miR159f

GAAGAAGAAGACGAGCTCCCTTCGATCCAATCCAGGAGAGGAAGTGGTAGGATGCAG
CTGCCGGTTCATGGATACCTCTGCAGTGCATGTCGTAGGCTTGCACTTGCATGGGTTTG
CATGACCCGGGAGATGAACCCACCATTGTCTTCCTCTTATGCTTGGATTGAAGGGAGCT
CTACACCTCTCTC

............((((((((((((((((((..((.((((((((((((((.((...((.((.((((.(((((.(.((((.(((((((((.((.....)).))))).)))).)))).).))))).)))).)
).))..)).))))....))))))))).)).)))))))))))))))))............

****

****

CTTGGATTGAAGGGAGCTCTA**********

miR159

GGAGTCTTTCCAGCCCAAAACAGCTTCTTGATCTTCTTGAAAACTTCTGTTTGGACTGA
AGGGAGCTCTAA

(((((((((.(((.(((((.(((.(((..(.....)..)))......))))))))).))).))))).))))..

**TTTGGACTGAAGGGAGCTCTA*

miR159e

CTGATGCTTGGATTTGAAGCGGAGCTCCTATCATTCCAATGAAGGGCCGTTCTGAAGGG
TGGTTCCGCTGCTCGTTCATGGTTCCCACTATCCTATCTCATCATGTATGTGTGTATGTAT
TTTCGAGAGGGAGGAGAGGAGCTAGACTCTCACGGTGGTCGTCTTTGAGATAGGCTTG
TGGTTTGCATGACCGAAGAGCTGCACCGTCCCCTTGCTGGCCGCTCTTTGGATTGAAA
GGAGCTCTTGCATCTGATCCATCCATCC

..((((..((((((.((.(((((((((((.(((.(((((.((..((((((....((((((((((.(.(((..(((.(((((....((((...(((((((((..((((((....))))))......((((
.((.((((((........))))).).....)).))))))))))))))...))))....))))).)))..))).).))))..))))))).))))..)).))))).))).))))))))).))).)).)))))
)..)))).

****

****

***TTTGGATTG
AAAGGAGCTCTT*******************

miR159e-3p

GCAGCTCCTGCAGCATCTGTTCGAGGCTCGCCGCGTTCTGATGATCCGGCAGCGCAAG
TCCGGCGGCGACGGCGGCGGCCGAGGTCGGCGGCGCCGGCTGGCCGTATTGGTTTGA
AGGGAGCTCCAC

(.(((((((.(((.(((.((....((((.((((.((.(((.(((((((((.((....))(((.((....)).)))..)))).))))).))).)))))).))))..)).))))))..))))))).)..

****

**************************ATTGGTTTGAAGGGAGCTCCA*

miR159h-3p

GGTGCTCCCTTCACACCAATAAACGGTCGATCTGATGGGTGGTACAGCTGCTCGTTCAT
GGTTCCCACTGTCCCATCTCATCAGAGAGAGAGAAGATCGTGAGAGAGCGAGAGATG

CTTGAGATGGGCTTGTGGTTTGCATGACCGAGGAGCTGCCTCGTCCCCTTCGTTGACC
GCTGTTTGGAGTGAAGGGAGCTCTGC
((.((((((((((.((((....((((((((..((.((((((..(((((.((((.(((((....((((...(((((((((........((......((((......))))......))))))))))...
))))....))))).)))).)))))..))).)))..)))))))))....)))).)))))))))).))..
****
****
*********************TTTGGAGTGAAGGGAGCTCTG*

miR160a

ATATGCTCGGCTCCCTGTATGCCATTCCCAGAGCCCACCAGTATCACCGGTGACCTCCG
TGAATGGCGTATGAGGAGCCAAGCATATT
(((((((.((((((..((((((((((((.(.(((..((((.(.....).))))..))).).))))))))))))..)))))).))))))).
TGCTCGGCTCCCTGTATGCCA
****

miR160h

ATGTGCCTGGCTCCCTGTATGCCATTTGCAGAGCCCACCGGAGAATCGATGGCCTCCGT
GGATGGCGTATGAGGAGCCATGCATAT
((((((.(((((((..((((((((((..(.(((.(((.((......)).))).))).)..))))))))))..))))))).))))))
TGCCTGGCTCCCTGTATGCCATT
****

miR160b

ATGCTAGATCCGCCTGGCTCCCTGTATGCCAACTGCGAGCCTTGCTAAATATTTGTTTGC
CTTGATTCATAGCTGGCGTTCAGGGAGTCAAGCAGACCTTGCG
..((.((.((.((.((((((((((.((((((.(((.((..(..(.(((((....))))).)..)..)).))).)))))).)))))))))).)).)).)).)).
**********CGCCTGGCTCCCTGTATGCCA**
****

miR160g

GTGTGCCTGGCTCCCTGTATGCCATCTGCAAAGCTCATCAGAAAGTCGGTGGCCTCCG
CAGATGGCATGCGAGGAGCCATGCATGC
((((((.(((((((.((((((((((((((..((..((((........))))..))..)))))))))))))).))))))).))))))
TGCCTGGCTCCCTGTATGCCATC
****

miR162a-5p

GATTCGGGAAATTTTATGTTTCTTTGCCATTTTTTTTGGAGTTTAGTTGGAAAAAGAGTG
AGAGTCGCTGGAGGCAGCGGTTCATCGATCTCTTCCTGGGAACGAATATATAAATATTG
AAAAAGCATGAATAGATCGATAAACCTCTGCATCCAGCGTTTGCCTCTTTATCTTTGTTA
TTGGCTTCAATTTTTTTGGTTTTTGAAAAAAGTGAGGGAAAACAGAGTC
(((((......((((((.((((...((((......((((((((.....(((.((((((.(..((.(((((((.((((.((((.((((((((.(((.((......(((((....)))))........)).))
).)))))))).)))).)))).)))))))))..))))))).)))........))))))))......))))....))))...))))))......)))))
**GGAGGCA
GCGGTTCATCGATC**
****

miR162

TTAACTGGAGGCAGGGTTTTATCCATCCCTTCCTGTTACTGATTTAAGGCTCTGCTCACA
ATTAACTGCAGTTAACAGGCACGGGTTGATAAACCTCTGCATCCAGTTTGG

..(((((((.((((((.((((((.((((...((((((((((..(((((((...))).....))))...))).)))))))...)))).)))))).)))))).)))))))...
****
******TTGATAAACCTCTGCATCCAG*****

miR162a

AGTGAAAGATGCTGGAGGCAGCGGTTGATCGATCTCTTTGTGGGAATTTTTGAAAAAC
AAAATTAAAAGCATGAACAGATCGATAAACCTGTGCATCCAGCGGTCGCCTCTTCA
.((((....(((((((.(((..((((.((((((((.((..((.....(((((.....))))).......))..)).)))))))).))))..))).))))))).)))).......
****
TCGATAAACCTGTGCATCCAG**************

miR162b

TGGGTGATGCCTGGGCGCAGTGGTTTATCGATCTCTTCCCTGCCTTGTGCTGCTCCGAT
CGATGCCCGTGCTGATTCTTGATAATATACAACGCAGGAATCGATCGATAAGCCTCTGCA
TCCAGATCTCACTTT
.((((((...((((..((((.((((((((((((..((((.(((.(((((....((.(((((..((....)))))))...)).....))))).)))))))..)))))))))))).))))..))))...)
))))).
****
************************TCGATAAGCCTCTGCATCCAG**********

miR164b-3p

GATGGAGAAGCAGGGCACGTGCATTACTAGCTCATATATACACTCTCACCACAAATGCG
TGTATATATGCGGAATTTTGTGATATAGATGTGTGTGTGTGTTGAGTGTGATGATATGGAT
GAGTTAGTTCTTCATGTGCCCATCTTCACCATC
(((((.((((..(((((((((....((((((((((.(((((((.((((.((((.((((((.(((((.(((((....)))))))))).))))))...)))).)))).)))....)))).)))))))
)))....)))))))))..)))).)))))
****
***CATGTGCCCATCTTCACCAT
C

miR164f

AGGCGAGGCTTGCGCGGTGGAGAAGAAGGGCACATGCATGCACACACACGCGCATGG
CCGGCCGCTCGGGCGACACGCGCGCCCAGGCCGCGGCGCGGCACACCGGCGGCGAG
CTGGAACGAATGCACGTGCCCTGCTTCTCCACCTCGCGCGCCTCGCCT
(((((((((.((((.((((((((((.(((((((.(((((.(.......(((((.(((((((.(((.(((....).)).))).)).)))))..))))).....(((((.....)))))...).))))).
))))))).))))))))))).)))).)))))))))
*****************TGGAGAAGAAGGGCACATGCA********************************
****
****

miR164e-3p

GTAGGTGAGAACCTCCATGTTGGAGAAGCAGGGCACGTGCAAACCAAAAAACATGAA
ATCTGTTTCATATGCTTTGCACGTGCTCCCCTCCTCCAACATGAGCTCCTCACCATT
...((((((...(((.(((((((((.((..(((((((((((((.((..((((((.(....))))))....)).)))))))))))))..)).)))))))))))...))))))...
**CA
CGTGCTCCCCTCCTCCAAC*****************

miR164b

TGAGCCATGCTGGAGAGGCAGGGCACATGCTAGATTTACCAGCAAGATGGTAAGTTTT
AGCATGTGCTCTTCTTCTCCAACTTGGATTC

.((((((.(.((((((((.((((((((((((((((((((((......))))))).))))))))))))))).)))))))).).))).)))
**********TGGAGAGGCAGGGCACATGCT***
****

miR164a

GTTGAGGAAATTATTGATCATGTTTTTTTGAGTTGTCTGGAATTCGACCGAATTGGAGA
AGCAGGGCACGTGAATCGAATTGGATAGGTTTGGAATGATCTTGGGTGACTCCAATGG
TGGCAGAGAAGTAGCAACCCCACCCGAATCCGTCCTCGTTAGC
(((((.((.....((((((((((..(((((.....(((..(((((....)))))..)))..))))).))))).)))))...(((..(((((((.......(((((....))))).(((.((.........)
).))).....))))))))..))))).)))))
**TGGAGAAGCAGGGCACGT
GAA**
****

miR164c

AGGTTCTTGTTGGAGAAGCAGGGTACGTGCAAAATGCACACCGGTTGGTCGAGCTAAT
TAACAAGCTCTGACGACCATGGTGATCGAATGCACGTGCTCCCCTTCTCCACCATGGCC
TT
(((((..((.((((((((..(((((((((((.......(((((((((.(((((((........))))).))))))))..))))......)))))))))))..)))))))).)).))))).
**********TGGAGAAGCAGGGTACGTGCA**
****

miR164h-5p

GTGGAGAAGCAGGGCACGTGTGAATTCATTCGTTCCATCGACGGTCTGGCGCCGACTC
ACGCATCGCCGGCGCGGCGAGCTGAGCGCGTGACACGGAAAGCAGGTCCGCCGCGCC
GGCGCCCGCGCTGGCCGATCGACCGGCTAACTGTAGCTCCACGAAGCAAAGCCAGGC
CAGCCGGCCGATCGATGCGTGCGTGCATGTGCCCTTCTTCTCCATCG
(((((((((.((((((((((((........(((.((((((((((.((((((((......(((..(((((((((((((..((..((.((.....))....)).))..)))))))))))))...))).((
((((......))))))......(((......))).......)))..)))))))).)))))).).))))))))))))))).)))))))))..
*TGGAGAAGCAGGGCACGTGTG***
****
****

miR166a-5p

AGGGGCTTTCTCTTTTGAGGGGACTGTTGTCTGGCTCGAGGACTCTGGCTCGCTCTATT
CATGTTGGATCTCTTTCGATCTAACAATCGAATTGAACCTTCAGATTTCAGATTTGATTA
GGGTTTTAGCGTCTTCGGACCAGGCTTCATTCCCCCCAATTGTTGCTCCCT
.(((((.....(..(((.(((((.((..((((((..(((((((.((((...((((((.((((((((((((......)))))))))...(((((((....))))..)))......))).)))))).)))).
)))))))..))))))..)).))))).)))..)..)))))..
********************GGACTGTTGTCTGGCTCGAGG*******************************
****
****

miR166e

GGAATGTTGGCTGGCTCGAGGCTTTGACAAAACAGGGTTCTTCTGCAACAAGAACTTC
AAAAAGGCTTCGGACCAGGCTTCATTCCCC
((((((..(.((((..(((((((((.........((((((((.......))))))))....)))))))))..)))).)..))))))..
**GGACCAG
GCTTCATTCCCC

miR166

GGTTTGTCTGAAGGATCTAGGGTTAATTGGGAAGCTTTTTCTTTTGAGGGGAATGTTGT
CTGGCTCGAGGCCACTAACTAGATCTTTGATTTAATCTCAGTTGATCATATATTTGTTTCA
TTTTTCTTCCATAAAATTTAATTATATTGAGATACCCATCATTGATCTTAAGATAATTAAGT
TAGTGACGTCGGACCAGGCTTCATTCCCCCCAATAATTGCTTCTATCTGCTATGATATCA
TCTTCATTTCTTGGAGGTGCTGTGATCAGTTCTCAAACTTTAGTTGATTTTTTCACAATT
TTTCTTTTTGTTTAATTTATTGATCTTGTGTTGTGTATATTTGTATGGTCAACGGAGAGAT
CTTGGTCATTGGCCATGAGCAGATTGGTGTAATGATGAAAGATTTTGACTG

((((((...(((.((((...(((......((((((..((....(((.((((((((..((((((..(((.(.(((((((((((((..((((...((((((((((((..((.((((................
..)))).)))))).))))))))....))))..)))))...........))))))))).).)))..))))))..))))))))).))).))..))))))....)))...(((.((((((((.....)))))))
).))))))..))).))))))).((((..(.(((((((..(((..((..((((((((.......((((((.(((((..((((....))))..)))))..))))))..(((.....))).))))))))..)
)...)))..)))))))..)..))))

****
****
*****************************TCGGACCAGGCTTCATTCCCCC***************
****
****
****

miR166h-3p

TGGGGATGATGGGAATGTTGTTTGGCTCGAGGTAACTGCATGGTCTTAATTTTGTTCAT
CTTTTGAAGCTTTAATTTATTTATGGGTTTCAATCTTTTTTGATCCCTTGAAACAGAAAA
AGCTTTAAAGGTTGGATTTTGAGGCTATCCCTTTATGTGATCTCGGACCAGGCTTCATTC
CCGTCAACCTT

.((((.((((((((((((..((((((..((((((.((.(.((((((((((....(((((.((((((((((((((......((((.(((..((((......)))).))).))))......))))))))))
))).))))).))))))))))).)......)).))))))..))))))..))))))))))).))))

****
****
*TCTCGGACCAGGCTTCATTCC**********

miR166i-5p

GTTGAGGGGAATGTCGTCTGGTTCGAGACCATTCATGCAAGTAGTCTCAGACATGACT
CTTCTGAGTGATTTCGGACCAGGCTTCATTCCCCTCAGC

(((((((((((((..((((((((((((((.((((((.(.(((.((((........)))))))))))))).)))))))))))))..)))))))))))))

*******GGAATGTCGTCTGGTTCGAG***
****

miR166u

GAGGTTGAGAGGAATGTTGTCTGGCTCGAGGTCATGGAGGAGGAGGAGAGTACTGAG
ATGAGTGAAAGTTTCCAATGGAAATTTACCCTCTTAACACAAAAAAAAAATTGATTCTCG
GACCAGGCTTCATTCCCCACACCCA

..((.((.(.(((((((..((((((..((((((((((....((((((....((((......)))).((((((((...))))))))..))))))....))..........))).))))))..)))))))..))))
)).).))).))).

****
*****************************TCTCGGACCAGGCTTCATTC**********

miR166i

TGAGGAGAATGGAAACTGGTTCGAGAACGTTTGTATGGATGAACATAAAAAGAGAGT

CTTTCATCCGAACGATCTCGGACCAGGCTTCATTCTCGCCAGCT
....(((((((((..(((((((((((.((((((.(((((.(((...(......)...)).))))).)))))).)))))))))))..)))))))))).......
**TCG
GACCAGGCTTCATTCTC*******

miR166j

GAAGATGTTATATATATGTGACATTGCCGTGAGGAATGACGTGTGGCCCGAAGCCATAT
CTTGGGTTGCCTTGATGAAGCTCCGGACCAGGCTTCATTCCCCTCGGCAGAGTTGCAA
CAAGTGGGAAAGGG
................((..((.((((((.(.(((((((.((.(((.(((.(((..((((..((....))..))))..))).))).))).)).))))))).).)))))).))..))..............
****
*TCCGGACCAGGCTTCATTCCC******************************

miR166m

TTTCGTTGCAAAGGTGAGGTTGAGAGGAATGTTGTCTGGCTCGAGGTCATGGAGGAGG
AGGAGAGTATTGAGATGAGTGAAAGTTTCCAATGGAAATTTACCCTCTTAACACAAAA
AAAAAAATTGATTCGCGGACCAGGCTTCATTCCCCACACCCAACTTTTTTTTTTATTCCT
.........((((((..(((((.(.((((((..((((((..((.((((((((....((((((....((((......)))).((((((((...))))))))..))))))....))...........)))))..)
)..))))))..))))))))).)))..))))))............
****
**CGGACCAGGCTTCATTCCCC
****

miR166c

ACCAATCGAATCCGGACCAGGCTTCATCCCAGGCATCTGGACCCAATCGACAGCAGCT
CCTTTAGCCTTTGAAAGGAACTCTGTCAAGGTCTCCTCTGCTATAGACAGGAGTCCAG
CGGGGCTAGCATCTCTTGGGGGATGCTGAGGTGTTGGATTATGTTGGT
((((((..((((((((((.(((.((.(((((((.((((((.(((....(((((.((.((((((........)))))).)))))))..((.(((((...(....)..))))).))...))).)))).))
..))))))))).)))..))).)))))))..))))))
***********CCGGACCAGGCTTCATCCCAG***************************************
****
****

miR166n

AAGGGGTGTTTGGAATGAAGTTTGATCCAAGATCCTTGTCTCTCCCGTTAACTTAGTCT
CTGTTACTGTTAGGTTTTCATTACTGAGTGTATTTGCAGCCCCCTTGTCTGATTTTAGCAT
CATGAGAGTAGGAAGTGCTGGTGATCATAGGGTTTGGTTCAAGATCCATTTGACTCTTC
TCTTTATATCTCTCCTGTGTCCTAGCTGGTAATCTGTAGTAGTCTTAATTATCCCTTACTT
CTTGTTAATTTTAAGGTTTGATCTTGCAAGTAATTATATTTGGAAAATATCATAAAATGAT
CTCGGACCAGGCTTCATTCCTTACACCTTG
..(((((((..(((((((((((((.(((.(((((..((....(((.....(((((((...((..(((....)))...))..))))))).(((((((((...(((((.......((((((....((.((((
((....(((((.((.((((((((...(((..((((...............))))...)))..)))))))))))))).((((((....((....))..)))))).)))))))).))))))...)))))).....
..)))))))))).........))).......)).......))))).))).)))))))))))))..))))))).
****
****
****
***TCGGACCAGG
CTTCATTCCTT********

miR166k

GAATGGAGGCTGATCCAAGATCCTCCTCGAGTGCGAAACAAATGCCGTTTTTTTGGTTT
TGTGTTTGCTCTCTTGGGGGGTCTCGGACCAGGCTTCATTCCTC

((((((((.(((.(((.(((((((((..(((.((((((((((.((((......)))))))))).))))).))).)))))))))).))).))).))))))))...

****
***TCGGACCAGGCTTCATTCCT*

miR166c-5p

CGGTTTGTGGGGAACGTTGGCTGGCTCGAGGCATCCTTGCCGACGGCGCGGGTCGAG
GCGTCGGACCAGGCTTCATTCCTTTCAAACCGCC

(((((((..(((((.(..(.((((..(((.((((((.((((...)))).)))).....)).))).)).))))).)..).))))).)))))))..

**********GGAACGTTGGCTGGCTCGAGG**
****

miR166a

GCGGGTGTTTGGAATGAGGTTTGATCCAAGATCCTTCCTTTTTCTCCATTAATTCCTTCA
GTACTGGTCATGATTTAGTTACTTCATAATCGGTTGATCTCGGACCAGGCTTCATTCCTG
ACACCAAGCT

((.((((((.((((((((((((((.(((.(((((.............................((((((.((((..........)))).)))))).))))).))).)))))))))))))).))))))..)).

****
********************TCGGACCAGGCTTCATTCC************

miR167b-3p

ATCTGCACAACTTGTTGCTCAGGTATTTTGAAGACAAGTCCACAAGGGAACAAGTGAA
GCTGCCAGCATGATCTATCTTTGGTTAAGAGATGAATGTGTAAACATATTGCTTAAACCC
AAGCTAGGTCATGCTCTGACAGCCTCACTCCTTCCTGGTTTAGGACCATTCACTGATAA
AGCATTCCACATGCCGAT

(((.(((........((((....((((.((((.....((((....(((((..(((((.(((((.(((((((((((.(((.((((..(((...((((((....)))))).))).)))).))).)))))))
)))).).)))).))))).)))))......)))).))))).)))).))))......))).)))

****
GGTCATGCTCTGACAGCCTCACT
****

miR167a

GGTGTACAGGCATCTGATGAAGCTGCCAGCATGATCTAATTAACTTTCTTTCTCTGTTGA
TTTTATGACAATGGAAAAGAGATGAGTGTCGATTAGATCATGTTCGCAGTTTCACCCAT
TGACTGTCGCACC

(((((((((.((...(.(((((((((.(((((((((((((..((.(((..(((((.....((((((....)))))).))))).))).)).)))))))))))))).))))))))).)...)).))))
.)))))

*****************TGAAGCTGCCAGCATGATCTAA******************************
****

miR167d

TTTTGGGAGGCTGAAGCTGCCAGCATGATCTGTGTATAGATAGTGGTACCAGATCATCC
TGCGGCTTCATTCACTCAAATCACCAACAAT

.((((((.(..((((((((((.((.((((((((.((((........))))))))))))).))))))))))..).))))))...........

***********TGAAGCTGCCAGCATGATCT**
****

miR167

TCAAGCTGCCAGCATGATCTAAAAATCTCTGCATGTGGGGATTATCAGATCATGCTGCA
GTTTAACCT
..(((((((.(((((((((((.(.((((((((....)))))))).).))))))))))))))))))).....
TCAAGCTGCCAGCATGATCTA***

miR167f-3p

CTCTATCAGTTGAAGCTGCCAGCATGATCTTAGCCTTCCTCCTTTGTTGAGGAAAGAAA
CAGATCATGTGGCAGTTTCACCTGTTGT
......(((.(((((((((((.((((((((...(.((((((.......)))))).)....))))))))))))))))))).)))....
***AGATCATGTGGCAG
TTTCACC******

miR167h-5p

CGCTATTAGTTGAAGCTGCCAACATGATCTGAGCTTTCCTTAATTTTCCTATACAGGAAA
GACTAGATCATGTGGCAGTTTCACCTATTGA
......(((.(((((((((((.(((((((((..(((((((.............)))))))..)))))))))))))))))))).)))....
**********TGAAGCTGCCAACATGATCTG***
****

miR167b

CTGCCCAAGGGAACGAGTGAAGCTGACAGCATGATCTATCTCCGAGTGATCAAACAAG
AAACGCTGCGGCAGCCTCACTTCTTCCCGGCG
..(((...(((((.((((((.((((.((((.(((((...........)))))..........))))...)))).)))))).))))))))).
*****************TGAAGCTGACAGCATGATCTA************************************
****

miR167j-3p

ATGAAGCTGCCAGCATGATCTGAAACCATACGTGTTCTTTGATTCCCATCTGAAGAAAG
AGTTGGCTTTCATGGAGAACCGACGGTCAGATCATGTGGCAGTTTCATTT
(((((((((((.((((((((((..((...((.((((((((((...((((((......)))..)))...)))..))))))))..)))))))))))))))))))))))..
****
*********GATCATGTGGCAGTTTCATT*

miR167c

TGCTCCATCATCAGATGAAGCTGCCAGCATGATCTTAATTTCCCTCTGTTTTATCGAGGA
AAAATCAGATCATGTGGCAGTTTCACCTGTTGCTGCTTGCAC
(((..((.((.(((.(((((((((((.((((((((.(.(((.((((.........)))).))).).))))))))))))))))))).))).)).))...))).
**********TCAGATGAAGCTGCCAGCATGA**
****

miR168b

ACCGTCTTGCTCGGATTCGCTTGGTGCAGGTCGAGAACTAATTGTCTAACACATCCACG
AAGTTTTACTTGCTGGTTTGTGAGCAGGGATTGGATCCCGCCTTGCATCAATTGAATCG
GAGCCCTGATCG
...(((..((((.((((((.((((((((((.((.((.((((((.(....((((.(((..(((.....)))..)))..))))....).)))))).)).)).)))))))))).)))))).))))...)))
..
*****************TCGCTTGGTGCAGGTCGAGAA*************************************
****

miR168a

TGTCTCTAATTCGCTTGGTGCAGGTCGGGACTAATTCGCCGGTGGCGGCGGCGGCAAT

CACAACGGCGGTGATTGTTATTAAATGGAGATTAGATGTACGAAGTTATCAACATTTTTT
GTTTTGCGGCGAAATTTGTCCCGCCTTGCATCAACTGAATTGGAGACTGC
.((((((((((((.((((((((((.((((((...(((((((...........(((((((((.......)))))))))...(((..((((...((((........)))).....))))..)))..))))))
)....)))))).)))))))))).))))))))))))...
**********TCGCTTGGTGCAGGTCGGGAC***
****
****

miR168a-3p

CTCTTATTCGCTTGGTGCAGGTCGGGACCTCATTCGCCGGCGCCGGGAATAATGCCGGA
CGAACGACGGCGGTGTTAATTCTACTAAAGCTGTCACCGACGGATAGATGTTTGATTAG
CGGCGAAATTTGGGTCCTGCCTTGCATCAACTGAATTGGAG
((((.(((((.((((((((((.((((((((..(((((((.((((((........)))).))...((((((...((.......))....))))))).....(((((....))))).....)))))))....))
))))))).)))))))))).))))).))))
****
***CCTGCCTTGCATCAACTG
AAT*****

miR169b

CTTGTTTCGTAGCCAAAAATGACTTGCCTGTAGGACATTATCTTCTTAATACTAATTTTG
AGTTTCTTAAATATTGAAGAGACTCTTAATTATTAGTTATATTACAGGCAGTTGTTTTAGG
CTAATTTGACAGGCTCTTTTCGATCATGTTAGATTTTTTCTTCCCTTTTAACTACGAAAAT
ATGAAAAGTTTCTTATCAAGAAAGAGTTAAGATCAAAGAGAGGTACATCTCGAAAAGA
AAAGTATTGTTTGATAGCCAAAAATGACTTGCCTGCTAATTCGATTAGCAAAAGAGATC
TAAATTTATTACTTAGAACTTGAATGAGCTTCATTTTCAGGCAGTCTTTTTGGCTAATCT
AACAAG
((((((...(((((.((((..(((.((((((((...((((......))))..(((((..(((((((((........)))))))))..))))).........))))))))))..)))).))))).....))
))))).(((((((((..((((.....((((((((...((((((((.(.......(((.(((...))).))).....))))))))...)))))))).)))).)))))))))......(((((.((((((
((((((.((((.(((((..(((..((..(((....(((.(((((.........))))).)))......))))))))..))))))))))))))))).))).))))).
*********TAGCCAAAAATGACTTGCCTG**
****
****
****
****

miR169q

GTTTTGTTTGGTGAGCCAAAGATGACTTGCCTTAATATAAGAAACATATAATATAAATGG
GAGAGCTTTAAGTATATATGAAGCTTTCATTGTTTCATATTTAAGTTGGCAAGTTATCTTT
GGCTTACTTCATAAAC
..(((((..((((((((((((((((((((((.....(((.((((((..............((((((((((........))))))))))..)))))).)))......)))))))))))))))))))))))
..))))).
***********TGAGCCAAAGATGACTTGCCT**
****

miR169d

ATTTATCGTGTAGCCAAGGATGAATTGCCGGCGTTTCACGCTGTTGATGGTGCGTGCAT
ATATAAGTTGGCGCCGGCAAGTCATTTCAGGCTACATGTTTGCC
......(((((((((..((((((.(((((((((((.(((((..........))))).............))))))))))).)))))).))))))))).....

**********TAGCCAAGGATGAATTGCCGG**
****

miR169k

CAAAGCATTGCATGGAAAAGAATAGAGTCTAACTGAGCCAAGGATGAATTGCCGGCAT
TGTATATTACCCCTCAAAATGCAATCTATATTTGTCGACCGGCAATTTGTTCTTGGCTATG
TTGGGCTCTCTTCGTTCCATGCAATGCCATG

....(((((((((((((..(((.((((((((((..(((((((((..(((((((((((((((((............)))))))..........).)))))))))..)))))))))..))))))))))
.))).))))))))))))))....

**********************************GAGCCAAGGATGAATTGCCGG****************
****

miR169a

GTAGAGATCGAGCCAAGAATGACTTGTCGGCAGGCTAGCAATTGCTACGTATAGAGCA
ATATGTATGCTAGAATGAGTATTCCGCCAGGTCGTTCTTGGCTCAACTTTG

.(((((...(((((((((((((((((.(((((..((((((((((((.(.....)))))))).....))))))..))......))).)))))))))))))))))..)))))

*********GAGCCAAGAATGACTTGTCGG***
****

miR169t

TGAGCCAAGAATGACTTGCCGGCCGACTTTCGACACCTGTCGTTCTCTGGTCGGCGCG
GTCTTCTTCGGCTCTTG

.((((((((((.(((((((((((((.....((((....)))).....))))))))).))))))))).)))))...

TGAGCCAAGAATGACTTGCCGGC***
*****

miR169n

AAAGGGAGGGAGTGGGGGCTTGAGTCAAGAATGACTTGCCGGTGTTTGCTTCTTGGC
AGGCATTAATTAAGCCGGCTCACCGGCCTTAACCTAGTCCTCCGGCAAGTTGTTCTTGG
CTCACCTTCGCTCTCTTTTCTTTCAT

((((..((((((((((((..((((((((((((..(((((((((.(((((((....))))))).....(((((((((....)))).))))).........)))))))))..))))))))))))))))
))).)))..))))...

********************TGAGTCAAGAATGACTTGCCG******************************
****

miR169v

AGGGAGGAACAAAGCCAAGGATGAATTGCCGGCGATGAAAGTAGGCAGCTTTGTCCA
TGGATCGGCAATTTATTTCTTGGCTATGTTGGGCTCTCT

((((((.((((.((((((((((((((((((((((.((((((((.....)))))...))).).))))))))))).))))))))).))))...))))))

***********AAGCCAAGGATGAATTGCCGG***************************************
****

miR171e

GATATTGGCACGGCTCAATCAAACAACTGCACCACTTTTATTTACATCTCTGTAAACATT
GGTGGTATATATATAAGTCTGAATGAACCGAGCCAACATCAC

(((.((((((.(((.(((.(((.((......((((((..(.((((((....)))))).)..))))))..........)).))).))).))).))))).)))..

****
TGAATGAACCGAGCCAACATC**

miR171

ATCGACGGTTGAAGGGGAGAGTTGTAAAATGAAATCATCAAGGTATTGGCGCGCCTCA

ATTTAAAGACGTGGTTAAATGGGCATGATTAGCCATGTATTTTCATTGAGCCGCGTCAAT
ATCTCCTTAATTATTTTGTAACTCTCTCCTCTATATCCTCGCCTTCGGTATGCAGCTGCTC
CTCGATACATATGAGGATTCAGAAACAGACAAAGGCGGTAGAAGTAATCTTCATCAATA
TTATTGAAGCAGGAAACATAACGGCAAGTTTTAAGACCCGTTTGGGGCATGTGGGGTC
TCATTTTGATGTTAATGAAGTGAAAACTTGTATTTTCCCTCAAACATTCACTCACTCCAG
GCCGGCAGGAACAAC
.((((..(((((.((((((((((((((((((........(((((((((((((.((((((..(((.((((((((((..........)))))))))).)))..)))))).)))))))))))))).....
.))))))))))))))))))).........(((((((.(...((...(((.((((((.......))))))..)))...))..).)))))))..((((....)))).)))))....)))).((.(....)....
((((..(((....)))..((((((((.(((..((...(((((((.........)))))))...))..)))....))))))))..............))))))).........
****
************************TTGAGCCGCGTCAATATCTCC**************************
****
****
****

miR171-5p
GCGGGTTGATGGCCAGGAACTCCGACAAGAGGAGGATGCTGAAACGGTCACTATGAT
GTTGGCTCGACTCACTCAGACCACGCCGGAGGGAGCCATCTGCGGCGGCGGTTCTGAT
TGAGCCGTGCCAATATCTTAGTGCTCTTTCATGCTCGTCCCTTGTCGCCATCACCCAC
..((((.((((((..((....))((((((.(((.((.(((((((.((.(((((.(((((((((.((.((((.(((((((.(((((.(((......))).)))))..)).))))).)))).)).)))
)))))).))))))).))))).)))).)))))))))))))))))))..
***TGTTGGCTCGACTCACT
CAGA**
****

miR171a
TGAATTCCCCTCCGCTTTTTGATGTTGGCTTGTCTCAATCAAATCAAAGTTCTTGAAATT
TGAGTTCTTTAGTCTGATTGAGTCGTGCCAATATCATATTAAGCGATAAAAGTC
............(((((..((((((((((.((.((((((((.((.((((..((..(...)..))..)))).)).)))))))).)).))))))))))....))))).........
***TGA
TTGAGTCGTGCCAATATC*******************

miR171c
ATTGAGATATTGGCTTGGTTCACTCAGAAGAAACAAAAAATAACACTACTTGTGAATGT
TTTGATTTTGATTTTGATTTTGATTGAGCCGTGCCAATATTTCAAA
.(((((((((((((.(((((((.((((((((((((((((.....(((.....)))....))))).))))........)))))).))))))).))))))))))))).
****
TGATTGAGCCGTGCCAATATT*****

miR171c-5p
GTGGGAACGGGATATTGGTGCGGTTCAATCAGAAAGCTTGTGCTCCGAAGGCGAGGG
GCTCCACTCTTTGATTGAGCCGTGCCAATATCACGTCGCCTT
..((..(((.((((((((..((((((((((((((.((...(.((((((........)))))).)..)).))))))))))))))..)))))))).)))..))..
*********GGATATTGGTGCGGTTCAATC***
****

miR171d-5p
TTGTAGCTATGATGTTGGCCCGGCTCACTCAGATGGATCATCGGTGCAGAAGAGTGCAT
GAATCTGATGCAGTCTCAGTGTAGTATGCTCCATGCTGGAACTTCTGATTGAGCCGTGC

CAATATCTCAGCACCAT

.....(((..(((((((((.(((((((.(((((((((.(((.(.((((..(((.(((((.......))))).)))...)))).)))).))))..........))))).))))))).)))))))))..))

).....

************TGTTGGCCCGGCTCACTCAGA*************************************

****

miR171d

ATGGAAGAAGCGATGTTGGTGAGGTTCAATCCGAAGACGAATTTATGCTTATTTTCGTA

AAGAACGATCTCAGATTGAGCCGCGCCAATATCACTTCTTATTTTCATTCCGT

((((((((((.((((((((((.(((((((((.((.((((..((((((........))))))...)).)))).))))))))).)))))))))).))))).........)))))

***TTG

AGCCGCGCCAATATCAC******************

miR172f

GCTAATGCAGCATCATCAAGATTCTCAATCAAAATCATCTTCCAAACAAATCAATCCCA

CAAAAACAAAATTTTAAAAAACAAATAATTAGGGTTTCCTTTCTTTTTTCTTTATTTATT

TCTTATTTTGTTGTGAATCTTGATGATGCCGCACAAGCAAATTA

(((..(((.((((((((((((((((.(((.((((((.......................((((......))))).......((((((.(((((...............))))).))))))...))))))))).)

)))))))))))))).))))..)))......

****

**TGAATCTTGATGATGCCGC

AC**********

miR172a

GTTTGCAGATGCGGCATTATCAAGATTCACAAACTTAGTACAGGGTTTCATTGGAGAGC

CAAAGGGTGTGGTTTAGTGGTACCCCTCCACAAAATCACCTCCTTTTACTTCAAAGTTT

TCCTTTCAAATATGGGAATCTTGATGATGCTGCATCAGCAAACA

((((((.((((((((((((((((((((((.((..........((((...(.((((((....((((((.(((((((.((((.......)))).))))))).))))))..)))))).)...)))).......

.)).))))))))))))))))))))))).)))))).

**********GCGGCATTATCAAGATTCACA**

****

****

miR172b

GTTTGCTGCTGCAGCATTATCAAGATTCACAAGAGGCGTTTTAGGGTCTTTTTTGATTG

ATTGATCAATCAATAAGCCCTTTTATGATTGAGAATCTTGATGATGCTGCAGTGGCAATA

..((((..((((((((((((((((((((.(((....(((...((((.(((..((((((((....)))))))).)))))))...))).))).)))))))))))))))))))))..))))..

**********GCAGCATTATCAAGATTCACA**

****

miR172j

TGATTGCCGCTGCAGCATCATCAAGATTCCCAATACCCCCGCCCATGAATGATTTGGGC

AAAAGTGAAAAAAAAAAAGCTTATAAAGATAAGCTCTTTTATTTTAACTTTGCCTTTGT

GGGTTTGTGAATCTTGATGATGCTCCAATAGCAAGCAGTGGCTGTTA

((((.(((((((((((((((((((((((((.(((.((((..(((((.........))))).(((((.((((.((((.((((((((....))))))).)))).)))).))))).........))))))).)

)))))))))))))))..........)))))))).))))

***********GCAGCATCATCAAGATTCCCA***************************************

****

****

miR172d-5p

GTGCAGCACCATCAAGATTCACATCCCCAGCTCGATCTGTGCATGATGAGATGAGAATC
TTGATGATGCTGCAT

((((((((.((((((((((((.(((((.((((.(.....).)).))..).)))).)))))))))))).))))))))

CAGCACCATCAAGATTCACA
**

miR172a-5p

AGGCGGCACGCACAGTCGGCGCTTGCAGGTGCAGCACCACCAAGATTCACATCGGGT
TCTCCAGTCGTAATTAATTAACGAGGAGTACAACCATGATGAGAATCTTGATGATGCTG
CATCGGCAACCGCTCGACTACTCATCATATCGTCAAT

.(((((.......((((((((.((((.(((((((((.((.((((((((.(((((((((((((..((((.........))))))))...)))).))))).)))))))).)).))))))))).)))).
))).))))).........)))))...

******************************GCAGCACCACCAAGATTCACA********************
****
***

miR172b-5p

TAGCTCATCTGTCGTTGTTTGTAGGTGCAGCACCATCAAGATTCACATGAAAATTGATA
AATCTCCTAAATTAGGGTTTTGATATGTATATGAGAATCTTGATGATGCTGCATCAACAAT
CGACGGCTACAAAATACCTAAAGCTG

(((((.....((((((((.((((.(((((((((.(((((((((((.((((...((...((((.(.((((...))))).))))..))...)))).))))))))))).))))))))).)))).))))))
).............)))))

**************************GCAGCACCATCAAGATTCACA************************
****

miR172a-3p

GTTTGCGGGCGCGGCACCATCAAGATTCACATCCATCCCATCCAGGCATCCTTCGGTTC
GTTTCCAAAACGAAACGATCGACAACGCGTTGGATACAATATAGATGTGCATGAGAATC
TTGATGATGCTGCGTCCGCACACAA

((.(((((((((((((.(((((((((((.((((((((..((((((((.....(((((.((((((......))))))))))).....)).))))))........)))).).))).)))))))))))).))
))))))))))).))..

****
*********************************AGAATCTTGATGATGCTGCGT**********

miR172a-3p

TAAGTACTTTCGCTTGCAGAGAGAAATCACAGTGGTCAAAAAAGTTGTAGTTTTCTTA
AAGTCTCTTTCCTCTGTGATTCTCTGTGTAAGCGAAAGAGCTTG

(((((.(((((((((((((.((((((.((((((.((.........(((.((....)))))).........)).))))))))))))).))))))))))))).)))))

**TGA
TTCTCTGTGTAAGCGAAA*******

miR319a-5p

ATTATTAGAAATAGATGAAGAGAGCTTCCTTCAGTCCACTCATGGAAGGGTAAGGGGTT
TGAATTACCTGCTGACTCATTGATTCAAACACAATAGACAATTATGGGGTTATGCTATTG
TGAATGTGTGAATGATGCAGGAGGTGAATTTCTTCCTTTTCTTCTTTGCTTGGACTGAA
GGGAGCTCCCTTTTTCTATTTATAAATTA

....(((.(((((((.((((.(((((((((((((((((..((.(((((((.((((((...((.((((((.(((..(((((.((.((..((((((((..((((....))))...))))))))..)).)
).))))))..))).)))))).))..))))))))))))).))..))))))))))))))))).)))).))))))).)))....

********************AGAGCTTCCTTCAGTCCACTC******************************
****
****

miR319f

AATTAAGGTGGTCTGCTTCTGATCCAACGATGCGGGAGCTGCATTTAGTCGTGACTGTC
ACGTCTTGGACTGAAGGGGCCTCTTCATTTCCCATA
.....(((.(((((.((((.(.(((((.((((.((.((..((........))..)).)).)))))))))).))))))))).))).........
***TTGGACTGAA
GGGGCCTCTT***********

miR319p

GAAATGGGAGTTCCTTGCAGCCCAAAGCTTCAAGGATGAAATTCTTCCTGCGGTTTTG
GACTGAAGGGAGCTCCTACTT
....((((((((((((.(((.((((((((...((((.(.....).))))..)))))))).))).))))))))))))...
***TTTTGGACTGAAGGGAGCT
CC*****

miR319q

GAGAGTGAAGGAGTTTCCCTCAGCCCATTCATGGATATAATGAAAGATTGGGTTGCTGA
ATTAACTGATTCATTCATACAATAGTATTCAATTAGGGTAATATTGTGTGAATGAAGAGA
GTATAGTATCTATATTGGAACCCCCTCTTTCTCTGTGCTTGGACTGAAGGGAGCTCCTTC
TTTTT
(((((.((((((((((((.((((.(((..((((((......((((((..(((((.(............((((((((((((((.(((((.....))))).))))))))))))))...((((((((...
)))))))).))))))..))))))))))))..))).)))).))))))))))))))))
****
****
TGGACTGAAGGGAGCTCCTTC*****

miR319a

GTGGAGCTCCGTTTCGGTCCAATAGTGGCTGCGACGGAAGGTGGTCCCGCTGCCGAAT
CACACGTCCGGGTTGCTTATCGGGGCAGGGCCCCGATACGGTATCCGAACGTTTGTCCC
GGGAACTGGTCGACCTTCCGCCCGGCGTCTCTTGGACTGAAGGGAGCTCCAC
((((((((((.((((((((((((.((..((((.(.((((((((.(.((.(.(.(((...((.((((.(((((.((((((((((((...)))))))))).)))))))).)))).))...))).).).).))
.).))))))))).)))))..)).))))))))))))))))))))
****
**CTTGGACT
GAAGGGAGCTCC**

miR319c

TAACCTCACTGGCTGTGGGAGCTTCCTTCGGTTCAATAGTGGCTGATATGAGGTTGCAC
TGCTGCCGACTCAAACTTCCGGCTTCCCTCTCTTAGAATGGCAGGGAATCCGAATGTCT
GATGCGGGAGCTGAGCGGTCTTCAACTCAGCTTCTCTTGGACTGAAGGGAGCTCCCAT
GTCTTTGTGGTTA
(((((.((..(((.((((((((((((((((((((((.((.((((((..(((((((((.(.(((.(((..(((.((...(((.((((((.((.......)).)))))).)))...)).)))..))).)
)).).)))).))))))..))))))).)).)))))))))))))))))))))))))..)).)))))
****
**CTT
GGACTGAAGGGAGCTCCC***************

miR319

AGCTCCTTTCAGCCCAATACGGAGGACTGAGATGTGGGTAGAGCTGCCATCTCATGCAT
AAGGCTATGCTTAATATTTCTCAAACAGAAACTTCCAGTCATCAAATTTGAGCTGCCAT
CTTCAAGGACTTAATGTTAAGCTCAACCAAATGCATGGAGTGGGAGCAACTCCTTCCC
CATACTTTGCCCGCCCATTGGACTGAAGGGAGCTCC
(((((((((((.(((((..((.((.(.(((.((((((..(((((.((((..(((((((..((....((((((((((..((.....(((.....((((((.......))).)))).....)))...))...
))))))))))....))..)))))))..)))).)))...))....)))))))).).)).)).))))).))))))))))))..
****
****
**********************************ATTGGACTGAAGGGAGCTCC

miR319e

GGAGGGGCTGAGGTGCTCCTTTTAGTCCAATACGGAGGGCTGAGAAGCGGCTAGAGC
TGCCATCTCATGCATTTAGGCAATGCTTAACATTTGACGAAGAGAGGTTAAGCTTGGCC
ACTTGCATGGTGTGGGAGCAACTCCTTCCGCATGCTTTGCTCGCCCATTGGACTGAAG
GGAGCTCCTGGTTTTACC
((..(((((.(((.((((((((((((((((..((.((((.(((..((((...(((((.((((..((((((....(((...(((((((.(((.....)))...)))))))...)))...))))))..)
))).))))...))...))))...))).)))).)).))))))))))))))))).))))))))..))
****
****
*****TTGGACTGAAGGGAGCTCCT*********

miR319i

GTGCAGAAATGGAGGTTCCTTCCAGCCTCAAGCATCTTTAAGATGGTGGCGATGTCTAT
TTTGGGCTGAAGGGAGCTCCCAATTCTTCT
..(.((((.(((..(((((((.(((((.((((((((.(((......))).)))))).....)))))))).))))))))..))).)))).).
**TTGGGCTGAAGGG
AGCTCCC*********

miR319c-5p

GGAATGGTCCAAGAGAGCTTCCTTCAGCCCACTCACGGAGGAGAAATGGGGTTGAATT
AGCTGCCGACTCATTCATCCAACCACTCAGTAGAAAGGATAGGTTTTGTGCTACTGTGA
TTGAGTGAATGATGCGGGAGATAGTTTTCTATCCCTCTCTTTCCTTGCTTGGACTGAAG
GGAGCTCCTTTTCCCCATT
..(((((...(((.(((((((((((((.(((..((.((((((((...(((((.((((((.((.(((..(((((((..(((.(((..(((((...(((.....)))...)))))))).)))..)))))
))..))).)).))))))...)))))))))).)))).))..))).))))))))))))))))....)))))
*************AGAGCTTCCTTCAGCCCACTC*************************************
****
****

miR390b-3p

AGTAGAAAAATAGCTATAAAGCTCAGGAGGGATAGCGCCATGAATCACCAATACTGTA
GGTTTTGTATATACATGCATGTAGATCTGTTGGCGCTATCCATCCTGAGTTCCATAGCTTC
TTCTTGCT
((((((....((((((..(((((((((.((((((((((((.....((....(((((((.((.........)).)))).)))....)).))))))))))).)))))))))..))))))..))).))))
****
************CGCTATCCATCCTGAGTTCCA***************

miR390-3p

GTAGAGAAGAATCTGTAAAGCTCAGGAGGGATAGCGCCATCATGATCACACACATCGA
TATTTTTGGCGCTGTCCATCCTGAGTTTCATTGGCTCTTCTTACTAC
((((((((((..(.((.((((((((((.(((((((((((.....(((.........))).....))))))))))).)))))))))).)).)..))))))..))))
***CGCTGTCCA
TCCTGAGTTTCA*****************

miR390a-3p

AGAATCTGTAAAGCTCAGGAGGGATAGCGCCATGGATGATCTTCTCTTCACTCTTGATC
TTCTCTTGCGCTATCCATCCTGAGTTTCATGGCTTCTATCTTCACA
((((.((((.((((((((((.(((((((((...(((.((((..............)))).)))...))))))))).)))))))))).)))).)))).........
***CGCTATCCA
TCCTGAGTTTC******************

miR390b-5p

AGAATCTGTAAAGCTCAGGAGGGATAGCACCGTGATACTGATGATTATATGTTCATAGTT
GTATACGTACTTGGCGCTATCTATCTTGAGCTTCATGGCTTCT
((((.((((.((((((((((.(((((((.(((.(.(((..(..(((((......)))))..)....)))).))).))))))).)))))))))).)))).))))
**********AAGCTCAGGAGGGATAGCACC**
****

miR390d-3p

AGAATCTGTTAAGCTCAGGAGGGATAGCGCCCTAAGGATAACCATGGGCTCTTTTTATT
TGGTTTTTGACTATCAGTGGCGCTATCCATCCTGAGTTTTACTGGTT
..((((.((.((((((((((.((((((((((((...((((..((..((((..........))))..))..)))))).)))))))))).)))))))))).)).))))
****
CGCTATCCATCCTGAGTTTTA******

miR393a-3p

AGAGGAAGGATCCAAAGGGATCGCATTGATCCTAATTAAGGTGAATTCTCCCCATATTT
TCTTTATAATTGGCAAATAAATCACAAAAATTTGCTTGGTTTTGGATCATGCTATCTCTTT
GGATTCATCCTTC
.(((((.((((((((((((((.((((.(((((.(((((.((.((....)).)).................(((((((...........))))))))))))..))))))))).))))))))))))))).))
))).
****
*************************ATCATGCTATCTCTTTGGATT********

miR393b-3p

AGAGAAAGGATCCAAAGGGATCGCATTGATCCTAATTAAGCTGATTTATTCCCCAATAA
TTGTTTTTTTTTTTCCTTCTCAATCGAAAGATGGAAGAAAAACAAATTCCAAACATTTTG
CTTACTTTTCCGGATCATGCGATCTCTTTGGATTCATTCTTT
((((((.(((((((((((((((((((.(((((.((.(((((.((.....))........(((((((((..((((......(((....))))))))))))))))..............))))).))....)
))))))))))))))))))))))).))))))
****
**ATCATGCGATCTCTTTGGATT
****

miR394-3p

CGAGAGCTCTTTGGCATTCTGTCCACCTCCTTGTGAATCTTGCAGGGGAGATCTCACGA
TCTGTCGAGCTTGCGGGTTGCTTGGAGGTGGGCATACTGCCAATGGAGCTGTGTAG
..(.((((((((((((.(.(((((((((((..(..(..(((((((((.(((((....))))).)....))))))))..)..))))))))))).).))))))).))))))).)....

****
*****AGGTGGGCATACTGCCAATGG**********

miR394a

CTTACAGTCATCTTTGGCATTCTGTCCACCTCCTTCTATACATATATGCATGTGTATATATA
TATGCGTTTCGTGTGAAAGAAGGAGGTGGGTATACTGCCAATAGAGATCTGTTAG
((.((((((.(((((((((.(.(..(((((((((((.(((((..((((((((((....))))))))))...)))))..)))))))))))..).).)))))).))))).)))).))
*************TTGGCATTCTGTCCACCTCC**
****

miR394a-3p

TCATGTGGGTTTTGCAAAGGGTTTCTTACAGAGTTTTTTGGCATTCTGTCCACCTCCATC
TGTAGAAACTACAAGTTGTTCTACTTTCTGGAGGTGGGCATACTGCCAACTGAGCTCTG
TTGGTCTCTCTTTGTAAAACCCTCGTGA
(((((.((((((((((((((((...((.(((((((((.((((((.(.((((((((((((...((((((.(........)))))))....))))))))))))).).))))))..))))))))).))...
))))))))))))))).)))))
****
**************************************CTGTTGGTCTCTCTTTGTAA***********

miR395c-3p

ATGACAGAGCTACAAGAAGAAAGTCAAATGTCTTCATGAGTTCCCTTTAACGCTTCATT
GTTAATATTCAAACTACATTTATTTCCATAAATGTTTGATCGATTTGTATATAACACTGAA
GTGTTTGGGGGGACTTTTGGTGTCATTCTGGCGTAGTTACGATTAT
((((..(((((((.........((((((((.(.(((.(((((((((..(((((((((.((((((((((((((...((((((((....)))))))))))))).......)))).))))).)))))))))..
)))))))))).))).).))))).))))))))))).)..))))
****
*************************************CTGAAGTGTTTGGGGGGACTT**************
****

miR395d-5p

ACAAAATATAATTCGACTAGATGTCCTCTAGAGTTCTCCCGAACACTTCATTGGAAATTT
GTTATTCGGTAAACTACCAATTAATTCCACTGAAGTGTTTGGGGGAACTCCGGATGTCA
TTTGGTGATGTCAAGCCATGT aly-MIR395d 140
...........((.((((((((.(.((..((((((((((((((((((((.((((.....((((...((((...))))...)))))))).)))))))))))))))))))))..)).).))))))))))).
....
*********************************GTTCTCCCGAACACTTCATTG*******************
****

miR395b-3p

CTTACTGCGGGTTCCCTGCAAACACTTCACGAAGCATTATTTCAAACGCCATTGCGAAG
TGTTTGGAGGAACTCTTGGTGAAGTCAAGCTCCAAGAACTAAGAAGGTGATCAATACG
AAGCATGTTTGGTCTTACCGTGGGTTCCCTACGAGCACTCCACGAGGCATTATTCCAAA
TACCACTGTGAAGTGTTTGGGGGAACTCTTGGTGATGC
.((((((.(((((((((.((((((((((((((((.....))))....(((.(((.(.((((((((..(((((((.((((((..((((((.......(((.....)))..((.....)).....))))))
..)))))).)))))))...)))))))).).))))))................)))))))))))))))))))).)))))))...
****
****
*****************************AAGTGTTTGGGGGAACTC**********

miR395e-5p

CTTACCGTGGGTTCCTTACAAGCACTTCACGAGGCATTATTACAAATGCCACTGTGAAG
TGTTTGGGGGAACTCTTGGTGATGC

.((((((.(((((((((.(((((((((((((.((((((......))))))..)))))))))))))))))))))).))))))...

**********GTTCCTTACAAGCACTTCACG***
****

miR395d

GCTGTCTCTTGGAGTTCCTCTGAGCACTTCACGTACGTGTGTGGCTAGTTCTATTCTGTA
TTACCCTTAGATAGAATAGTCTTAATTAAGTGAAGTGTTTGGGGGAACTTTCGGGAACA
AC

..(((..((..(((((((((..((((((((((.....((...(((((.((((((.(((.........))))))))))))))).)).....))))))))))..)))))))))..))..)))..

****
***********TGAAGTGTTTGGGGGAACTTT**********

miR395g

GAGTTCCTCTGAACGCTTCATATAAGAGGTTATCCATCTTCATTGAAGTGTTTGGGGGA
ACTC

(((((((((..(((((((((...((((((....)).))))...)))))))))..)))))))))

**TTGAAGTGTTTGGGGGAACTC

miR395a

TTGTCCACTGGAGTTCTCCTCAATCCACTTCAGTAGATAGCTATGGCTAGGCCTCATTGC
ATTGCACTGTTACATAACTGTGATCATGGGGCCAAAAGCTAGCTATGTATAGTGAAGTG
CTTGGGGGAACTCCAGTTGACAC

.((((.((((((((((((((.(((..((((((((((.((((((..((((.((((((((.(.((..((..((((....)))))..)))))))))))...)))))))))).)))..))))))).)))))
))))))))))).)))).

****
********************************GTGAAGTGCTTGGGGGAACTC**********

miR395f

TTATCGCGGGTTCCCTTCAATCACTTCACATGGTACTATTTCAAGGCCTACTATGTGAAT
TGTTTGGGGGAACTCTCGATGT

.(((((.(((((((((.(((.((.(((((((((((((((......))...)))))))))))).)).))))))))))))).))))).

**GTGAATTGTTTGGGGGAA
CTC*******

miR396d

ATGGTGGTTTAAGGAAACAAGGAGGAAGGTTTAGGGCTTTTTATTGGCAAATATGGAA
GGTTTATCTCAACTGATGGCTTCATGCTTCTCCACGGCTTTCTTGAACTTAGTTTGAATA
AGGTGAGCCTTCTTACAAGATCAAGAAAGCTGCGGAAAAACATAGATCCACTCAAAAT
TATGAACTTTCAACAGAGAGATAGATCTCCAAGGTAAGTTATCTAGCCATTGT

(..((((((..((((.(((.....(((((((.....)))))))..........((((.((((((((((...((((((.(..(((.((.(((.((((((((((((.(((.((..((...((((....)))))
..))))).))))))))))))).))).)).)))..).))).)))......(((....))).....)))))))))))))......))).)))))))))..)

****
********CTCCACGGCTTTCTTGAACTT***
****

miR396c

GCCATGTTTTTCCACAGCTTTCTTGAACTTTAAAAATTCATGTGTTAATTCCGTTCTCAA
GAATTCCTTTTCGAGAATTCACGTGATTAAACGAAGTTCAAGAAAGCTGTGGGAAAAC

ATGACA
..((((((((((((((((((((((((((((......(((((((........((((((.((((....)))).)))))).)))))))......))))))))))))))))))))))))))))...
****
*****************TTCAAGAAAGCTGTGGGAAAA*******

miR396h

GAATGGTCTTTTTCGTGATCTTCCACAGCTTTCTTGAACTGTGTTGTGTGAGGTTTCTCC
AAGTGAAGGTTTAAGATCCCTTATGCAACATAAATTCTTTGAGCACAATTCAAGATAGC
TGTGGAAAATCACTGAGATGATCTCGTTC
((((((((.((((.(((((.((((((((((.((((((..((((((((((((((..(((((.......))....)))..))))))))))))))......(((....))).)))))).)))))))))).
))))).)))).)))))..))))
*********************TCCACAGCTTTCTTGAACTG*******************************
****

miR396c-3p

TGCCATGCCTTTCCACAGCTTTCTTGAACTTCTCTTGTGCCTCACTCACTTTCATTACTG
GAGAGATATGCATCATCAGTGGAAGCTTATAGGGAGAGGAGTGCAAGAAGAGGGTCA
AGAAAGCTGTGGGAAGAAATGGCA
.(((((..((((((((((((((((((((.((((((((((.((((.(((.(((((...((((..((........))..))))))))))......))).))))...))))).))))).))))))))))))
))))))..))))).
****
*********************************GGTCAAGAAAGCTGTGGGAAG********

miR396g

ATGCTGTGTGTGTGAGATCTGAGCTCAATTTTCCTCTCAAGTCCTGGTCATGCTTTTCC
ACAGCTTTCTTGAACTTCTTATGCATCTTATATCTCTCCACTTCCAGCATTTTAAGCCCTA
GAAGCTCAAGAAAGCTGTGGGAGAATATGGC
..(((.((.(.(.((((..((....))..))))).).)))))....((((((.(((((((((((((((((((.(((((...((...........................))...))))).))))))))
))))))))))).))))))
**TTCTTGAACT
TCTTATGCATC**

miR396e-3p

GGTCATGCTTTTCCACAGCTTTCTTGAACTTCTTTGCCTTGCTTAATCTGTGTATATATAG
ATCACTACATGTACAGCTCCTATATATATAAATATATGTATGTATAGCGCCATGGAAGCTC
AAGAAAGCTGTGGGAGAACATGG
...((((.((((((((((((((((((((.(((((..((............(((((((.(((....))).)))))))....(((((((((((......)))))))))))).))...))))).))))))))
))))))))))).)))).
****
CTCAAGAAAGCTGTGGGAGA***

miR396g-3p

TTGCATGCTTTTCCACGGCTTTCTTGAACTTGGCACTCAAGAGACATGAGAGTAAAGG
GCTAGGCTTTCTTTTCTATTCCTTTCGTTCTTTGAAATTTTCCTGTTCAAAGAATCCATAC
ATATCAGTCTCTTTAGTACAAAGCTCAAGAAAGCCGTGGGAAAATATGA ptc-MIR396g
168
...((((.((((((((((((((((((((.(((.(.(((.(((((((.(((....(((((..((((........))))..))))).((((((((((.........))))))))))).........)))))))
))).))).).))).))))))))))))))))))).)))).
****

***CTCAAGAAAGC
CGTGGGAAAA*****

miR396

TTTTCCCACGGCTTTCTTGAACTTCTCATTCGGAACTGTTATCAGTGCGTCCGGCCGAT
GCCCACAGAACTCATCATGAAGTTCAAGAAAGCCGTGGAAAAATATAA
((((.((((((((((((((((((((....(((((((((....))))...)))))..((((..........))))..)))))))))))))))))))).)))).....
***TCCCACGGCTTTCTTGAACTT**
****

miR396-3p

GCTATTCCACAGCTTTCTTGAACTTTCTTGAACTGCTTTTTATAATTTTTGTATATAGCAC
CATACATGGAAGCTCAAGAAAGCTGTGGGAAAAC
....(((((((((((((((((.(((((.((...((((...((((.....))))...))))......)).))))).)))))))))))))))))....
**AAGCTC
AAGAAAGCTGTGGGA****

miR396a

TTCCACAGCTTTCTTGAACGTCTGTGCCCTTTCAGGAACTATCAGATAATTTTCATGCA
GTTCCAATAGCACAAAAACAGTTCAAGAAAGCTGTGAAA
...((((((((((((((((((.(((((.......((((((..((((......)).)).))))))....)))))...)).))))))))))))))))...
TTCCACAGCTTTCTTGAACGT***
****

miR396b-3p

AGATGGCCTTCTTTGTGATCTTCCACAGCTTTCTTGAACTGCATCTCTCAGAGGAGCGG
CAGCTTCAACTCCTCCACCCGCATCAGCAGGTGCATGCAGTTCAATAAAGCTGTGGGA
AACTGCAGAGAGAGGCCAG
...((((((((((((..(.((((((((((((.(((((((((((......((((((..(......)..))))))((((.((....)).)))).))))))))))).)))))))))))).)..))))))
).)))))).
****
*******************GTTCAATAAAGCTGTGGGAAA*****************

miR397b-3p

CCTGGGTTTGAATAAACATCATTGAGTGCAGCGTTGATGTAAGTTTACTTATTTTATTCC
ATTGTTGAATGAATTAAAATGATACAGAATACATCAGCGTTGCATTCAATTATGTTTTTCT
AATTTTCAGG
((((((...(((.((((((.((((((((((((((((((((((.......((((((((...((((....))))...))))))))........))))))))))))))))))))).)))))))))).....))
))))
****
**************TCAGCGTTGCATTCAATTATG*****************

miR398f

CCGGGTCGACTAGAGAACACACGAAGAGAAGTCCGCAACGAGTGGGGCGCTTCGTTT
CACTTTCCTGGGTGTTCTCAGGTCGCCCCTG
..(((.(((((.((((((((..((((.((((((((.((.....)).)).)))))).)))).(......).)))))))).))))).)))..
***GGTGTTCTC
AGGTCGCCCCTG

miR398b-5p

GGGGGCGGACTGGGAACACATGGGAATGAGATGAGATCATTGCTCGGTCGTGCTGGCC

TGGGCCGTCGGCGCGCGTTGATCTTGCATGTGTTCTCAGGTCGCCCCCGG
(((((((..(((((((((((((.........((((((((.(((.((.(((.((.(((....))))))))))).))).))))))))))))))))))))..)))))))..
*GGGGCGGACTGGGAACACATG**
****

miR398

AGCCCAAATTCGAAGAAGCATCAGAATGGGGAAGTCTCAACAGGAGCGATATGAGAA
CACACAATCATATGCATAAATATTGTGTATGTGTGTGTGCACGTGTGCATGTGCATGTGC
GTGCTTGTGTGTTCCCAGGTCGCCCCTGCCGAGCCTGCCCATATCTATAAATAATTCTTC
TTGCTGCT
(((.(((....((((((.....((((((((..((.(((..((((.(((((.((.(((((((((..((((((((((.....)))))))))).(..((((((((((....))))))))))..).)))))
))))).))).))))).))))..))).)).))))).)))........))))))))..)))
****
TGTGTTCCCAGGTCGCCCCTG**
****

miR399g

AGGGCTTCTCTCCATTGGCAGGCATAATGACTTAGACTTTCATGTTCTGCCAAAGGAGA
GTTGCCCTTC
(((((..((((((.((((((((....((((.........))))..)))))))).))))))..)))))..
AGGGCTTCTCTCCATTGGCAGG***

miR399h

CCATGCATTACTGGGCAGGTCTCCCTTGGCAGTGGCCGATCGAGCTGATCAAACCACG
CAAAAGCCACTGCCAAAGGAGACTTGCCCAGCAATGCAGAT
...((((((.((((((((((((((.(((((((((((.((((.....))))............))))))))))).)))))))))))))).))))))...
***TGCCAAA
GGAGACTTGCCCAG**********

miR399a

TGAGCGGATTAGTTGTAGGGCGTCTCTCCATTGGCAGGCAATGATGATTTTGTGTTCTTA
TAAGTTATGACCCGCCAAAGGAGAGTTGCCCTTCGACTGCTTCTGCTCA
.(((((((.((((((.((((((.((((((.(((((.((..(((((....(((((....))))))))))..)).))))).)))))).)))))).)))))).)))))..))))))).
***CGCCA
AAGGAGAGTTGCCCTT****************

miR399e

AACTAGTTAAATGGCTTCTCTTCTTTGGCAGGTGATGGTATAGATTACAACTTATAGCTT
TAATCTAGTATTCAGTTACCCGCCAAAGGAGAGTTGCCCTCTAACTTCC
....(((((...(((..((((((((((((.((((((.((((((((((............)))))).))))...)))))).))))))))))))..)))...)))))...
****
*CGCCAAAGGAGAGTTGCCCTC********

miR399i

AGTAGTTGTAGGGCTTCTCTCCTTCTGGCAGGAGATGGCAATAGATTATCCTTTGTGGC
TTATCTCCGGTGTTGATTAACTTCCAATGACCCGCCAAAGGAGAGTTGCCCTGTGACTA
CTTC
((((((..((((((..((((((((.((((.((((((((((.((((((......))))).)).)))))))((((((((.........)))).))).))))))))))))..))))))..))))))..
****
************CGCCAAAGGAGAGTTGCCCTG**********

miR399e-5p

ATGCCTAAGCTGTCCAGTTTCAGGGCTTCTCTTTCTTGGCAGGGAAGCATGAGATGCCA
TAGCTCTCTGCTCTCTTCAACCCTCTGCCAAAGGAGAGTTGCCCTGTAACAGGACTCA
.....((((((((((.(((.((((((..(((((..((((((((((.((((.(((((.((....)))))))))))..........))))))))))..))))))..)))))))).))).)))))..)))))))..
**********************GGGCTTCTCTTTCTTGGCAGG*****************************
****

miR408

ATTTAGTAGTAGCTAGGGAGAGACATTGCATGATAGCTACACCAAAGAGCAACCTCGG
CTCCTTGTGTGGCTTTGATGCACTGCCTCTTCCCTGGCTGCGGCCAAGTTA
((((.((.((((((((((((((.((.(((((.(.((((((((.((.((((.......)))).)))))))))).).))))).)).))).))))))))))).)).))))..
**TG
CACTGCCTCTTCCCTGGCTG***********

miR408e

AGAAGATGGGTATGGTTGGAGACAGGGATGAGGCAGAGCATGGGATGAGGCCATCAA
CAAAATTTCCAATTTCTGTCCTCCGCTAGGCCGCTACTGCATTTATGTTTGCTTGCTCAC
AAAACGGAGGGATTTGTGAGAGTTATCAGGCAGAAAGAACAAAGAAGGTGCCTCCCT
GGTGAAGTGGTGATGGCCTGACCTGAGACGGATGAGAGCTCAGCTGGTGTCCTGTTGT
TGCTTACTTCCCTGCACTGACTCTTCCCTGGCTCCCCACCGTTGCCCTTGC
.......(((((((((.((((.((((((.(((.(((.(((.((((..((((...(((((..((..(((...((((((.((.(.(((((((.(((..(.......((((((((((((((((.(.....).
))))))))......))))))))...............(((.....)))...)..))).))))))).)..))))))))(((....)))..))).))..)))))..))))...))))))).))).))).))))
)).)))))..))))).))))))....
****
****
****
******CTGCACTGACTCTTCCCTGGC******************

miR482a-5p

GAGAAGAGGGAAAGGGAGATTGGAGCTGCTGGAAGTTTTAGGAATGGGCTGTTTGGG
AAGAArGAAAATTACAAGAATAATTGTCTTGTGGGGTTTCTTCCCAAGCCCGCCCATTC
CTATGATTTCCAGCTGTTCCTCCCTTTCCCTTGTATCTC
((((.((((((((((((....(((((.((((((((((.(((((((((((.((((((((((((......((((((((.......))))))))...)))))))))))))..))))))))))))).)))))
))))).)))))))))))))))))...))))
***************************************AGGAATGGGCTGTTTGGGAAGA*********
****
******

miR482d

AGGAAGTTGTTGGAATGGAAGGGTAGGAAAGAAGAAACTTCTCTTGTATGTTCCAAAT
TCAAGAGATAAAATTTCTTTCCTATCCCTCCCATTCCTTCkATTTTCT
(((((((.(..((((((((.((((((((((((((((.....((((((((.((.......)).))))))))).....)))))))))))).))))).))))))))..).))))))))
*************AATGGAAGGGTAGGAAAGAAG************************************
****

miR482e-5p

GATCTGTGCTATGGAAATATTGATGGCGTTGAAAGGGATGAGAGTATTGGGAAGTTGTT
GGTTGTGGGTGGGGTGGAAAGATTTGATAAATCTAATTTTAAAGAGATTAAATCTTTCC
TACTCCTCCCATACCCATGACTTCCACTAGCTTCTCCCTTTCTTCATTTGTCATCTCCTTT

GCTAAGTTC
((.((..((...(((......(((((((..((((((((.((.(((....((((((..(.(((.(((((.(((((((((((((((((...(((........))).))))))))))))).))))).)))
))))).)..))))))....)))))))))))))......))))))))))...))..)).))
***TGTGGGTGGGGT
GGAAAGATT***
****

zma-miR482-3p
AGTGGGAGATGAAGGAGCCTTGCATCGATGTCACCGCCGGAGGAGCGCTCGCCTTCTT
CGCGCACCGCCGCAATAGCCGCCCTCGGACCCCTCGCCTCGCTCTTCCTTGTTCCTCCC
ATTTT
(((((((((..((((((....((..(((.(((...((((((((((.(....)))))))))).))...((.((....)).)).....)))...)))....))..))))))..).)))))))))..
****
*********************TCTTCCTTGTTCCTCCCATT**

miR535b
GATGTTGTGTGACAAGGAGAGAGAGCACGCTTGTCATCACCCATGAACGGATCATGGT
TGTTTACGGGCGTGCTCTCTCTTCTTGTCATACGGTC
....(((((((((((((((((((((((((((((((...((.((((((.....)))))).))...)))))))))))))))))))))))))))))))..
*********TGACAAGGAGAGAGAGCACGC**
****

Non-Conserved miRNAs

miR403c-5p
GGTTTGTGCGTGGATCTGAGGCCATCACAACCGTCCACTACACGACCACCCAATGGCT
TTAGATTCACGCACAAACTCG
((((((((((((((((((((((((((.......(((.........)))......))))))))))))))))))))))))))..
TTTGTGCGTGGATCTGAGGCC
****

miR414
TCATCATTATCATCATCATCATATTCATCTTCATCATCATCGTCATCATCATCATCATCATC
GTATGAGAAGATAGAGAAGAGTGAGAGTATGAGATTTGAGTTGAGA
...(((...(((..(((.((((((((.(((((.(((((.((.((((((...............).)))))).))).)))))))....)))))))))))).)))..)))..
************************TCATCTTCATCATCATCGTCA****************************
****

miR477e
TTGATCACTCTCCCTCAAGGGCTTCTATTAGTCATGTAGAGAATTCTTATGTAACTAGAA
GAACTTGAGGGAGAATGAACAAAC
(((.(((.((((((((((((..((((((.....((((.((......))))))....))))))..))))))))))).))).)))..
*******CTCTCCCTCAAGGGCTTCTA***
****

miR477a
ATTACTCACTCTCCCTCAAGGGCTTCTGTGAGTTTCTGGTTGAAATACCATACGGAGGA
CTTTAGAGAGAGTGGATAAT
((((..((((((((.((.(((..(((((((((......((((......)))))))))))))).))).)).))))))))..))))
********CTCTCCCTCAAGGGCTTCTG**

****

miR477i

AAGGCATTTTGATTGTTCACTCTCCCTCAAGGGCTTCCGGCAGTGCCAGACATATGCGT
GAATGGCGAGCTGGAGGTCCTCGGGGAGAGTGGACAATATTACTGTCT

.(((((.....((((((((((((((((..((((((((((((..(((((..(((....)))..))))).)))))))))))).)))))))))))))))).....)))))

******************ACTCTCCCTCAAGGGCTTCCG********************************
****

miR477b

CACTGCTTGTTGTCTCTCCCTCAAGGGCTTCTCTTCTGCTTGTTCATAAGATTTATAAGC
TTTTAATTAAGATGCTAAACTTAAAGCATACTGGGAATAGGAGCTCTTGCAGGTTGAGA
CAACAAGAAATGCC

.....((((((((((..((((((((((((((.((((((((((...........))))))).........(((((((........))))).)).)))).))))))))))).)))..)))))))))).....
...

************TCTCTCCCTCAAGGGCTTCTC***************************************
****

miR477h

AAGGCAATTCGCGTTTAACTCTCCCTCAAGGGCTTCAGCTAGCCAACGTCCCTTTGCAT
TCTCTTTCATGAGACTGGCGATGATGCACAGAGGAACGTGTTAAAGTTGAATCCCGGC
GGGGGGAGTTGGACGCGACCTGCCTT

((((((..((((((((((((((((((...(((.(((((((.....((((.((((((...((((......))))...((......)).)))))).)))).....))))))).)))...)))))))))))
)))))))..))))))

*****************ACTCTCCCTCAAGGGCTTCAG*********************************
****

miR477a-5p

CCTCTCCCTCAAGGGCTTCTCTCCTGCATGCTTATATGTTGGGAAAGAAGCTCTAGCAG
GGAGAGCCA

.(((((((((.(((((((((.(((((((((....)))).))))).))))))))).).))))))))...

CCTCTCCCTCAAGGGCTTCTC***

miR529

TGGTCGTGGCTGGCACCGTGGGTGGTGGTATCCCATGGCTGAGTACTTGACAGAAGAG
AGAGAGCACAGCTTGAGTCGGCGGCGACCCCTGTGAGACACCCGCCTGGAGGGAGCT
CTGGA

.((((((.((((((..((((((.........))))))((((.((.(((..(......).))).)).))))....)))))).))))))((...(((.(.(((.......))).)))).)).

***AGAAGAGAGAGAGCACAGC
TT**

miR827

TTTGCAGCAGTGGATATGTTTGTTGCTGGTCACCTAGTTATTCCATGGTTCCATCCATCT
GCTGGCTTCAATATATAATTTGTGTTTGTATATGTTGTATATATACATGCAGTATTGTATATT
TGACCCATGGCGGACGTTCTGATTAGATGACCATCAACGAACACATTCATGATGCAAG

.(((((...((((((.(((((((((.((((((.(((((((.....(((......)))((((((((.((.((((((((((...(((.(((((((((...))))))))).)))..)))))))))).))
.))..))))))).....))))))).)))))).))))))))).))))))..))))).

****
**TTAGATGAC
CATCAACGAACA***************

miR845a-3p

TCAATAGTTCCATCGCTCGGTTTCCATCACGTCGATTGGTGTCAGAGCCACGCCTATGA
AAAATTTGCGCGGCTCTGATACCAGTTGATGCAATGGAAACATCGATATTGCCTGA
......((...((((....((((((((..((((((((((((((((((((.(((..((.....))..))).))))))))))))))))))))..))))))))..))))...))....
**CGGCTCT
GATACCAGTTGATG*************************

miR845b-5p

AGTTCCATCACTAGGTTTCCATCACATCAATTGGTATCAGAGCAACGCATGATGTATAAT
AACTAAGCGTCGCTCTGATACCAAATGATGCAATGTAAACATGGATATTGCT
...(((((......((((.(((..(((((.(((((((((((((.((((.....((......))...)))).))))))))))))).)))))..))).)))))))))).......
**************************TCAATTGGTATCAGAGCAACG************************
****

>ath-MIR845a

AGATAAGGGATTGCACGATTTCTCAATAGTTCCGTCGCTGCGTTTCCATCACGTCGATT
GCTGTCAGAGCCACGCCTATGAAGAATTTGTGCGGCTCTGATACCAATTGATGCTATGG
AAACATCGATATTGCCTGATTCCGTGTTTCCTTTTACCTTGTTGT
....((((((..(((((...((.((((......((((....((((((((..(((((((((.((((((((((.(((..((.....))..))).)))))))))).)))))))))..))))))))..))))
))))...))...))))).))))))...........
****
************CGGCTCTGATACCAATTGATG***************************************
****

miR845c

CAATTATGTGTTTTGGTTGTCTGAGTTTCATGTTGGCAATTTCTTAATGATTAAACATGA
GGCTCTGATACCAATTGATGCATAAACCAT
...((((((((((((((.(((.((((((((((((...((((.......)))).)))))))))))).))))))))..)))))))))).....
***AGGCTCTGATACCAA
TTGATG**********

miR845d

ATAACCAACATTAATTATCCCTAGGGCCATGGGATAAACAAAAACTCTGCAAAACCAAG
TTAGCACACCCTAGAATGGTTCTAGCACAATTACTAACCTATGGCTCTGATACCAATTGA
TGGGAAAAACCT vvi-MIR845d 131
....((..((((((((.....((((((((((((.............(((..(((((..((((......))))..)))))...)))..........))))))))))))).....))))))))))........
****
*********************TGGCTCTGATACCAATTGATG**********

miR854

AATCAGTGGAGATGAGGATAGTGAGGAGGAGGAAGACACAGGAATGGGGGAAGTCGA
TGTTGAGAATGCAGGTCCTGGGATCACTGAGTAATGATTGTTGTAGGAATTTTGTAGGA
ACAGAGAAAAAGGTTTTCACTTTAAGAGACTGACACATACTTGAGTTATTACAGTTGG
GGAAATTAACATGTCTGTATTGTGCTCTCATTCTTTCTCTTGATATCAAATGATAAAAGC
TTATGTGGTTCTCTTTTCGAGTGTATTTTTGCTATTGTCCAAATCATTTTCCTCCTAAAGT
GATTTTGTAATGGAAATAAGTCATTTTTTTAAAAAAGGAAATCTAAAATCATTCTAAAAT
TTTAAAGAATCGCTTGGTAGTTGCAAATGCAGTGGTTAAACTCAAAGTATTTGCAAGTA
TAGCACAATGTACAAGAGCTTGCAAATATATCTTTGAGTGTATTAGTGAGACTATTTTGT

TAATAAGAGTATCCAATAGTTTTTATGTGAAGACACCAAAATTGGCCATCAAGATCATT
TCTTTCCTCTTCAGAGGCTCTTCTTCTCAACAATGTAGCATCTTCAGAGGACTCTTTC
..((..((((((((...(((.((..(((.((((((((....(((.((((((((..(((.((((....((.((((....))))...................((..((((((....))))))......(((((
(((..((((((((((....((((((..((((..((((((((.....))))).)))...((((((((............(((..((((....)))).)))).....((((((((.((((.(((((.(((((
..((((((.(.((((((((((((......)))))))))).)))))))).........((((((((....))))))))..))))))))).))))......))))))))...(((.((((((((((..(((.
.....)))..)))))))))).))).))))))))).((((((.((..(.(((((((......))))))).)..))....)))))))))..))))))....))))))))).))))))).)).......)
)..)))).)))....))))))))))....).))))))))))..)).)))..))))))))..))........
**********GATGAGGATAGTGAGGAGGAG**
****
****
****
****
****
****
****

miR858

CTTTTGCTCTCGTTGTCTGTTCGACCTTGAATTTTTCTCAAAAACATAGAAATATTATATA
TTCAGAACTTTTTTTTTTTTTTATCAATAAATAAATAAAATAAAACCCTCAATTTTGGATG
GATTCATTGGTAGGTTTAGGTAGCAGCCGAAGGACTATACAT
((((.(((...(((..(((..(.(((.((((((..((.(((((.....(((..........))).((...((((.((((.((((((......))))).)))).))))...))..)))))))..))))))..)
))..)..)))..)))))).)))).........
*******TCTCGTTGTCTGTTCGACCTT**
****
****

miR894

CATAATTCAAATAATCCAAAAAAAATAAACCAAATACCTTCCACCTCAAGCGTTTCACG
TCGGGTTCACCAAATATGTTGTATTATGTGAAGTTTTGATTGAGAATGCACCGAGCACT
TGGCTATGGAAGGTAAAAGAAAATTGCAACAAAAAGTCCTAACAATCCAATAGGATTA
TAGAAAATTTATAATTAAACGTTAACAATCATAATCAAACAATA
.....(((..((((((((................(((((((((......((((((((.(((((((((((..(((((...)))))..)))))..)))))))))).)))).((((....))))...))))))
)))...................((....)).........))))))).)))...................................
CGTTTCACGTCGGGTTCACC
****
****

miR1511

TCAGCCGTGGTATCAGGTCCTGCTTCATCAAGTGGTCTTGTGTTCAAATCCAGCCTCAA
GCACATGGTTAACCAGGCTCTGATACCATGGTGAATATAA
(((.(((((((((((((..((((.((.((((.(((..((((.(((.......)))..))))))).)))).)).))))..)))))))))))))))......
**AACCAGG
CTCTGATACCATG**********

miR1511-3p

ATCGTGGTATCAGAGTCATGTTATTTGCATGTTGCCACCACATATGTGTTGCTCATATAAA

TAACCTGGCTCTGATACCATAAC

...(((((((((((((((.((((((((.(((..((...(((....)))..)).))).)))))))).)))))))))))))))...

***ACCTGGCTCTGA
TACCATAAC

miR1863

GAATCGAATCTGAACAAAGGGAGTTCGGGGCGTGGTGGGTTACAGACAACGTGGGTT
TGGGGCGTGGTATTGTGACGGACATGCGTTTTTTGGGTATTTGGCCGTCAGGTTGCTAG
GGTGTTGAACGACGGACGGCGAACGGTTTTAGGGACGACAAACTGATGAGTTTTATGG
GGTTTTGTGGGGTTGGAATGGCAGATCTGAAAGCGTGGGTTTCGGGTTGGGGACATGA
GTTGAAGGGTTTGCAGCGGACATGGAAGGAGAAAGGGGCAGCGACGGTGGCTGTGG
GAGAAAGGTGGATGCGGCGGCGACGGTGACTGTGGAGAAAAAGGGGCAGCGGCGGG
TCTAAGGTTGGAGGGAGCGACGGCTGATGCGTGAAGCTTCGACAGGCGTGCGTGGGT
TTCTGTATTGCAAGAGACATGATTTTCAGATGAGGAGTGGGGGTTGGGGACAAGGGTT
TTTTTGTTTTTTAAGTGGAGACAAAAGGTGAAAGTGAGAGATTTACACTTGAGTATGTC
TCCGTCTATGCTAGTTATTGACGAAGGTAGAGATCGAACAAAGCGGCTAAAATCTAAA
GCTCTGATACCATGTTAGATTTGTATGAA

....(((((((((.((...((...(((((((....((((((.((((.(((((..(((((..(((......)))..)))))..))))).))))).....((((((((....(((((.(..((((....))))
..)))))).(((((((..(((.((((.(((..((((..(((((...(((((..(.(((.(....((((((((...(((....((..(......)..))....)))...))))))))..).)))..........
......((((..((.((.(((((.(.(..........).).))))).)).))..))))........((..((.(((.((.((((...(((((((...(((.((.....)))))....))))))))))).))))
)..))..))...)..)))))..)))))..))))......(((((....(((....)))....(((((..(((((....((....))......)))))..)))))....))))).))).))))))).........
........((....)).)))))))......)))))))))))))..)))))))..)).)))))))))))......

****
****
****
****
****
****
****
**********************AGCTCTGATACCATGTTAGATTTG******

miR1863a

CCATATCTCTAGTCTAATATGGTATCCGAGCTTATTGGTTAGTTGCTCTTGTTCCCTCAAT
CCCGTTTGTCTCCATCCACAACCACACGAGTGCAAAGCCTCACATTCATGTCAACAAC
AACTCTCATGTCATTAGCAAATCTAGTGTCTAGCATCAACACCTAGGTAGCACTTATTGG
TTAAAAAGGGCATTTGCTAGGAAAAATGAGCAACCACTAGTCCACTACACGTTTGGAT
GCTTGACAATATGTGCGCTTTGGTGCTTGCAATAATAATGTTGTGATGGAAGGTTTGTC
ACTAAATATTGGTTAGCAAAGTTGCAGTATGGGGAAGACCAATAAGCTCTGATACCATG
TTAGATTAGTGATATGCCT

.((((((.((((((((((((((((((.((((((((((((.(..((....))..)..............(((((((.(..(((((....((((..((((((..((((.......((((((........(.(
(((((.....)))))).)..(((....((((.((((.((((.(((((.........(((....)))..........(((((.(((...((.......))...))).)))))..))))).)))))))).))))..
)))........))))))))))..))))))..))))......))))).(((....))).).))))))).))))))))))))).)))))))))))))))))).))))))...

****
****
****
****

************************AGCTCTGATACCATGTTAGATTAG**********

miR2111a

CTTGATATTGGGTCAGGACTTGGTAATCTGCATCCTGAGGTTTGGATCATCATGTAGTTT
GATCTAGTCCTTGGGTTGCAGATTACCTCTTCCTCACTGACAATGCAGAG
(((..((((((((.((((...(((((((((((.((..(((.((((((((..........)))))))).)))..)).)))))))))))...)))).)))..)))))..)))
***********************TAATCTGCATCCTGAGGTTTG****************************
****

miR2111-3p

GCAATATTGGGTCAGGATCGGGTAATCTGCATCCTGAGGTCTAGATAAGTATATCTCCGT
TGCAGCTAGTCCTCTGGTTGCAGATTACTTCTTCCTCACTGCCAATGC
....((((((((.((((..((((((((((((.((.((((.((((....(((.........)))..)))).)))).)).))))))))))))..)))).))..)))))).
**GTCCTCTG
GTTGCAGATTACT*******************

miR2111b

GCACTTGATGAGGAACTGGTAATCTGCATCCTGAGGTTTAAAAATACATAGGCACATGC
AAATGTGTGTATTATAGTTTTTAATCCTCGGGATACAGATTACCTCTTCCTTTTACTGAA
.........((((((..(((((((((.(((((((((.((((((((..(((.((((((....)))))).)))....)))))))).))))))))).)))))))))..))))))........
****
ATCCTCGGGATACAGATTACC*************

miR2118b

ATGAGCGAGGAAGAGGAAGAAAGAGGTAGTCTAAGAGCAGTGGGAATGGGAACATA
GAGGAAAAATGAGCTCTCAGTGTTCACTTCTGGAAGTGGAGGTGGATATTTGTCTCAG
TATTTTTCCCGATGCCTCCCATTCCTATCGTTCTTAGGTGCTCCTTCCTCTTCCTTTCAGC
TCAT
(((((((((((((((((((...(((.((.(((((((((.((((((((((((.(((.(.(((((((((......(((((((((((((((......))))))))))).)))......))))))))).)
))..))))))))))))).))))))))))))))))))))))))))...))))))
****
TTCCCGATGCCTCCCATTCCTA********
****

miR3630-5p

CAGTATGGTTGTGCAAGTGACGATATCAGACAGGGACATCAGCACCTCCAATGGAGAA
TGATGATTTGTCTTTGGGAATCTCTCTGATGCACAAGCATATTG
(((((((.(((((((((.((.(((.((...((((((((((.((.((((...))))..)).)))..)))))))).)))))))).))..))))))).)))))))
***********TGCAAGTGACGATATCAGACA***************************************
****

miR5021

GTAGTGATGATGAATTTGAGATTTGGGAGAGGGAGAGGATTGAGAAGAAGAAGAAGA
AAAGAGTGATTTGAGAGTTCATGGGTCTTGAAAGATTTTAGTGACTTTCGTTATCTTCC
TTGTTGTTGTTCTTCTTCTCTAATAAGTTCTTCAATGGAGTTTTGTCGTCTCTAT
((((.((((((......(((((((.(..((((((....((((((((((((((.((.(((((...(((.((((((((.(((.(((((....)))))...))))))))))..)))...))).)).)).))
)))))))))).)))...))))))..).)))))))))))))).))))
TGAGAAGAAGAAGAAGAAAA********
****
****

miR5293

GGTTGAAAATGATGAAGAAGTGGAAGGAAGAAGAAGAAATGAAAACAAGGTTTTTAA
TTTGAAATTTTGTTAATCCTTGTTTTTGAATTTCTTCCCTCTATAAGAGGCATCATGTCAG
CCA

((((((..((((((.....(((((.((((((((.......((((((((((...(((((..........))))).))))))))))...)))))))).)))))......)))))).)))))).

**********GATGAAGAAGTGGAAGGAAGAAGA************************************
****

miR5298a

CGTTAGGTTTTCTTCATTTTCTTCTAGATTTTTCTTGGACTTTTGTTGTTGAAGGTCGAT
GGATATGATATGAAGATGAAGAAGAAGAGAAG

.......((((((((((((((.((...(((..((...((((((((......)))))))))..)))..))...)))))))))))))).......

**TGGATATGATATGAA
GATGAAGAA*********

miR5654a

TAATGATGTATTGTAGCTCTGCGTTTCTTGTTCGTTCACTCTTTTGTCAGGTATTAACAA
GATAAATCCCAAGCATCATCCACCTTATTAGCCCTTGAACATGAAGGCTTGTAAAATGG
ATGATGCTTGGGATTTATTTTGTACTCTCCTCTTCTAAAAGAGTGTTGCCTCCATAGGAC
TACCAATGATTTCAGA

...(((..(((((((((..(((.......((...((.((((((((((..(((.....((((((((((((((((((((((((((..((((.(((((.((......)).)))).)))))..))))))))))))
))))))))))))))).....)))....)))))))))))..))...)))))..))).)))))...)))..

**ATAAATCCCAAGC
ATCATCCA**
****

Novel miRNAs

Lc-miRn1

CAACAGTTGAGGGGAATGTTGTCTGGTGCGAGATCTTTCATCTCTTTTGAATGATTTCG
GACCAGGCTTCATTCCCCTCAACATCCC

.....(((((((((((((..((((((((.((((((((.(((((.......)))).))))))))).))))))))..)))))))))))))).....

************GGAATGTTGTCTGGTGCGAGA**
****

***TCGGACCAGGCTTCATT
CCCC**********

Lc-miRn2

GTTTTATTTGGGTTTCACTTGTTCTTCTTGCCACCAGCCAGAATCCTTTGGATTTGGAGA
CCTCTGCTGAATGCTTGGTTGAAGAGGAGCCTGGTCTGGAACTCCGACACAAAA

.......(((((((((((((.((((((((((.(.((((((((((.(((...))))))))))........((.....))))))).))))))))))..)))..))))).)))).......

****
AGAGGAGCCTGGTCTGGAACTC*******

Lc-miRn3

TGAGGACAAGCAATGGCTTAAGTTTGGGGGTGTGATAACGCTCATTTATGTGAGCTATT
TAGCCTTAAAGACCTAGGTTATTGTTTAGAAAGCAAGAACTTTGTGCTTT

.......(((((((((((((.((((.(((((..((((..(((((((....))))))))))..)))))..)))).))))))))))))))..((((((.........))))))

**********CAATGGCTTAAGTTTGGGGGT**

****

Lc-miRn4

AAGGGGTGTTTGGAATGGAGTTTGTTCCGAAATCTTTGTTGTTTCTTATTCTTTTTCTTT
GCAAAGTTTCTTTCTTTCTTTCTTCTTTATGATCTTGAAGATTCTATGTCGTTTTTTCCGA
CTAAAAGTTTGCCCTCTTCTTCGTCTTTGAAAAAATGATCTCGGACCAGGCTTCATTCC
TCGCACGTCTA

.(((.((((..(((((((((((((.(((((...((((((....................))))))................((((((......))))))......(((((((((((.(((.(((((.......
.))).)).)))...)))))))))))).))))).)))))))))))))..)))).))).

****
****
CTCGGACCAGGCTTCATTCCT********

Lc-miRn5

GGAGCTTTTTTCTTTTGAGGGGAATGTTGTCTGGCTCGAGGACACTTGTTTTGATCCAT
TTTTAATTCTCTGTTTCTTTCAATTTCCAACTTCAATTTGGTTCATTAGTGCCGTCGGAC
CAGGCTTCATTCCCCCCAATTGCTTGTCTCCATTT

((((.......(..(((.((((((((..((((((.((((((.((((.....(((.(((...................................))).)))..)))))).)))).))))))..)))))))).
)))..).....))))....

*******************GGAATGTTGTCTGGCTCGAGG****************************
****
****
****
**********************************TCGGACCAGGCTTCATTCCCC*****************
***

Lc-miRn6

TCCTCCGGAGCCTCGTGATGACGCTTTTCTGAAGGTCTCGCTCCGTCCTCTCCTTAATC
TATATGGTTTCATATGCGTGATTGATTATCGGCTCACTTTGGAATTGTGTGCGTTTGTGCT
TTTAACTGACAACTGACAATTTCCATGTCGTGTTTAGTTCTTTAGAAGAGTGCGAATCG
CAAACTGTAGGAATAGGAAAGTAGTATACCGTTTGTTGTTTAAGAACTATAGAATGAAT
GAGTTGAGTTATGGATGGTCGGAGGTTTCTCATGAAGGTGTTGTCATGCGTTGGAAGAT

((.(((((.((...(((((((((((((.((((((((((...((((((....((((((...((((((((((.....((((((((...)))).))))..))))))))))((((((((((((((((
((((..((.((((.......))))))..))))))......)))))))))))).)))))..((((((((((((.(((...........))).))))))).....)))))).........))))))....))))))
...))))))).))).)))))))))))))))).))))).)).

**********CCTCGTGATGACGCTTTTCTGA**
****
****
****

Lc-miRn7

CTGCCAATTGAGCTCTGTTGGATTCTCTTTGTAAGAGCCCTCATGATTCCTCTCTCTTGT
TTTTTTTTTTTTTTTTGAGAAATAATTTCAAAGCCTATGATCATGGAGGTTTCTGATCTTTC
TCAGGGAAAAAAAGAAGCCCAAAAAGTTTCAATCAAGGCTTACTAGGTTT

..(((...((((((.((((((((..(((.((((((((((((................)))))).((((((((((((((((((((((.....(((((((((..........)))))).)))..)))))))))))))
))))))))))..)))).))).))))).))).))))))...)))..

**********AGCTCTGTTGGATTCTCTTTG**
****

****

Lc-miRn8

ATTGTTGAGGAGAATGCGGTCTGGTTCGAGAGCATTCATTTGAATAAATTCAAAGAAA
AATCTTTCGTCCGAATGATCTCGGACCAGGCTTCATTCTCGCCAGCTCT

...((((..((((((((.(((((((((((((((.(((((.((((((....))))))((((....))))....))))).))))))))))))))).))))))))..))))...

************AATGCGGTCTGGTTCGAGAGC**************************************
****

**TC
TCGGACCAGGCTTCATTCT**********

Lc-miRn9

TTCTTTTCTAGCCCTCGGGCTGCAGATTACCTCTTCCTTGCTGGCAATGCAAGATTTGC
AACTCTTCTGCAAGGTTTCTATGCTCTTTTTCCTTCTGTTTTTATGTTATGTTCTCTCAAG
ATTTCATGTTATGAACATGGTGATGGTGATGATTCTTCTGTCACATGCTGCTGCAAGATT
AAAACATCACATCCAAGTAGGTAAGATTTTACAGAACTGATAGCAGAAATGTC

..(.((((((.((..((((.(((.((((((((((((((((((.((...(((((...)))))...))...))))))).............................(((((((...((........))....)))
))))((((((((((((((....)).))))).(((....))).........))))))....))).))))))...))).)))..))))..))))))).)..

**********GCCCTCGGGCTGCAGATTACC**
****
****
****

Lc-miRn10

TCAGAAGGTGCATGAAGAGCAAGATGGAGAAGCAGGGCACGTGCTGTACTAACTCCA
CTCAAATGCAATTCAAAAATCACTTCTTTTTTTTTTGGGGTTTTGAAAGAAGAGATAGA
TTTTCAAATTTCAAACAAAAAAAATTCAGCTTTGTTGCCTTTTGAGTTAGTTCTTCATGT
GCCCCTCTTCGCCATCGTGACCACTG

.(((..(((.(((.........(((((.((((..(((((((((..(.((((((((........((.((((..((((((...(((((((((((.........)))))))))))..))))))..)))).)).
..............((......)).....)))))))).)..)))))))))..)))).))))))))))).)))

************************TGGAGAAGCAGGGCACGTGCTG************************
****
****

****
****
************TCATGTGCCCCTCTTCGCCATC**********

Lc-miRn11

CCACCTATCTCAGATAGAGAACTATGGGACCCATCGCAGCCTGCCTTATAAAAGGGTTT
TGCAAGCGATTGCTCGACAGGACTTAGATCTGGTCTTTGAAGGAATGAAAAAAAGGA
GAGACTGGTAGAGCTCCTCTTTGTCAGCGGCATTCTCCCTTCTATCTATCAACCATTTCA
TTATGGAACTTTCTCCCCGAGCTGCGGAACTAACAAGTCTATTAGAAAGTCGAATTAGC
CACTTTTACACGAATTTTCAAGTGGATGAAATCGGTCGAGTGGTCTCAGTTGGGGATG
GGATT

...((((((((.(((.((((.((((..((((..(((((((...((((....))))(((..((((....))))..))).(((((.......)))))((((.(((..((.....(((.((((((((((((
.....)))))))))......)))))))))..))).))))((((......)))).............)))))))).........(((((((.((((((((................)).))))))).))))))).
.....))))..)))))))).))).))))))))...

****

****
****
****************************ATCGGTCGAGTGGTCTCAGTTGG**********

Lc-miRn12

CCTGAAGAGTCTTTGGTCTTCAAGGAGCGTTGAAGTCATGAAGAGTGTTTGGACTTCA
AGGAGTGTTCAACACTTAGACATTTATGGATTTCAGGGAGTCTTCAGT
.(((((((.((((((((..((((..(((.(((.((((..((((.(.(.((((.....)))).).).))))..)))).))).))).))))..))))))).))))))).
**********CTTTGGTCTTCAAGGAGCGTTGA***************************************
****

Lc-miRn13

ATGGCTCTGTTTGTATTCTTCCACAGCTTTCTTGAACTGCATCTTCTTGAGCCTAATTCG
CTCCTATCTCTAAAAGCGCTTTTGTTCATCAATGCTTTTGCGGTTCAATAAAGCTGTGGG
AAGATACAGACAGGGTCAACCAA
.((((((((((((((((.((((((((((((.((((((((((......(((((..((..((((..((....))..))))..)).)))))..........)))))))))).))))))))))))).)))))
)))))))))))).....
********************CCACAGCTTTCTTGAACTGCA******************************
****
****
***********************GTTCAATAAAGCTGTGGGAAG********************

Lc-miRn14

TGCTTTGGTTGGTGAAAGTGGAAGTGGAAAGTCTACTGTAATTAGTCTGATAGAAAGA
TTTTATGATCCCGACGATGGTCGTGTCTTGCTCGATGGAATAGATGTGAAAAGACTCAA
ACTTAGTTGGTTGAGACAACAACTAGGCTTGGTTAGCCAAGAACCAATTCTTTTCAATG
AGACCATCCGTGCCAATATAGCTTATGGCAAGCAGGGACAAGCCACCGAGGAAGAGAT
AATTTTAGCAACAAAATTAGCTAATGCCGACAACTTCATATCTTCGTTGCCTGGTGGTTA
CAATACTCTTGTGGGCGAAAGAGGTATGCAGTTATCTGGAGGACAAAAGCAGCGGATA
GCCATTGCG
.(((((........)))))...(((((...((((.((((..((.((((..((((..((((....)))).((((....))))(.((((.((((.(((........((((((((......((((((((......
....))))))))(((((....)))))........))))))))......((.....(((((.((.....)))))))).....)).....))).)))).)))).)(((((((......)))))))((....))....
((((.((((((((..(((((((..(((......)))....)))))))..)))))))).)))).))))..))))))..)))).))))..)))))...
****
****
****
****
**************TGGAGGACAAAAGCAGCGGAT**********

Lc-miRn15

GTAAGATTGAGAAAATTTTCTGGCATAGGCTACTTGGAAACTTGAATTCAAAGCAATTC
ATTCATTCTCCGAATATGAACTGGTCTTTAATTTCCAAGTCCACCCATGCCCGCTGATTT
TCTCATCTCTTCTA
..((((.((((((((((..(.(((((.((..(((((((((.(((((.....(.((.(((((..((((...))))))))).)).).)))))))))))))))...)).))))).)..))))))))))..)
)))...
********************TGGCATAGGCTACTTGGAAAC******************************
****
****

************TTCCAAGTCCACCCATGCCCGC********************

Lc-miRn16

CACTGTGTCGTCTCCCTCGTCGAGACCCACGATCCATAGCCACAGACGACCCACGGTG
CGAAACGCAGTGGGTTTCGTTTCAGATCGAGATTCCGTGGGTCTATTTGCGAGTGGGT
CTGTGATGTGGGGCAGAGGCGCTTTCCGTGCTGTTGGGTGT

....(((((.((((((((((((((((((((...........((((.((((((((((((((...))))...(((((((........)))))))))))))))..))))...)))))))).))))).)))
).))))))))..((((......))))...

**********TCTCCCTCGTCGAGACCCACG**
****
****

Lc-miRn17

CACGAATTGATCGATAAACCTCTGCATCCAGTTCTTTCTCTCTTTACGTTTGATTGATCT
GAAAACCCATGAAAATGAAAGAAACTGGTTTATTTCAATGCATTTGACTTAATTAGGCC
TTATTCTAGATGCCATTTTATTGGTTTTCTTATCCAAAGCTATTGATCTTCTTTGCGATGC
CCAGGTATTATTGTTTGTTTGGT

..(((((.((.((((((((((..((((((((.........((..((..((((...(((..(((((((.(..((((((..((((...((((((..((((.....))))......))))))...))))......
))))))..))))))))..))))))).....))..)).......))).)))))..)))).))))))))..)))))..

**********TCGATAAACCTCTGCATCCAG***
****
****

Lc-miRn18

GAGAAAGATGGAAGAAGAAGAAGAGTGGGCATAGAGCTGTTCTGGGTTTTCAAAGCT
CTGCATTTTTACTAGAAATAACAAGAATCCCGAGCAGTTTCTTTCGCTTTCTCAGGAAA
CAAACAAGAAAATAAGGGGTAGCAAAAGCAGAGGAATTGAAAGCAATTTCTGGCGAG
AGAAGAAGAAGGAAGGAAAGAAAAGCAATCGCAGTGCAGTGGAAGAAAATCTTTTG
ATGTATCTTTTTAAGAACAGGATAGCGTATCAGTCCTACACAGAGCTCGCTCTTCCTTCT
TCTATTAAACAAATTGTTTTTCTTC

(((((((((((((((((..(((((((((((.....(((((((.(((((((......((((.........))))........))))))).)))))))((((((((((((((..(.........).)))))..
........((....))..((((((((....)))))))).)))))))))).......(.((((..((...((.(((.(.((.....((((((..(((....)))...)))))).....)).)))).))...))..
)))).).....)))))))))))))))))))))))............)))))).

**********GAAGAAGAAGAAGAGTGGGCA***************************************
****
****
****
****

Lc-miRn19

GGAGATGGGCAGCGGGGAAAAGGCAGAGCATGAATGGAGTCCCCAACAGAGTGAAA
TCAACAAAGTTTGCCTTTGCCTTCCTGTTGTGCCTCTACTCATGCACTGCCTCTTCCCTG
GCTGCCCTCATA

...((.(((((((((((((.((((((.((((((.(((((....((((((...(((.....(((((.....)))))..)))))))))...))))).)))))).)))))).)))))).)))))))))..
.

****
*****************CATGCACTGCCTCTTCCCTGGC**********

Lc-miRn20

AACACTTTGTGAAAGTATTCTTTAATTGTAAGCTATCACTAGGAAGCATTTCATCAGAG
AAACTAATCCAGCATCATCAGGTGTTGTGCATGGGATTGAAGGAGCTCTACAACAGTGT
A
.((((((((((..(((.((((((((((.((.(((.((....)).))).((((....)))).......((((((((....))))))).)..)).))))))))))))).))))).))))).
****
**********ATGGGATTGAAGGAGCTCTA**********

Lc-miRn21

TCTCTCTGTGCCAAGTGATGGAAGCTGATGGCCAAAGATGTGCTTGTATTTTAGTAACA
GTTATAGATTCTTGTTTGGAGAGAGAA
(((((((...(((((....((((.(((.((((......((((((........))).)))))))))).))))..))))))))))).
**********CCAAGTGATGGAAGCTGATGGCC*************************************
****

Lc-miRn22

TGAAAGACACACTCACAAACTAAACTTTGTTATGAAACGCACATCTTTTCTTTCTTGTC
TTTACTAACTTGTTAATATTCCTTGAGGACAAACAATGGCTTAAGTTTGGGGGTGTGAC
AACAC
.......(((((((.((((((.((((((((((.......................((((((((..................))))))))))))).)).)).)))))).))))))).......
****
*************CAATGGCTTAAGTTTGGGGGT**********

Lc-miRn23

CGCTAAGCTATGCTCACTTTGGAATCACACTACAAATAATTATGTCACATATAAGCTCGA
AGATCGAACGAAAACACTTAGTCAAGTCCCACGTCATTTTCATTTTGGTAACGAGAGT
GCACTAATAAGGGAACAAGACCAATGTGTATGGATTAAAATCGAAGTATTAAACTAAAG
TGAACATATCTCCGTGG
(((..((.((((.((((((((((................((((((...))))))..((((...))))......((((((.(((...((((..((((((((((...........))))))).))......)))).
...))).)).))))).(.(((....))).).........))))))))).)))).))..))).
CTAAGCTATGCTCACTTTGGAA*
****
****

Lc-miRn24

TGAAAAATGACATAAAATCAGAATCAAACAATCAGCCAGTATATGTGTGCATAGAGATA
GAGAAAGTGAGAGACTAAATATCATGCAGATGACATTATGGGAAATAATTGAAATAAGG
AGGAGGTAAGAAGCTATAATATTATAAACTGAATTGGTTAAGCTTGACATACAACAGGT
ATACCCATGATATCCATAAGAGCCCCTGAACCAAGAGACAGCATAACAGCTTCAGCAAT
TCCAAGACCAGTAGCAAGTGCTAAAGAAGTGGATACAGAGGAAAGGAGCTTCTTTCC
TTGATTCTCTTCTATGCCATCAGTATTTGTTTGAACAATGTTTTTGTCGTCTGTACTCAT
(((.....((((((((((.((...((((((((.......((((..(((.(((((((...(((((((((.....)))..((((((((((((((.(((((((..............................))
))))).))))...)))....(((...(((((........)))))..)))))))))))...........((((..........(((......)))))))....((((.......((((....))))......))))
....((((((((((....))))))))))..))))))))))))))))..)))))))))))....)).)))))).)))......))).
**********CATAAAATCAGAATCAAACA**
****
****
****
****

Lc-miRn25a

TGCCATGTTCTCCCACAGCTTTCTTGAACTTCCAAAGGGTTTTTTTTTTTTAATTTTTGT
TGCTAATCTCTAGGAGAGATTGCATAGAGAAAAAAAAATGATCGAGCTGAGAAGAAGA
TCAAATAAAAGAGAAGAAAGTTCAAGAAAGCTGTGGAAAAGCATGGCT
.((((((((...((((((((((((((((((.((....))(((((((((((((.(((((..(((.((((((((...)))))))))))..))))).......((((((...((....))..))))))..))))
))))))))))))))))))))))))))))...)))))))).
**********TCCCACAGCTTTCTTGAACTT**
****
****

Lc-miRn25b

AGCCATGCTTTTCCACAGCTTTCTTGAACTTTCTTCTCTTTTATTTGATCTTCTTCTCAGC
TCGATCATTTTTTTTTCTCTATGCAATCTCTCCTAGAGATTAGCAACAAAAATTAAAAAA
AAAAAACCCTTTGGAAGTTCAAGAAAGCTGTGGGAGAACATGGCA
.((((((.((((((((((((((((((((((((((...........(((((............)))))((((((((......((((((((((...))))))).))).........))))))))........
.))))))))))))))))))))))))))).)))))).
**********TTCCACAGCTTTCTTGAACTT**
****
****
****
***GTTCAAGAAAGCTGT
GGGAGA********

Lc-miRn26b

ACCCATGAGTATCATCAAGAGACCTTGAACAATCTCAGGACTGGCATGCAAAATACTC
AAATCCTTTGCGCCGTCATGCTCGATACCAAGGTCTGTTACTCTCCCCTGTTATAATGTT
GGCTTGAAACTGATGAGTCATGCAATGATTCAAGTGTTTATCATTTGTTCTGGCTTGGG
TGATCTGAAAATGGAT
..((((.((.((((((..(((.((..((((((...((.(((.(((..(((((...........)))))))))))).)).((((..((((.((.(((.((........)).))))).)))).))))(((..
.((((((((...))))))))...)))......)))))).))))).)))))))).)...)))..
****
****
****TTGTTCTGGCTTGGGTGATCT**********

Lc-miRn27b

ATCCTGCTATGTATTGGTGATGGGTATATAACGCAGCTTGAGTAGTAAAGTCTTGTGCTA
TGAACGCATAAGCAGGTA
..((((((((((.((.((....((((((..((...(((.....)))...))..)))))))).)).)))).)))))))..
TAAAGTCTTGTGCTATGAACGCA*
****

Lc-miRn28a

TGGGGGTTTGTCAAAGGGTTTCCAACAGAATTTATTGGCATTCTGTCCACCTCCTTCTC
TACAGAAAAACCCAAAAAGGGTTCATGAAGAAACCAAAAAAAAAAATCCAAAAAAAGG
CCTTCACTTTCTATTGTTTTTTTCTTTCATTTGGAGGTGGGCATACTGCCAACTGAGCTCT
GTTTGGCTCCTCTTTGTAACACCCTC
.((((((.(..(((((((...((((((((.((((((((((...(((((((((((........((((((((((.....))))...(((((...((..................)).)))))...........)
)))))......))))))))))))...)))))).)))).)))).))))...))))))..).))))))

****
****
***********AGCTCTGTTTGGCTCCTCTTTG**********

Lc-miRn28b

TGGGTTTGTTTGGCAAAGGATTTCCCACAGAGTTTATTGGCATTCTGTCCACCTCCCAT
TTCAATGCTTCATTCAATTTTTTAAAAAATTTTTTTGGTTGTTTCTTTTATATGGGGAGGT
GGGCATACTGCCAATTGAGCTCTGTTGGATTCTCTTTGTAAGAGCCCT
.((((((.....((((((((..(((.((((((((((((((((...((((((((((((...........((.((((................)))).))............))))))))))))...)))))
).)))))))))).))).)).))))))..)))))).
****
**AGCTCTGTTGGATTCT
CTTTG**********

Lc-miRn29

TTGGATTGACCAAAGTATCGTAGGTTGTTATTTTCACATGTACATGATATTACAGAAATA
GTGCATATTTTATTTATAAAAAAACATAAATATTTAGAATGAGAGAAAAATAAAGATGAC
TAACCTAAGATGATGGGCAGTTCTA
..((((((.(((...((((.((((((((((((((...((((((................)))))).((((((((.((((...........))))))))))))........)))))))).)))))).)))
).))).))))))..
**********CAAAGTATCGTAGGTTGTTATT**
****

Lc-miRn30

CACCAGCAGTTGAAGCTGCCAGCATGATCTAAACTTCTTCTTGCTCTTCAGCCAAGGA
AAGATCAGATCATATGGTAGCTTCACCTTTTGATGG
((.(((.((.((((((((((((..((((((((...(((.(((((((((....)).)))))))))...))))))).))))))))))).)).))).)).
**********TGAAGCTGCCAGCATGATCTAA***************************************
****

Lc-miRn31

TGCATGTGAACTAAGTGCCTGCTTGGAATGACTTTTTTAGTGTTTAAAAACACTTTTTG
TTTTCACTTAAAAACATTTTTTTAAACACTTAGAAATTCATTCCAAACGGACTCTAAATG
TCAACATTGTT
.((((((..((..((.(.(((.(((((((((.(((((.((((((((((((...((((((........))))))....)))))))))))).))))).))))))))).))).).))....))..))).)
)).
**********CTAAGTGCCTGCTTGGAATGACT**
****

Lc-miRn32

TCCATGAGAAACGAGGATCAGCAGCAAACGAGATTGGATTGGAATCGGTGGAACGAA
CCCTAGGGAGAAGAAAGAGGAGGAGGAGGGAAGAAGGAAGGGGGCGGTGGTGGCT
CCGGCTCTTGACTTGGGAAAAGGCTGCTGCTGAATGTGCTGAGTTTGGTCGGTCATCG
GCTCAGTGTGATGGGTTCAACGCGTGGAAGAGTCTGAAGAGTTTTAAGAGTTGCGCCA
CTCACTTTCGTACGAATGCCCCACGTCGACTCCATCCTTATTGACTCATCTTTCAGATCC
GAACTCAAAACGAGGTCTGTTGTCCATACTGTCGTCCGTTCGTCGTGAAGCTGCTGCT
CGTCGTGG
.((((((....((((...(((((((..((((((.(((((..((....(((((.(((..(((..(((...((((((.((((((((.(((.(..((...(((((((((((((..((((((((((((.
(((.....((((.(((((((.(((((((((.(((.....)))))))))))).....).)))))..))))))).....))).)))....))))))))))).))))).))))..........)))))...))..

))))).)))))).....))).))))))...)))............)))....))))))))....))))))).)).))))...))))))))))))))))
****
****
****
****
*******CCGTTCGTCGTGAAGCTGCTG**********

Lc-miRn33

AGTCCGATTGGAGAACCGCAAGAGTCATGGAAGGTGGTCCTCACCTCCGAAAGGAGT
CTAGAAGATGAACGTCAACGCTTCTTGGAGCAATGCTCAGAACAGAGGAGGTGTTGG
ATGGATTCTTCGTGACTCTTCAGGTTCTTCGATATGCAT
......((((((((((..((((((((((((.(((.((((.(((((((..(((((((...((.(.....).))...))))))).((((...))))........)))))))..))))..))).)))))))
)))))..)))))))))))......
****
TTCGTGACTCTTCAGGTTCTT******
**

Lc-miRn34

ACTTCCGACGGGATGTTCACGTGGTGGGGCGGCGACCGGGACGGGGACCGGGATAGC
TTCCCGCTGTTCTGTGAAATTTGGCGGGGAT
..(((((.(.((((.((((((((((((((.(((..((((........))))....))))))))))...))))))))).)))))).
**TCCCGCTGTTCTGTG
AAATT**********

Lc-miRn35

TCCCCCTTCGTTCCAGATCTGGAATGAACCCAGATTTGGGCTCGTTCCAGATCTGTAAC
GAAGAGATGGA
(((.((((((((.((((((((((((((.((((....)))).))))))))))))).))))))).)..)))
**********TTCCAGATCTGGAATGAACCC**

Lc-miRn36

ACCCCTGCTCCTCGTCGTCGCTCAGATTCGACTGCTGCGGCTTGGGCTTCGCCGGATAA
GGGTATTTCACGACGGCAGGCGGCGGTTGCTGGTGGTGGTGGGGC
.........((((((((((((.(((...((((((((((.((((.(.....(((.......)))......).).)))..)))))))))))))))))))))))))).
**********CTCGTCGTCGCTCAGATTCGA**
****

Lc-miRn37a

CTTAGGACCTGGCCATGTTTTTCCACAGCTTTCTTGAACTTCTTCTTCTTTCATTTCTTC
TTCCTCGCAATTCTCCTCTGAAGATTTAATGAATTCTGAAGCTCAAGAAAGCTGTGGGA
CATCATGGCACTTCAGGCTTCTTT
...((((((((((((((...(((((((((((((((((.((((.......((((((...(((((................)))))...))))))....)))).)))))))))))))))))...))))))..
..)))).)))))..
*********************TTCCACAGCTTTCTTGAACTT*********************************
****
****
************************CTCAAGAAAGCTGTGGGACATC*********************

Lc-miRn37b

TGCCATGATGTCCCACAGCTTTCTTGAGCTTCAGAATTCATTAAATCTTCAGAGGAGAA

TTGCGAGGAAGAAGAAATGAAAGAAGAAGAAGTTCAAGAAAGCTGTGGAAAAACAT
GGCC
.((((((.....((((((((((((((((((((....((((((...(((((...(........)...)))))...)))))).......)))))))))))))))))))).....)))))).
**********TCCCACAGCTTTCTTGAGCTT***
****

Lc-miRn38

GGGGAATGTTGGCTGGCTCGAGGCTTTTCAAAAGAAAGATGTTCTTTTTCTTTTCAAGT
TTAAAGGAAAAAAAAAAAAAAGAAAAAAAAAAAGAGAACTGTTAGGCTTCGGACCAGG
CTTCATTCCCCTCAACTCAAA
((((((((..(.((((.((((((((((..((..........(((((((((.(((((.........................)))))...))))))))))..))))))))).)))).)..))))))))......
....
GGAATGTTGGCTGGCTCGAGG
****
****
**************************TCGGACCAGGCTTCATTCCCC**********

Lc-miRn39

AGAACCTGGCCATGTTTTTCCACAGCTTTCTTGAACTTAAAATTTCTTTCATTCTCACAA
ACAAACTACAATCTCAATTCTAACAAAAACACTTTCAAGCTCAAGAAAGCTGTGGGAA
ATTATGACACTTTC
.(((..((..((((.((((((((((((((((((((.(((.(((..))).))).)))))))))))))))))))).)))).))..)))
**************TTTTTCCACAGCTTTCTTGAACT**********************************
****
****
********************CTCAAGAAAGCTGTGGGAAATTA**********

Lc-miRn40

TTTTACTTTAATAATTACTACTACCACCCATAGTTTCTTCTTCTTCTTCTTCTTCTTTT
TATTTATTATTTTCTTTTAACATTACTACTTAGTGTCTGAATTGAAATTTACTGAATTGGAT
GTTATTATGCCTCAAATTTTATCATTTTCGTTGTTGTTTTTTTTAGTGTAAGTTTTCTTCGG
AGGTTGGGTTTGGTTTTGGATTATTGGGAGATATGGAGATGTGAGTGGGATTGGGTGTT
GTAACGTAATTAAGGAAGAAGGG
.....((((..((((((((((...((((((..((((((((....(((((....((((((....(((((..(((................(((((((.((((((..((((((((((((...((((..(((.
.......)))..))))...)))))................)))))))))).)))))).))))))))..)))..)))))....))))))...)))))...))).))))))))))))..))))..))))))))..)))
).....
**********ATAATTACTACTACCACCCAT**
****
****
****

Lc-miRn41

TATAGCAAAACTATCGCTGATAGACTCGTATTGCTGATAGATTCGTATGATATATCAGTGA
TAGGTCAATATTTACAACATAGTCTATTTGTGACTATAC
..........(((((((((((((...((((((....((.....))))))))..)))))))))))))...............((((((.......))))))..
**********CTATCGCTGATAGACTCGTATT**
****

Lc-miRn42

TCATTTCTATGGCAGCATCTCAAGGTTGGTTTTGCACATTAAAAAAGTCTCCACATGAT
CCTTAGGAGAAGTATATATGGAGCAACCACCTGGATTTGTTGCTCAAGGGAAAAA
...(((((.((((((((..((.((((.(((..(((.(((........(((((............)))))......)))..))))))))).))..)))))).)).)))))...
****
CAACCACCTGGATTTGTTGCTC*******

Lc-miRn43

AATGAAATTAGGGTTTGAGGCAGAGCTCCTTGAAGTCCAATAGAGGTTTATGCCAGGT
TGATTGAGCTGCTAAGCTATGGATCCCACAGCCCCTATTGCTATATCTATCATGTTGTTGT
ATAATATCTTTAAAGCTGATAGGCCTGTGGCTTGCATATCTCAGGAGCTTCATCACCTGT
TTTTGTTAGATCCTTGTTTGGATTGAAGGGAGCTCTACATCTTCTCTCTAGTTTATC
....(((((((((...((.(.((((((((((..(((((((..((((((((.(((((((.(((.(((((.((.((.((((.(..((((((..(((((((((.((......(((((((...))))))).
..)).)))).))))))).))))))..).)))).)).)).))))).))))))))).....)))))).))))..)))))))..))))))))))).).))....)))))))))...
********************CAGAGCTCCTTGAAGTCCAATA*****************************
****
****
****
****
****
*************************************TTTGGATTGAAGGGAGCTCTA*************
****

Lc-miRn44

TATGGGTATCAGAGAAGTTTGTTTTAGTCAATTCTACAACGGTTTACTTAATTTCTAATG
CTTGTGGTCTACATTTCAAGTTCCGTTGTAGTCTAGTTGGTTAGGATACTCGGC
..((((((((............((((((((((((((((((................(((((.(((....))).))))).)))))))))...)))))))))))))))))..
****
TCCGTTGTAGTCTAGTTGGTTAG********

Lc-miRn45

TATAGAAAACTTTCAAATTTTATCAATGATAGACTTTTATTAGTGTCTATTACTGCATATA
ATAGATATTAACAGACTTTTATTAATGTCTATCGTTGATAGAATCTGATAATTTCTTAT
...(((((....(((.((((((((((((((((((..((((((((((((((((.......))))))))))))))..........))).)))))))))))))))))).)))...)))))...
****
********GTCTATCGTTGATAGAATCTGAT***********

Lc-miRn46

TTTGTTGAGGAGAATGGAAACTGGTTCGAGAACGTTTGTATGAATGAGCTTAAAAAGA
GAATCTTTCATCCGAATGATCTCGGACCAGGCTTCATTCTCGCCAGCTCT
...((((..(((((((((..(((((((((((.((((((.(((((.((.(((.....)))..)).))))).)))))).)))))))))))..)))))))))..))))...
***T
CTCGGACCAGGCTTCATTCT**********

Lc-miRn47

GACGAAGACGAACGTGAATCAGAGTTGAGAACGGACGTGTGACGGACGCGCGATTCT
GGGTTCGACGGATCTGGGTTCGTCGAATTTGGGTTCGTCGGATCTGGTTTGCAAGAGT
CCAACATGTGGATTGTTGAACGGAGACGAGGTTCGTGACTGTGAGATATATTCTTCAGA
TCTGAAAAAGAGACTTGCATACAGAGACCGATAGGGGACGACATGAGATCTAATAGTC
GTCAATGGCTTCACGGCAGAGATCTCGACAGCGGCGGCAAGAGGTACGCTGGATCTCG

ACGGCGGCGACTATACAATAACCCTAGGTTAAAAGTTCTGATACCATGTTAATCAATATG
......((..((((((.(((((((((...(((...(((((.....)))))(((.((((((((((((((((....)))))))))))))))).)))((((.((((...((((((.(((((.....))))
)((((..((((..((((...))))..))))..))))...........(((.....)))..))))))..))))..))))((((((((((((((........)).))))))..(.(((.(.((...((((((..
..(((((((........)).)))))))))))..))).))).)...........))))).)))...)))))))))..))))))..))......
****
****
****
****
TAAAAGTTCTGATACCATGTT*******

Lc-miRn48

CGAGTTTAAAAGTAAAAGATAAGATGTGTCTAATAGTAAGCTTGAAATGAACTACAAC
AACGGTATAGATCTCTCTCACATCTCTTCTTCTCTATAAAGCTGGCAAGCAAATTGTGTA
AGTTTTTGTATAGAGTGGTCCAAATTATGGGATATACTTCTTTTTTTTCTTTCTATATTCCA
TTTTTACCCATTCTACTTCTTTCTTCCATCATCTTCCTCTCCTCTCCTGTCTCAAGAAAGA
CATTCTTCATCTTCTTCCCCTTCTTCTTCTTCTGAAGATAAAGAAATCAAAAGGAAATG
GTTTTTTCAGGTCATAGCCATGCTTTTCCACAGCTTTCTTGAACTTTCTTTGCTCT
.((((...(((((..((((.(((.((((...((.((((.((((((..((((.(((((.(((..((((((....................))))))..(((....)))...........))).)))))(((((
(((....((..((((((((((.................))))))))))..))..))))))))............(((((.(((((.........(((((......)))))(((((.((((((.............
.....)))))).)))))......))))))))))...)))))..))).)))..)))).)).)))).)))))))..)))))....)))).
****
****
****
****
*********TTCCACAGCTTTCTTGAACTT**********

Lc-miRn49

TTACATTTGTTTTGGTGTGGTATGAAGCTTTTGATAGTTGAACCTAAAATCCGTTATCTA
ACTCAATTTTTGCAAAGCTTTCTTGCACTAACAGAGTAC
.(((.(((((..(((((((((...(((((((((((.((((((....................)))))).))).)))))))).)))))))))))))))).
**TTGCAAA
GCTTTCTTGCACT**********

Lc-miRn50

GTATACAAATTCCGTTGTAGTCTAGTTGGTTAGGATATTCGGCTCTCACCCGAAAGACC
CGGGTTCAAGTCCCGGCAACGGAAACTTTTTTTTAATATATTTTTATATAATGATATTAAT
TATTTGTTTTTGTATACCTATTTATTTATTAAAAAATATATATGAGAAAAGCACATTTGGA
GAATATCAAATTGGCCTAACTGGCGCCTGGCTGGAATCAAAATGCA
((((....((((((...(((.(((((((((((((((((((((((...(((((.......)))))...))))(((....)))...((((((((..((((((((((((((((.((((.........))))
.)))))))................)))))))))..))))))))..........)))))))...)))))).))))))...)))..))))))....)))).
**********TCCGTTGTAGTCTAGTTGGTTAG***************************************
****
****

Lc-miRn51

CATGTCACGGATGCAGGTTTGGAAGAAGAAGTAAAGGATAATTGTATTTTAAAATTCAA
CTCAGCCAAGTGAAGCATAAACCAATTAATCTTCCAACATTCGTCTTCTAGATCCTTGG
ACTACATA

.((((...((.(.(((((((((((((.(((((.((((.((((((...((((...((((.((......))))))...)))).)))))).))))...)).))).))))))))).)).)).))))))).
****
****************ACATTCGTCTTCTAGATCCTT**********

Lc-miRn52

AATAATGTCTCTCTCTTGAATGATGACTATAAATATGTGTTTCTCTTTCATGTTATTCTGT
GAAGAAAAAGGTGTCAATGTCATGCATGAATTATTGTCATTGAAGAACAGTGGAGAGA
GAATGATAGTCCATTGTTTAAGAGATAATTGTATTGTA
.((((((....((((((((((((((((((..........(((((((((.((((.(((.((((........(((.((......)))))).........)))).))).))))...)))))))))..))))).)
)))))))))))))......)))))).
**********CTCTCTTGAATGATGACTATA**************************************
****
******

Lc-miRn53

GTTCCGGAAAAGTAACAATTTAGTCTCTAAACTTTAGTTTCAAACAATTTAATTTTGGTA
TTTTAACTGTTGTAATAATTTAGTTTTTGAGCATACTATATATTAATTTAGTCTTTGTACTT
TGTTGTTGTTACACTTTGCCTAGAATGAGTAGTATGTTGAGAAATTTTAACGCCTAGAC
AATTCATGTGCTAATTTGCCTTGTAACTCGTTTATTGAGTTGGTATTTCGTAGGAAA
.((((....(((((.((((((((((....((((...((((....((((....(..(((((((......((((..........(((((((.((((((((((...(((.(((((.(..(((((.........).)
)))....).))))).))))))))))))).))))))).........))))......)))))))..)...)))))))).))))))))))))).)))))....)))).
**********AGTAACAATTTAGTCTCTAAACT*************************************
****
****
****

Lc-miRn54

AGGTGCGTGCCGATCAGGATGGAGAAGCAGGGCACGTGCATTACTCACTCGTGGAAG
TCCTAACACAACACCACCGGTGGCTGTGTCTGAGTGAGTGGGTTGTTCATGTGCCCCT
CTTCTCCATCCTGACCACTG
.(((....)))..((((((((((((((..(((((((((.((.((((((((((...((.....(((((.((((...))))..))))))).)))))))))).)).)))))))))..)))))))))))
))).....
******************TGGAGAAGCAGGGCACGTGCAT*************************
****
****
************************TCATGTGCCCCTCTTCTCCATC**********

Lc-miRn55

TTGCCGAGAAGAACAATAGCGAAGAGTCCATTCGCGCTAAATCAATTCCCGCCAAAAC
TTGAGATTTTTTTTCTTTATTTTAGGGTTTGATTCACCGATGGGCCCAAGGCCCAATCCA
GTCCACGAAAACAAAGCTTGAGTTTGGCCCAAATATCTATAACTATTTGTAAGGAGTAT
TTGTAGGGGATAGTTGCAAATTTACCATTTAAATTTAAATTAATTAAGTATATAGCATAAT
TTTAAAAAAATTACAAATATAGCAAATTTTGTCAAATTCTATCTGGGCATGCTTCCTTTG
CTTTGCTCTTCTTCTTCA
.....(((((((....(((((..((.....))..))))).........((((((.((((((.(((.(((((.........((((...........((((((...)))))))))).......))))).))).
))))))))))).((((((........))))))...((((((...((((((((.((((((.......(((..(((((((.....((((...(((((....(((((((...)))))))...)))))...))
)).....))))).))..)))))).)))))))))))..)))))))))))))..
****

****
****
CATGCTTCCTTTGCTTTGCTC
****

Lc-miRn56

TGGGGGTTGTGAGGAATGTTGGCTGGCTCGAGGCTTTTCGAAACAAAGATGTTCTTTT
TTCTTTTTTTCCTTTTTTTTCAACTTGGAAAAGAAAAAAGAAAAGAAAAGAAAAGAAA
AGCAAAGAAGAGAAAAGAAGAGAAAAGAAGAGAGAACTTTTAAGGCTTCGGACCAG
GCTTCATTCCCCTCAACTCAAA

...((((((.(.((((((..(.((((.((((((((((........((((.((((((((((((((((((..(((((((((..(((...............................)))..))))))))
).)))))))))))))))))))))))))))))))))).)))).)..)))))).).))))))...

****
****
*****TCGGACCAGGCTTCATTCCCC**********

Lc-miRn57

CTGAAGGTTTAAATCTGCAGTGTGTTACTTAAAACCTGAACTCTTCTTTGTATAACGTTT
TCATAATAAATGGATAGTTTTCCCCTTAACTAGTTAAATACACTTGCTATTTAGTTTTTAT
TTATTGTTCAGGGGGAGTATTTGAAACAAAATATTTACAATTACTCTCGCGGAAAGTTAT
CTAATACGTGCGTCGATTTCTTACCCTTTTT

..(((((...(((((((((.(((((((..(((...(((.........(((((.(((...(((((.....)))))..)))(((((((.(((....((((((.(((........)))...))))))..))).)
))))))((((((......)))))).))))).........)))....)))..))))))))))..)))))).....))))..

****
****
***********************CTAATACGTGCGTCGATTTCT**********

Lc-miRn58

CCTGTTAAACCAAGCTCTTGGTGACTGTTTCAACCCATACAACAACTTTTGAAGCTTGC
AAACCTGTTTGTCAAATTGAACTTGAAGTCTTGGAGGATGACTTACATACACTTTCTTC
TAAATCTACATTCAAGAAAACATTCTTAAAATCAAGTTGATATTGAGGCCAATCTTTATT
TGCAGCAACGGACAACAAGACTCTAACAGTGTTTAACTTGACAATAGGAAAAAAGTC
TCAGAATAGTCAACCCCATAAGTTTAAGTGAATCTT

..........(((((..((((((((((((.................(((.((((.....((((((.(((((.(((((((((.((((((((((((...............))))).............(((((
....)))))........((((..(.(((((.....))))).)..))))..........)))))))....))).)))))).))))))))))....))))).))))))))))))...)))..)))))........
...

**********CAAGCTCTTGGTGACTGTTTCA**
****
****
****

Lc-miRn59

TGAGAAAGTCTTCGGACATCGGCGACTTGGAAACTAGAAATTGAAACTTAGTGCAAAA
TTCATTCTGTTCTTGTGAATTTGAGCTGTTCTTTAATTTCCAAGTCCACCCATGCCCGCT
GATTTTCTC

.(((((((((..(((.(((.((.((((((((((.((((....(((...((((...((((((((.........))))))))..)))))))))))).))))))))))..)).))).)))..)))))))))

****
****************TTCCAAGTCCACCCATGCCCGC**********

Lc-miRn60

CCTAAAATTCTTAATCTTTCCAAAGATTTTTACCTTCTTTTCCTTATAAACTCCATTTTCT
CTTGTCCTTTTCAAAATATAACAAGTTTCAAAGATCTAAAAAAAAACAAAATTTGGAGT
TTTCACAACTTTGAGGTTTTCACACATTATATCCTCAAAAGGGTTAAAGGTTGACTCAC
AAAAGTTAGAGGCAATTGAAACTTGGGAATGCTCCAAAACATTTTATGAAGTTAGAAT
CTTTCTTGGCTTGGCAGGTTACTATAGGTGATTCGTGGAAGGATTTTTCAAATTAG
.((((.......((((((((((..((...((((((......(((.................(((.......))).......((((((..((((((((((...........((((((((.(((.....((((((((
................))))))).(((((...(((((.(((..........))).)))))..)))))..))).))))))))............)))).))))))...))))))..)))......))))))..)
).)))))))))).......))))
****
****
****
************************TAGGTGATTCGTGGAAGGATTT**********

Lc-miRn61

TTTGTAATTACAATGTGGGATCCTCAACATAATTGACACTCTGTTTCTTGTGCTCTGATA
TGTTTGAATAGCCATTGCCTAAATTTTTATTTTTGGCTGATCAAGGTATGAAGGCACTGT
TTGGGATGATTTGGCTCATGGTAAAGGTGTTTACGTTGCTGAACTGGGACTGGTCAGGT
CGGTCTGTACTTCATCATATCGCTTAGGCAGTATTGTACCTAAAATCTTTAATATCTTGTT
ATTAAGTTTATTGGTTAAAGATATTATAAAAGACACATTATGAAGAATTGGGGAAGATGT
TGTGAATTTCACGTCAGTTTTATGAAT
(((((((.....(((((((((.(.((((((........(((((.((((((((((.(.((((..((((.((((((..................)))))).)))).)))).).)))))..((((((((((((
(((.((..(((((..((((..((((..(((((.((((......)))).))))).))))...)))).)))))...)).))).))))).)))))).((((((((((((...(((((.....)))))..)
))))))))...)))).........))))).)))))...)))))).).))))))))).....))))))).
**********CAATGTGGGATCCTCAACATAAT**************************************
****
****
****
****

Lc-miRn62

TGAGGTTGATGGGAATGTTGTTTGGCTCGAGGTTCATATATCAAACTATATATCAACTTT
TTTGGATAAAGAAAAAGGGTTGTTGTTTGATTGTTAAATATATAATCTCGGACCAGGCTT
CATTCCCGTCAACCAAA
...((((((((((((((..((((((.((((((((.((((((..(((...(((.((((((((((.........)))))))))).)))......)))..))))))))))))))).))))))..)))))))
)))))))...
****
**************************CTCGGACCAGGCTTCATTCCC**********

Lc-miRn63

CCAAGAATAACAGTGCGAGTCTCTATTGGCAGAGATTTATTATTATCTCAAAGAAAAAT
AAAAAAATATTTTTGTTTTTGCTTCTTTTTTCGATCTTCTCCTTCTTCTGCCAAAAGAGA
CTTGCCCTGCCATTCCATT
....((((..(((.((((((((((.((((((((((.........(((..((((((((((((((....)))))))))....))))))....)))........)).))))))))).)))))))))).))).
.))))....
****
****************************TGCCAAAAGAGACTTGCCCTG**********

Lc-miRn64

TTTGAGAGGTTGAAGCTGCCAGCATGATCTGAGGAACGCTAAAACATTTGGTTAAAGA
TTGGTTTCTTCCATCAGGTCATCTTGCAGCTTCAATCACTCAAT

.(((((.((((((((((((.((.(((((((((((((.((((((((.....))).....)))))...)))).))))))))))).))))))))))).))))).

**********TGAAGCTGCCAGCATGATCTG***************************************
****

Lc-miRn65

GAGAGGCTACATCATACCATAAGTTTCTTTGCAATTTTTGTATTTTACACGGTTGACTTG
TTGATGAGCAGAACAAGTGCCCCACAATCGTCTGTCCCGTGTTTGAAAGATTGCGATT
GTGACAAGTGGTATCAGAGCCTAAG

...(((((.....(((((((..(((.(.(((((((((((.......((((((..(((..((((..(.(((.......))).)..)))).)))....))))))...))))))))))).).))).))))))
))...)))))...

****
*********************************TGCGATTGTGACAAGTGGTATC**********

Lc-miRn66

TGGGTGAGAAAATTCTTGTTGGAGAAGCAGGGCACGTGCAAACTCACCCATTTCTAAA
CCCTTTGCACGTGCTCCCCTTCTCCAACATGGCTTCCT

.(((.(((.....((.(((((((((((..(((((((((((((..................))))))))))))..))))))))))).))))))))

*******************TGGAGAAGCAGGGCACGTGCA******************************
****

**CACGTGCTCC
CCTTCTCCAAC**********

Lc-miRn67

TATAACAAAACTATCACTGATAGACTTGTATTGCTGATAGACTTCATATGATATATCAGTG
ATAGATCAACATTT

..........((((((((((((...(((((....(((......))))))))..))))))))))))..........

**********CTATCACTGATAGACTTGTATT***
***

Lc-miRn68

AGCTTGTGTTGAAGCTCCATTTGAATCAATGGCCTTCGTTTCCGTTTGGCCTATGTTTCT
TTGATTTGGAGGGAGCTCCTCTATACGGC

.(((.((((.((((((((.((((((((((.((((..((....))...))))).........)))))))))).))))))..)).)))))))

***TTTGATTTGGAGGG
AGCTCC**********

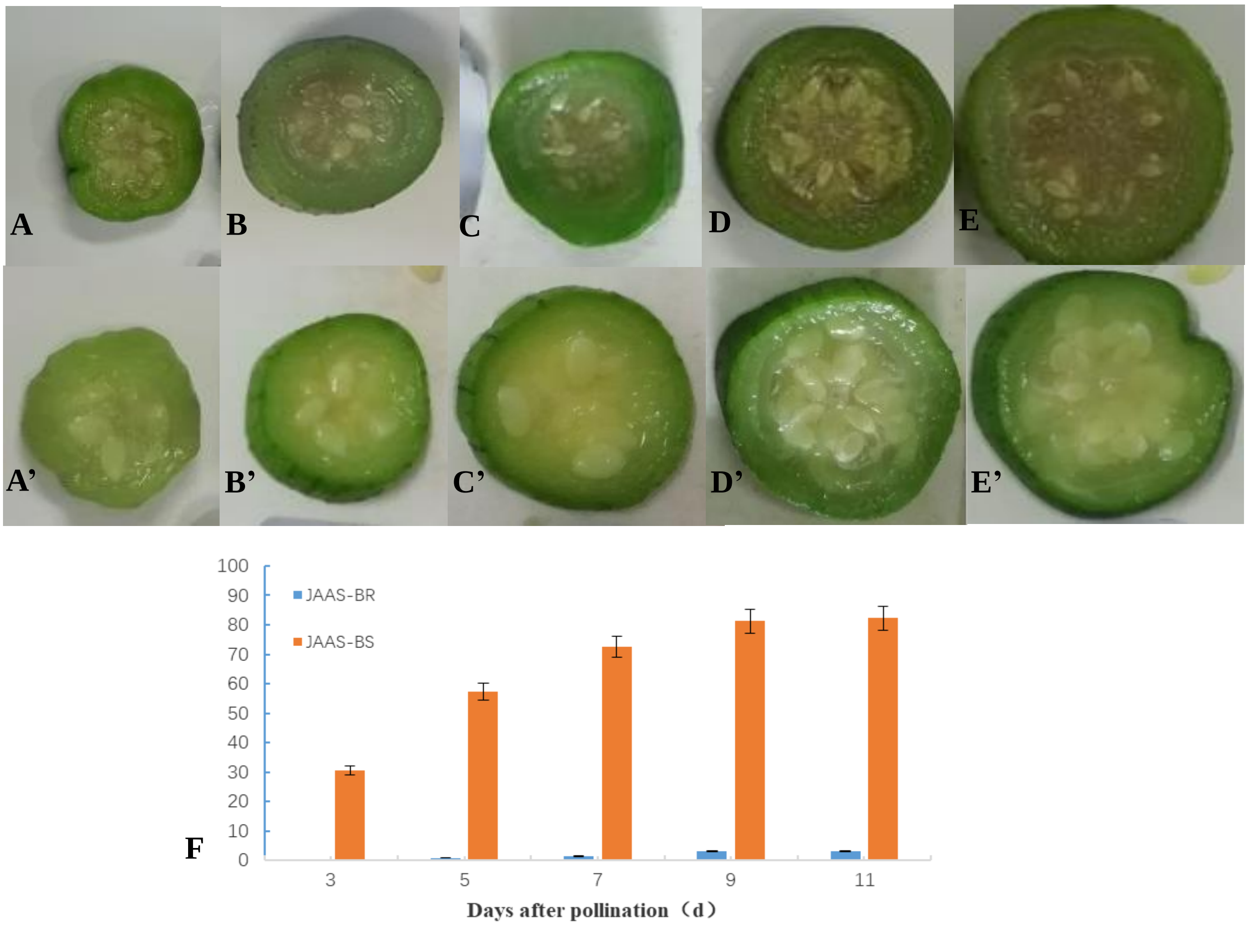

Supplementary Figure S3: Appearance and browning index at different DAP of JAAS-BS and JAAS-BR.

A-E successively represents 3, 5, 7, 9 and 11 DAP of JAAS-BS. A'-E' successively represents 3, 5, 7, 9 and 11 DAP of JAAS-BR. F represents browning index (BI) of JAAS-BS and JAAS-BR.
