# Supplementary material for: Identification of browning-related microRNAs and their targets reveals complex miRNA-mediated browning regulatory networks in Luffa cylindrica
Source: Sci Rep. 2018 Nov 2;8:16242. doi: 10.1038/s41598-018-33896-9 (PMC6214963; doi:10.1038/s41598-018-33896-9)
Supplement: Supplementary file 2 — Supplementary Table S1 [file 41598_2018_33896_MOESM2_ESM.pdf]

Identification of browning-related microRNAs and their targets reveals complex miRNA-mediated browning regulatory networks in *Luffa cylindrica*

Yuanyuan Xu, Zhe Liu, Lina Lou, Xiaojun Su*

Supplementary Table S1: Primers of miRNAs and targets in *Luffa* for RT-qPCT.

miRNA/Target	Primer sequences
miR169b	TAGCCAAAAATGACTTGCCTG
miR169t	TGAGCCAAGAATGACTTGCCGGC
miR171d	TTGAGCCGCGCCAATATCAC
miR172a-3p	AGAATCTTGATGATGCTGCGT
miR396b-3p	GTTCAATAAAGCTGTGGGAAA
miR399h	TGCCAAAGGAGACTTGCCCAG
miR477a	CTCTCCCTCAAGGGCTTCTG
miR858	TCTCGTTGTCTGTTCGACCTT
Lc-miRn10-3p	TCATGTGCCCCTCTTCGCCATC
Lc-miRn19-3p	CATGCACTGCCTCTTCCCTGGC
Lc-miRn24-5p	CATAAAATCAGAATCAAACA
Lc-miRn41-5p	CTATCGCTGATAGACTCGTATT
Lc-miRn52-5p	CTCTCTTGAATGATGACTATA
Lc-miRn54-3p	TCATGTGCCCCTCTTCTCCATC
Lc-miRn60-3p	TAGGTGATTCGTGGAAGGATTT
Lc-miRn66-3p	CACGTGCTCCCCTTCTCCAAC
NFYA-TR39930-F	TATGGAACCGAAGAAAGTC
NFYA-TR39930-R	TGCTTGATACCTCCGAAC
TOE3-TR41282-F	TTGAGAATGATAATGGTAG
TOE3-TR41282-R	GAGAGTAATTGTGAAGAT
RAP2-7-TR76908-F	CGGTCCATGTTATGCTAA
RAP2-7-TR76908-R	TCTGTTACGCTTTCCTCA
PHYH-TR37331-F	GCAGGTTATCTCGTCATTG
PHYH-TR37331-R	CAGAGGAAGTGGAGCAGTC
RGLG-TR79721-F	GAATCTTATTGTCGGTATAG
RGLG-TR79721-R	TAGCAAGGAATCATATTATC
UBC24-TR71171-F	AACAATGAGTGCTTATGAAC
UBC24-TR71171-R	CTTATCCAATGCCAACCTA
ACL5-TR32830-F	TGCTGACTGCTGGGTTCTA
ACL5-TR32830-R	AAGGTTCGCTCATGGACAA
Myb4-TR9920-F	CATCCACCTTCGCAATCA
Myb4-TR9920-R	TGTCATCCTTGTCGTTCTTAG
MYB308-TR28685-F	AGAGAATCAGAAGGAACT
MYB308-TR28685-R	TCAACCTCATCAATCATC
ODO1-TR36521-F	AGTTCCTTGTGCTTCTTCT
ODO1-TR36521-R	ATTGGCTGCTGATTCTGA
TCB3-TR53813-F	GATTCGCAACTTCCTCTC
TCB3-TR53813-F	GAACAAGCCAAGAACCAT
SR34A-TR73357-F	CGAGCAGCAGTAATGGAA
SR34A-TR73357-R	CCGATTGAAGAAGTTGATTATGAT
Actin-F1	GTACAACTGGTATCGTGCTG
Actin-R1	AGGTCCAAACGGAGAATT
