# Supplementary material for: Identification of browning-related microRNAs and their targets reveals complex miRNA-mediated browning regulatory networks in Luffa cylindrica
Source: Sci Rep. 2018 Nov 2;8:16242. doi: 10.1038/s41598-018-33896-9 (PMC6214963; doi:10.1038/s41598-018-33896-9)
Supplement: Supplementary file 3 — Supplementary Table S2 [file 41598_2018_33896_MOESM3_ESM.pdf]

Identification of browning-related microRNAs and their targets reveals complex miRNA-mediated browning regulatory networks in *Luffa cylindrica*

Yuanyuan Xu, Zhe Liu, Lina Lou, Xiaojun Su*

Supplementary Table S2: Distribution of small RNAs among different categories in JAAS-BR and JAAS-BS libraries.

Category	JAAS-BR		JAAS-BS	
	**Unique**	**Total**	**Unique**	**Total**
Total sRNAs	5,856,286	110,533,85	5,739,233	10,382,404
miRNA	8,448	221,571	7,669	186,919
rRNA	314,503	1,974,652	405,776	2,260,428
snRNA	4,372	9,228	4,687	8,811
snoRNA	11,970	46,485	11,089	43,460
tRNA	30,642	474,383	29,676	358,969
Unannotated	5,486,351	8,181,631	5,280,336	7,394,420
