# Supplementary material for: Identification of browning-related microRNAs and their targets reveals complex miRNA-mediated browning regulatory networks in Luffa cylindrica
Source: Sci Rep. 2018 Nov 2;8:16242. doi: 10.1038/s41598-018-33896-9 (PMC6214963; doi:10.1038/s41598-018-33896-9)
Supplement: Supplementary file 4 — Supplementary Table S3 [file 41598_2018_33896_MOESM4_ESM.pdf]

Identification of browning-related microRNAs and their targets reveals complex miRNA-mediated browning regulatory networks in *Luffa cylindrica*

Yuanyuan Xu, Zhe Liu, Lina Lou, Xiaojun Su*

Supplementary Table S3: Detailed information of known miRNAs identified in *Luffa* from JAAS-BR and JAAS-BS libraries.

						JAAS-BR		JAAS-BS	
**Members**	**Sequences (5'-3')**	**Size**	**Arm**	**LP**	**MFE**	**Counts**	**Normalised**	**Counts**	**Normalised**
**Conserved**									
miR156b-3p	GCTCACTTCTCTCTCTGTCACC	22	3p	205	-102.9	0	0	2	0.4455
miR156f-3p	TGCTCACTGCTCTTTCTGTCAGA	23	3p	177	-71.3	1	0.2152	0	0
miR156c	TGTCAGAAGAGAGTGAGCAC	20	5p	97	-45.4	1	0.2152	1	0.2227
miR156q	TGACAGAAGAGAGTGAGCACT	21	5p	105	-50.8	2	0.4304	1	0.2227
miR156z	ATTGGAGTGAAGGGAGCT	18	3p	187	-82.5	2	0.4304	0	0
miR156a	TGACAGAAGAGAGTGAGTAC	20	5p	130	-58.71	1	0.2152	4	0.8909
miR156i-3p	TGCTCACTTCTCTTTCTGTCATC	23	3p	93	-43.1	0	0	1	0.2227
miR156e-5p	TGATAGAAGAGAGTGAGCAC	20	5p	105	-60.2	0	0	1	0.2227
miR156d-3p	GCTCTCTATGCTTCTGTCATCA	22	3p	93	-42.99	9	1.9369	8	1.7818
miR156e	TGACAGAGGAGAGTGAGCAC	20	5p	108	-48.2	0	0	1	0.2227
miR156a-3p	GCTCACTTCTCTCTCTGTCAGT	22	3p	152	-79.9	21	4.5193	20	4.4546
miR156e-3p	GCTCACTGCTCTCTCTGTCATC	22	3p	126	-78.8	1	0.2152	0	0
miR156k-3p	GCTCGCTTCTCTTTCTGTCAGC	22	3p	123	-72.5	1	0.2152	0	0
miR157d-3p	GCTCTCTATGCTTCTGTCATC	21	3p	256	-86.92	1	0.2152	1	0.2227
miR159b-5p	GAGCTCCTTGAAGTTCAATGG	21	5p	196	-85.6	1	0.2152	1	0.2227
miR159b-3p	ATTGGAGTGAAGGGAGCTCCA	21	3p	186	-85.8	0	0	1	0.2227
miR159c	TTTGGTTTGAAGGGGGCTCTG	21	3p	163	-57.6	1	0.2152	0	0
miR159e-5p	GAGCTCCTTGAAGTCCAATT	20	5p	211	-108.04	37	7.9626	40	8.9092
miR159b	TTTGGATTGAAGGGAGCTCTC	21	3p	187	-92.3	6	1.2912	6	1.3364
miR159a	CTTGGATTGAAGGGAGCTCC	20	3p	169	-96.3	432	92.9689	354	78.846
miR159d	ATTGGATTGAAGGGAGCTCCG	21	3p	189	-100.1	2	0.4304	5	1.1136
miR159f	CTTGGATTGAAGGGAGCTCTA	21	3p	188	-95.5	491	105.666	342	76.1733

miR159	TTTGGACTGAAGGGAGCTCTA	21	3p	71	-18.8	1	0.2152	0	0
miR159e	TTTGGATTGAAAGGAGCTCTT	21	3p	264	-108	0	0	1	0.2227
miR159e-3p	ATTGGTTTGAAGGGAGCTCCA	21	3p	127	-58	2	0.4304	0	0
miR159h-3p	TTTGGAGTGAAGGGAGCTCTG	21	3p	200	-93.3	85	18.2925	62	13.8092
miR160a	TGCTCGGCTCCCTGTATGCCA	21	5p	88	-48.6	0	0	1	0.2227
miR160h	TGCCTGGCTCCCTGTATGCCATT	23	5p	86	-50.4	26	5.5953	15	3.3409
miR160b	CGCCTGGCTCCCTGTATGCCA	21	5p	103	-42.5	2	0.4304	1	0.2227
miR160g	TGCCTGGCTCCCTGTATGCCATC	23	5p	86	-53.1	1	0.2152	0	0
miR162a-5p	GGAGGCAGCGGTTCATCGATC	21	5p	228	-70.96	128	27.5463	69	15.3683
miR162	TTGATAAACCTCTGCATCCAG	21	3p	111	-42	116	24.9639	90	20.0456
miR162a	TCGATAAACCTGTGCATCCAG	21	3p	114	-40.9	0	0	1	0.2227
miR162b	TCGATAAGCCTCTGCATCCAG	21	3p	134	-52.7	9	1.9369	6	1.3364
miR164b-3p	CATGTGCCCATCTTCACCATC	21	3p	153	-65.5	31	6.6714	20	4.4546
miR164f	TGGAGAAGAAGGGCACATGCA	21	5p	161	-106.3	0	0	1	0.2227
miR164e-3p	CACGTGCTCCCCTCCTCCAAC	21	3p	114	-53.8	1	0.2152	1	0.2227
miR164b	TGGAGAGGCAGGGCACATGCT	21	5p	89	-49.9	27	5.8106	21	4.6773
miR164a	TGGAGAAGCAGGGCACGTGAA	21	5p	160	-38.7	1	0.2152	2	0.4455
miR164c	TGGAGAAGCAGGGTACGTGCA	21	5p	119	-55.32	0	0	2	0.4455
miR164h-5p	TGGAGAAGCAGGGCACGTGTG	21	5p	219	-114.8	24	5.1649	20	4.4546
miR166e	GGACCAGGCTTCATTCCCC	19	3p	88	-35.32	4	0.8608	2	0.4455
miR166	TCGGACCAGGCTTCATTCCCCC	22	5p	414	-122.21	2	0.4304	1	0.2227
miR166h-3p	TCTCGGACCAGGCTTCATTCC	21	3p	190	-76.3	2	0.4304	0	0
miR166i-5p	GGAATGTCGTCTGGTTCGAG	20	5p	97	-58	0	0	1	0.2227
miR166u	TCTCGGACCAGGCTTCATTC	20	3p	141	-47	15	3.2281	8	1.7818
miR166i	TCGGACCAGGCTTCATTCTC	20	3p	101	-44.6	686	147.6311	562	125.1736
miR166a-5p	GGACTGTTGTCTGGCTCGAGG	21	5p	170	-68	6	1.2912	3	0.6682

miR166j	TCCGGACCAGGCTTCATTCCC	21	3p	131	-50.7	1	0.2152	0	0
miR166m	CGGACCAGGCTTCATTCCCC	20	3p	176	-44.3	29	6.241	35	7.7955
miR166c	CCGGACCAGGCTTCATCCCAG	21	5p	164	-66.3	3	0.6456	1	0.2227
miR166n	TCGGACCAGGCTTCATTCCTT	21	3p	331	-87.46	28	6.0258	14	3.1182
miR166k	TCGGACCAGGCTTCATTCCT	20	3p	103	-53.8	105	22.5966	68	15.1456
miR166c-5p	GGAACGTTGGCTGGCTCGAGG	21	5p	91	-42.8	15	3.2281	9	2.0046
miR166a	TCGGACCAGGCTTCATTCC	19	3p	130	-45.41	6	1.2912	9	2.0046
miR167b-3p	GGTCATGCTCTGACAGCCTCACT	23	3p	195	-61.06	4	0.8608	1	0.2227
miR167a	TGAAGCTGCCAGCATGATCTAA	22	5p	132	-54.9	3	0.6456	6	1.3364
miR167d	TGAAGCTGCCAGCATGATCT	20	5p	90	-37.1	2	0.4304	2	0.4455
miR167	TCAAGCTGCCAGCATGATCTA	21	5p	68	-31.5	3	0.6456	1	0.2227
miR167f-3p	AGATCATGTGGCAGTTTCACC	21	3p	87	-39.6	1	0.2152	2	0.4455
miR167h-5p	TGAAGCTGCCAACATGATCTG	21	5p	91	-41.14	2	0.4304	0	0
miR167b	TGAAGCTGACAGCATGATCTA	21	5p	90	-33.1	1	0.2152	0	0
miR167j-3p	GATCATGTGGCAGTTTCATT	20	3p	109	-47.7	1	0.2152	0	0
miR167c	TCAGATGAAGCTGCCAGCATGA	22	5p	102	-42.4	2	0.4304	4	0.8909
miR168b	TCGCTTGGTGCAGGTCGAGAA	21	5p	130	-47.6	1	0.2152	2	0.4455
miR168a	TCGCTTGGTGCAGGTCGGGAC	21	5p	168	-70.6	4	0.8608	0	0
miR168a-3p	CCTGCCTTGCATCAACTGAAT	21	3p	159	-67.2	26	5.5953	17	3.7864
miR169b	TAGCCAAAAATGACTTGCCTG	21	5p	365	-106.2	154	33.1417	70	15.591
miR169q	TGAGCCAAAGATGACTTGCCT	21	5p	137	-55.39	0	0	1	0.2227
miR169d	TAGCCAAGGATGAATTGCCGG	21	5p	103	-46.02	0	0	1	0.2227
miR169k	GAGCCAAGGATGAATTGCCGG	21	5p	150	-73.02	3	0.6456	4	0.8909
miR169a	GAGCCAAGAATGACTTGTCGG	21	5p	109	-48.5	1	0.2152	0	0
miR169t	TGAGCCAAGAATGACTTGCCGGC	23	5p	75	-38.6	375	80.7021	170	37.8639
miR169n	TGAGTCAAGAATGACTTGCCG	21	5p	142	-76.7	7	1.5064	4	0.8909

miR169v	AAGCCAAGGATGAATTGCCGG	21	5p	96	-42.2	13	2.7977	6	1.3364
miR171e	TGAATGAACCGAGCCAACATC	21	3p	102	-30.99	1	0.2152	0	0
miR171	TTGAGCCGCGTCAATATCTCC	21	5p	371	-116.74	0	0	2	0.4455
miR171-5p	TGTTGGCTCGACTCACTCAGA	21	5p	173	-93.3	1	0.2152	0	0
miR171a	TGATTGAGTCGTGCCAATATC	21	3p	114	-40.2	1	0.2152	1	0.2227
miR171c	TGATTGAGCCGTGCCAATATT	21	3p	105	-36.7	1	0.2152	2	0.4455
miR171c-5p	GGATATTGGTGCGGTTCAATC	21	5p	99	-51.5	1	0.2152	0	0
miR171d-5p	TGTTGGCCCGGCTCACTCAGA	21	5p	135	-56.2	9	1.9369	16	3.5637
miR171d	TTGAGCCGCGCCAATATCAC	20	3p	112	-44	30	6.4562	13	2.8955
miR172f	TGAATCTTGATGATGCCGCAC	21	3p	163	-40.06	1	0.2152	0	0
miR172a	GCGGCATTATCAAGATTCACA	21	5p	162	-70.47	0	0	1	0.2227
miR172b	GCAGCATTATCAAGATTCACA	21	5p	119	-64	1	0.2152	0	0
miR172j	GCAGCATCATCAAGATTCCCA	21	5p	165	-69.4	1	0.2152	0	0
miR172d-5p	CAGCACCATCAAGATTCACA	20	5p	74	-34.6	0	0	1	0.2227
miR172a-5p	GCAGCACCACCAAGATTCACA	21	5p	153	-66.23	1	0.2152	1	0.2227
miR172b-5p	GCAGCACCATCAAGATTCACA	21	5p	146	-52.21	45	9.6843	30	6.6819
miR172a-3p	AGAATCTTGATGATGCTGCGT	21	3p	143	-67.1	6	1.2912	0	0
miR173-3p	TGATTCTCTGTGTAAGCGAAA	21	3p	102	-43.07	0	0	1	0.2227
miR319a-5p	AGAGCTTCCTTCAGTCCACTC	21	5p	207	-87.1	1	0.2152	1	0.2227
miR319f	TTGGACTGAAGGGGCCTCTT	20	3p	95	-28.1	0	0	1	0.2227
miR319p	TTTTGGACTGAAGGGAGCTCC	21	3p	79	-38.4	33	7.1018	25	5.5682
miR319q	TGGACTGAAGGGAGCTCCTTC	21	3p	184	-74.1	1716	369.293	1251	278.6338
miR319a	CTTGGACTGAAGGGAGCTCC	20	3p	169	-98.8	1	0.2152	1	0.2227
miR319c	CTTGGACTGAAGGGAGCTCCC	21	3p	189	-99.1	7	1.5064	2	0.4455
miR319	TTGGACTGAAGGGAGCTCC	19	3p	206	-87	58	12.4819	35	7.7955
miR319e	TTGGACTGAAGGGAGCTCCT	20	3p	192	-92.1	11	2.3673	6	1.3364

miR319i	TTGGGCTGAAGGGAGCTCCC	20	3p	89	-33	41	8.8234	35	7.7955
miR319c-5p	AGAGCTTCCTTCAGCCCACTC	21	5p	195	-81.9	0	0	4	0.8909
miR390b-3p	CGCTATCCATCCTGAGTTCCA	21	3p	127	-51	5	1.076	5	1.1136
miR390-3p	CGCTGTCCATCCTGAGTTTCA	21	3p	105	-50.2	2	0.4304	3	0.6682
miR390a-3p	CGCTATCCATCCTGAGTTTC	20	3p	105	-44.44	96	20.6597	70	15.591
miR390b-5p	AAGCTCAGGAGGGATAGCACC	21	5p	103	-39.2	1	0.2152	1	0.2227
miR390d-3p	CGCTATCCATCCTGAGTTTTA	21	3p	106	-44.7	0	0	4	0.8909
miR393a-3p	ATCATGCTATCTCTTTGGATT	21	3p	133	-47	0	0	1	0.2227
miR393b-3p	ATCATGCGATCTCTTTGGATT	21	3p	160	-57.9	0	0	1	0.2227
miR394-3p	AGGTGGGCATACTGCCAATGG	21	3p	115	-56.4	1	0.2152	0	0
miR394a	TTGGCATTCTGTCCACCTCC	20	5p	117	-53.1	1	0.2152	1	0.2227
miR394a-3p	CTGTTGGTCTCTCTTTGTAA	20	3p	147	-80.3	1	0.2152	0	0
miR395c-3p	CTGAAGTGTTTGGGGGGACTT	21	3p	166	-49.51	0	0	1	0.2227
miR395d-5p	GTTCTCCCGAACACTTCATTG	21	5p	140	-56	0	0	1	0.2227
miR395b-3p	AAGTGTTTGGGGGAACTC	18	3p	214	-79.6	4	0.8608	10	2.2273
miR395e-5p	GTTCCTTACAAGCACTTCACG	21	5p	84	-45.4	1	0.2152	1	0.2227
miR395d	TGAAGTGTTTGGGGGAACTTT	21	3p	121	-50.2	4	0.8608	1	0.2227
miR395g	TTGAAGTGTTTGGGGGAACTC	21	3p	63	-35.1	1	0.2152	0	0
miR395a	GTGAAGTGCTTGGGGGAACTC	21	3p	142	-69.2	1	0.2152	0	0
miR395f	GTGAATTGTTTGGGGGAACTC	21	3p	82	-36	6	1.2912	2	0.4455
miR396d	CTCCACGGCTTTCTTGAACTT	21	5p	229	-68.6	17	3.6585	14	3.1182
miR396c	TTCAAGAAAGCTGTGGGAAAA	21	3p	124	-59.2	60	12.9123	40	8.9092
miR396h	TCCACAGCTTTCTTGAACTG	20	5p	148	-58.2	2909	626.0334	3827	852.3833
miR396c-3p	GGTCAAGAAAGCTGTGGGAAG	21	3p	141	-68.8	56	12.0515	36	8.0182
miR396g	TTCTTGAACTTCTTATGCATC	21	3p	151	-46.88	0	0	3	0.6682
miR396e-3p	CTCAAGAAAGCTGTGGGAGA	20	3p	145	-58.4	1	0.2152	0	0

miR396g-3p	CTCAAGAAAGCCGTGGGAAAA	21	3p	168	-66.2	7	1.5064	7	1.5591
miR396	TCCCACGGCTTTCTTGAACTT	21	5p	107	-46.7	10	2.1521	3	0.6682
miR396-3p	AAGCTCAAGAAAGCTGTGGGA	21	3p	95	-38.1	2380	512.1896	2166	482.4307
miR396a	TTCCACAGCTTTCTTGAACGT	21	5p	98	-39	91	19.5837	107	23.832
miR396b-3p	GTTCAATAAAGCTGTGGGAAA	21	3p	136	-69.9	68	14.634	30	6.6819
miR397b-3p	TCAGCGTTGCATTCAATTATG	21	3p	131	-49.04	10	2.1521	3	0.6682
miR398f	GGTGTTCTCAGGTCGCCCCTG	21	3p	88	-37.2	81	17.4317	73	16.2592
miR398b-5p	GGGGCGGACTGGGAACACATG	21	5p	108	-61.8	91	19.5837	69	15.3683
miR398	TGTGTTCCCAGGTCGCCCCTG	21	3p	185	-74.92	2	0.4304	0	0
miR399g	AGGGCTTCTCTCCATTGGCAGG	22	5p	69	-33.8	185	39.8131	143	31.8502
miR399h	TGCCAAAGGAGACTTGCCCAG	21	3p	99	-59.84	1761	378.9772	700	155.9102
miR399a	CGCCAAAGGAGAGTTGCCCTT	21	3p	109	-55	30	6.4562	29	6.4591
miR399e	CGCCAAAGGAGAGTTGCCCTC	21	3p	109	-40.12	3	0.6456	2	0.4455
miR399i	CGCCAAAGGAGAGTTGCCCTG	21	3p	122	-57.9	0	0	3	0.6682
miR399e-5p	GGGCTTCTCTTTCTTGGCAGG	21	5p	124	-58.3	5	1.076	6	1.3364
miR408	TGCACTGCCTCTTCCCTGGCTG	22	3p	109	-53	3	0.6456	0	0
miR408e	CTGCACTGACTCTTCCCTGGC	21	3p	283	-105.5	1	0.2152	0	0
miR482a-5p	AGGAATGGGCTGTTTGGGAAGA	22	5p	155	-93.8	0	0	1	0.2227
miR482d	AATGGAAGGGTAGGAAAGAAG	21	5p	106	-45.9	0	0	1	0.2227
miR482e-5p	TGTGGGTGGGGTGGAAAGATT	21	5p	188	-77	3	0.6456	0	0
miR482-3p	TCTTCCTTGTTCCTCCCATT	20	3p	122	-38	1	0.2152	0	0
miR535b	TGACAAGGAGAGAGAGCACGC	21	5p	95	-61.4	1	0.2152	0	0
**Non-Conserved**									
miR403c-5p	TTTGTGCGTGGATCTGAGGCC	21	5p	79	-41.42	0	0	1	0.2227
miR414	TCATCTTCATCATCATCGTCA	21	5p	108	-23.36	2	0.4304	0	0
miR477e	CTCTCCCTCAAGGGCTTCTA	20	5p	84	-36.4	5	1.076	4	0.8909

miR477a	CTCTCCCTCAAGGGCTTCTG	20	5p	79	-29.1	43	9.2538	20	4.4546
miR477i	ACTCTCCCTCAAGGGCTTCCG	21	5p	107	-62.4	3	0.6456	3	0.6682
miR477b	TCTCTCCCTCAAGGGCTTCTC	21	5p	133	-51.3	37	7.9626	20	4.4546
miR477h	ACTCTCCCTCAAGGGCTTCAG	21	5p	143	-71.6	0	0	1	0.2227
miR477a-5p	CCTCTCCCTCAAGGGCTTCTC	21	5p	68	-35.7	4	0.8608	2	0.4455
miR529	AGAAGAGAGAGAGCACAGCTT	21	3p	120	-46.1	0	0	2	0.4455
miR827	TTAGATGACCATCAACGAACA	21	3p	181	-66	138	29.6984	84	18.7092
miR845b-5p	TCAATTGGTATCAGAGCAACG	21	5p	112	-42	1	0.2152	2	0.4455
miR845a	CGGCTCTGATACCAATTGATG	21	3p	163	-58.3	35	7.5322	28	6.2364
miR845a-3p	CGGCTCTGATACCAGTTGATG	21	3p	115	-55.9	18	3.8737	18	4.0091
miR845c	AGGCTCTGATACCAATTGATG	21	3p	90	-30.3	6	1.2912	10	2.2273
miR845d	TGGCTCTGATACCAATTGATG	21	3p	131	-32.25	9	1.9369	9	2.0046
miR854	GATGAGGATAGTGAGGAGGAG	21	5p	592	-145.1	1	0.2152	0	0
miR858	TCTCGTTGTCTGTTCGACCTT	21	5p	164	-27.5	156	33.5721	18	4.0091
miR894	CGTTTCACGTCGGGTTCACC	20	5p	220	-36	226	48.6365	184	40.9821
miR1511	AACCAGGCTCTGATACCATG	20	3p	99	-38.6	62	13.3428	53	11.8046
miR1511-3p	ACCTGGCTCTGATACCATAAC	21	3p	84	-38.2	18	3.8737	8	1.7818
miR1863	AGCTCTGATACCATGTTAGATTTG	24	3p	605	-173	39	8.393	32	7.1273
miR1863a	AGCTCTGATACCATGTTAGATTAG	24	3p	374	-127.61	0	0	4	0.8909
miR2111a	TAATCTGCATCCTGAGGTTTG	21	5p	110	-49.4	56	12.0515	67	14.9228
miR2111-3p	GTCCTCTGGTTGCAGATTACT	21	3p	108	-44.9	0	0	1	0.2227
miR2111b	ATCCTCGGGATACAGATTACC	21	3p	119	-51.7	1	0.2152	0	0
miR2118b	TTCCCGATGCCTCCCATTCCTA	22	3p	179	-87.6	1	0.2152	0	0
miR3630-5p	TGCAAGTGACGATATCAGACA	21	5p	102	-36.9	1	0.2152	1	0.2227
miR5021	TGAGAAGAAGAAGAAGAAAA	20	5p	171	-50.5	3	0.6456	5	1.1136
miR5293	GATGAAGAAGTGGAAGGAAGAAGA	24	5p	121	-39.4	1	0.2152	0	0

miR5298a	TGGATATGATATGAAGATGAAGAA	24	3p	92	-24.4	0	0	1	0.2227
miR5654a	ATAAATCCCAAGCATCATCCA	21	5p	195	-73.11	1	0.2152	0	0
